# Automated Glycan Assembly of ^19^F‐labeled Glycan Probes Enables High‐Throughput NMR Studies of Protein–Glycan Interactions

**DOI:** 10.1002/anie.202102690

**Published:** 2021-05-07

**Authors:** Giulio Fittolani, Elena Shanina, Mónica Guberman, Peter H. Seeberger, Christoph Rademacher, Martina Delbianco

**Affiliations:** ^1^ Department of Biomolecular Systems Max Planck Institute of Colloids and Interfaces Am Mühlenberg 1 14476 Potsdam Germany; ^2^ Department of Chemistry and Biochemistry Freie Universität Berlin Arnimallee 22 14195 Berlin Germany; ^3^ Current address: Department of Pharmaceutical Chemistry University of Vienna Althanstrasse 14 1080 Vienna Austria; ^4^ Current address: Department of Microbiology, Immunobiology and Genetics Max F. Perutz Labs Campus Vienna Biocenter 5 1030 Vienna Austria; ^5^ Current address: Medicinal Chemistry Leibniz-Forschungsinstitut für Molekulare Pharmakologie Robert-Rössle Strasse 10 13125 Berlin Germany

**Keywords:** ^19^F NMR spectroscopy, automated glycan assembly, glycans, lectins, protein–glycan interactions

## Abstract

Protein–glycan interactions mediate important biological processes, including pathogen host invasion and cellular communication. Herein, we showcase an expedite approach that integrates automated glycan assembly (AGA) of ^19^F‐labeled probes and high‐throughput NMR methods, enabling the study of protein–glycan interactions. Synthetic Lewis type 2 antigens were screened against seven glycan binding proteins (GBPs), including DC‐SIGN and BambL, respectively involved in HIV‐1 and lung infections in immunocompromised patients, confirming the preference for fucosylated glycans (Le^x^, H type 2, Le^y^). Previously unknown glycan–lectin weak interactions were detected, and thermodynamic data were obtained. Enzymatic reactions were monitored in real‐time, delivering kinetic parameters. These results demonstrate the utility of AGA combined with ^19^F NMR for the discovery and characterization of glycan–protein interactions, opening up new perspectives for ^19^F‐labeled complex glycans.

## Graphical abstract

^19^F‐labeled glycans produced with automated glycan assembly allowed for the high‐throughput NMR screening of protein–glycan interactions. Binding to mammalian and bacterial lectins as well as enzymes was monitored in real‐time with a fast assay that does not require labeled proteins or complex 2D NMR experiments. Previously unknown glycan–lectin weak interactions were detected, and quantitative data were obtained.

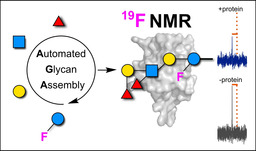

## Introduction

Glycans are a highly diverse class of biomolecules involved in several processes such as cellular communication and recognition and play important structural and modulatory roles.^[1]^ Pathogens invade the host by mimicking or exploiting host glycans present on endothelial cells. This process is often mediated by lectins, a class of glycan‐binding proteins (GBPs) expressed by both pathogens and hosts. Typically, mammalian glycans have low affinity for mammalian receptors, while showing higher affinity for bacterial lectins.^[2]^ Profiling glycan–lectin interactions is a crucial step towards the understanding of the biological functions of glycans. Still, the extreme complexity and diversity of glycans pose a severe bottleneck to the characterization of these generally weak and promiscuous interactions.

Synthetic glycans are valuable probes to dissect glycan–protein interactions. However, lengthy synthetic protocols hampered their systematic and widespread use in glycobiology. Automated glycan assembly (AGA) enables fast access to complex and well‐defined glycans.[^3^, ^4^] With AGA, glycans are typically assembled in an overnight run, permitting the production of broad collections of glycans for systematic screenings.^[5]^

An additional challenge to the study of glycan–protein interactions is the need for highly sensitive methods able to detect the often inherently low affinities. Several analytical techniques have been developed to quantitatively describe these interactions at the molecular level and in a high‐throughput manner.[^6^, ^7^, ^8^] Most of these strategies rely on immobilized glycans (e.g. microarray technology)[^6^, ^7^, ^8^, ^9^, ^10^] or require large amounts of samples and analysis time (ITC,^[11]^ SPR,^[12]^ or X‐ray crystallography^[13]^). In contrast, NMR allows for the detection of protein–glycan interactions in solution in a fast and reliable manner, providing information on the binding mode in a homogeneous assay format in absence of immobilization protocols.[^14^, ^15^]

NMR active labels are commonly introduced to simplify NMR analysis.[^15^, ^17^] Among all, the ^19^F nucleus stands out due to its unique properties such as: i) high sensitivity to local chemical environment, ii) short acquisition times, iii) simple spectra, iv) broad chemical shift range, and v) absence in biological systems (no background signal).[^18^, ^19^] Even though ^19^F NMR has enabled the description of peptide (mis)folding, real‐time in vivo events,[^18^, ^19^, ^20^, ^21^, ^22^] protein–ligand interactions, and high‐throughput ligand screening,[^23^, ^24^] the use of fluorinated glycans to investigate protein binding^[25]^ and enzymatic reactions[^26^, ^27^, ^28^] is just at the beginning. The labor‐intensive multistep synthesis of ^19^F‐labeled glycans represents the main bottleneck and has limited these studies to small collections of short and relatively simple glycans.[^14^, ^29^, ^30^, ^31^, ^32^] Still, ^19^F‐labeled glycans have the potential to dissect protein–glycan interactions.[^33^, ^34^]

Herein, we present a high‐throughput NMR‐based approach for the screening and characterization of protein–glycan interactions using ^19^F‐labeled glycans. AGA enabled quick access to a collection of ^19^F‐labeled Lewis type 2 complex glycans. Lewis type 2 antigens are involved in several physiological and pathological processes, including cancer, where they act as cell adhesion or recognition mediators.[^35^, ^36^] Subtle differences in the fucosylation pattern strongly impact their interaction with proteins and ultimately can lead to host immune system elusion.[^37^, ^38^, ^39^, ^40^] The ^19^F‐labeled glycan probes (hereafter F‐glycans) were screened against mammalian and bacterial lectins as well as enzymes. Among mammalian lectins, we selected Langerin^[41]^ and the dendritic cell specific ICAM‐3 grabbing non‐integrin (DC‐SIGN)^[42]^ both of which are known to bind high‐mannose N‐glycans. DC‐SIGN also selectively recognizes specific fucosylated glycans,^[43]^ playing a crucial role in the biology of viral pathogens (e.g. HIV). In addition, we screened soluble lectins produced by some opportunistic pathogens responsible for lung infections, such as *Pseudomonas* (LecA and LecB)^[44]^ and *Burkholderia* (BambL)^[45]^ species. Finally, we selected two different sialyltransferases and screened their interactions with Lewis antigens, given the importance and widespread occurrence of terminal sialylation in Lewis antigens.[^46^, ^47^] The labeled glycan probes in combination with ^19^F NMR proved to be valuable for detecting binding events in real‐time, identifying new weak protein–glycan interactions, and determining affinities (*K*_d_) as well as kinetics of enzymatic reactions.

## Results and Discussion

### Automated Synthesis of F‐Glycans

**Figure 1:**
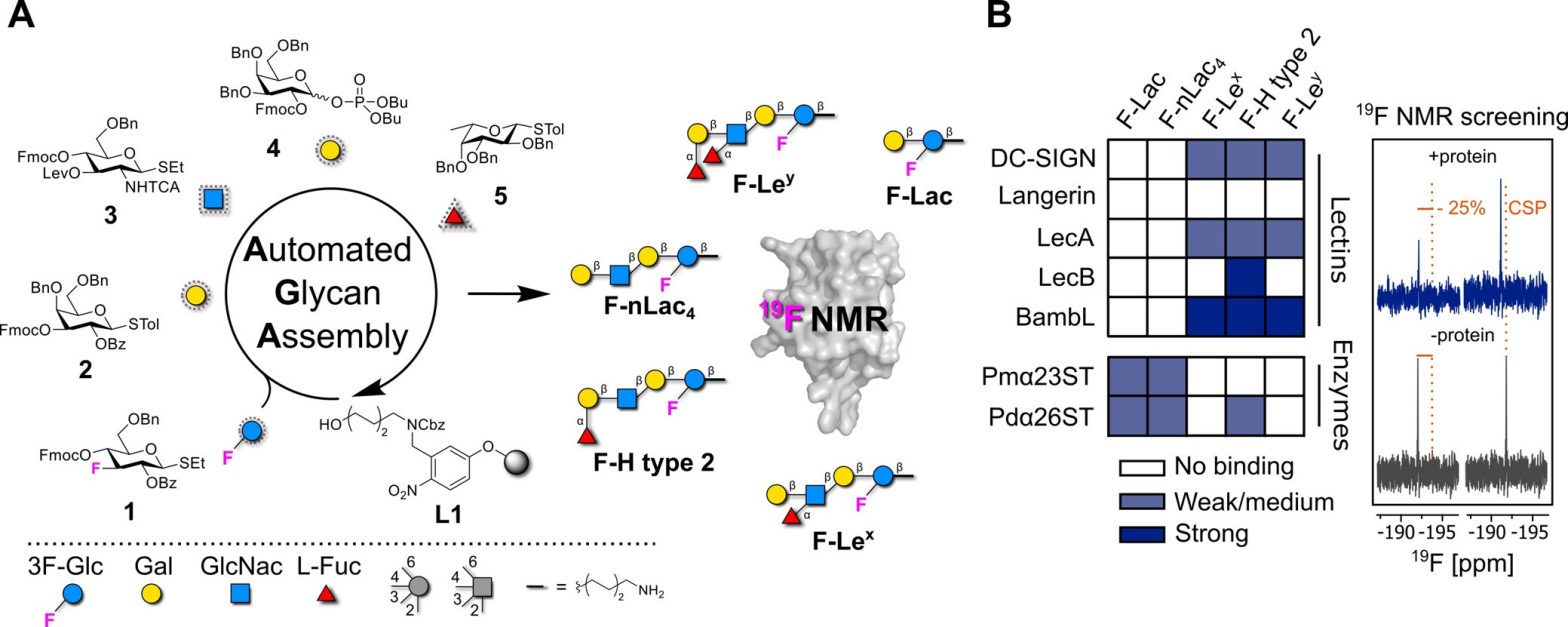
Integrated approach for the preparation of ^19^F‐labeled Lewis type 2 glycans by AGA and screening against lectins and enzymes. A) BBs **1**–**5**, including BB **1** bearing the ^19^F reporter, were employed for the AGA of a collection ^19^F‐labeled Lewis type 2 antigen analogs represented following the Symbol Nomenclature For Glycans (SNFG).^[16]^ B) The F‐glycans were screened against proteins, including mammalian and bacterial lectins, as well as enzymes. The enzymes were screened in the absence of donor (i.e. CMP‐Neu5Ac) to probe binding to the substrate. The binding strength was defined depending on the changes observed in the NMR after addition of the protein (right panel). Strong binding (blue) is defined as a decrease in peak intensity higher than −25 % or a chemical shift perturbation (CSP) bigger than 0.01 ppm in the ^19^F NMR. Weak/medium binding (light blue) is defined as a decrease in peak intensity higher than −25 % in the CPMG‐filtered ^19^F NMR. No binding (white) is defined as a decrease in peak intensity lower than −25 % in CPMG‐filtered ^19^F NMR.

Recently, an elegant procedure to access a collection of Lewis type 2 antigens by AGA was reported.^[48]^ We envisioned a similar approach to produce a set of ^19^F‐labeled analogs to screen protein binding in a simple ^19^F NMR assay. Since the position of the ^19^F reporter is thought to be crucial to obtain valuable information, F‐glycans (**F‐Lac**, **F‐nLac_4_**, **F‐Le^x^**, **F‐H type 2**, and **F‐Le^y^**) were designed with the ^19^F reporter in the lactose inner core subunit (Figure 1 A). This position is distal from the binding site (i.e. non reducing end) to minimize the effect of the fluorine atom during the binding event.[^49^, ^50^] We hypothesize that labeling of the inner core glucose unit should maintain sensitivity to the binding event due to overall changes in the correlation time of the glycan in the bound state, reporting changes in the ^19^F NMR signal.^[20]^

^19^F‐labeled analogs of Lewis type 2 antigens were assembled on a solid support (functionalized Merrifield resin, **L1**) using building blocks (BBs) **1**–**5** (Figure 1 A). The BBs are equipped with a thioether or a dibutylphosphate reactive leaving group. Orthogonal cleavage of the 9‐fluorenylmethoxycarbonyl (Fmoc) and levulinoyl (Lev) temporary protecting groups permits regioselective chain elongation. Benzyl (Bn), benzoyl (Bz), and *N*‐trichloroacetyl (TCA) groups protect the remaining functionalities. β‐Stereoselectivity during glycosylation with BBs **1**–**4** is ensured by anchimeric assistance of the protecting groups at C‐2, while α‐stereoselectivity with BB **5** was verified in previous studies.^[48]^ BB **1** is labeled with the ^19^F reporter at the C‐3 position.^[51]^ Each oligosaccharide was assembled in an overnight run following previously reported conditions for unlabeled analogs (see SI).^[48]^ Post‐AGA manipulations included solid‐phase methanolysis,^[51]^ photocleavage^[52]^ from the solid support, and hydrogenolysis (see SI). A single final purification step afforded the target F‐glycans in overall yields of 5 % to 16 % over 7 to 15 steps.

### ^19^F NMR Screening of F‐Glycan Library

A ^19^F and CPMG NMR screening was performed to probe the interactions of five F‐glycans (**F‐Lac**, **F‐n‐Lac_4_**, **F‐Le^x^**, **F‐H type 2**, and **F‐Le^y^**) with mammalian (Langerin, DC‐SIGN) and bacterial (LecA, LecB, BambL) lectins and enzymes (α(2,3)‐sialyltransferase from *Pasteurella multocida* (Pmα23ST)^[53]^ and α(2,6)‐sialyltransferase from *Photobacterium damsela* (Pdα26ST)^[54]^) (Figure 1 B). Upon protein binding, the molecular tumbling rate of the glycan is drastically affected resulting in a decrease of the ^19^F signal intensity.^[20]^ Monitoring ^19^F chemical shift perturbation (CSP) or change in peak intensity upon addition of protein allowed us to qualitatively evaluate the strength of the interaction. A decrease in peak intensity or a CSP in ^19^F NMR indicates strong binding. Application of a CPMG‐based spin echo filter allows us to detect weak binders. As a result, bacterial (LecA, LecB, and BambL) and mammalian (DC‐SIGN ECD) lectins preferred fucosylated glycans (Figures S2A, S2B, S2C, and S2E). No binding to F‐glycans was observed in presence of Langerin ECD (Figure S2D), in agreement with previous reports.^[55]^ In contrast, the enzymes showed much weaker interactions and a slight preference for shorter non‐branched glycans (Figure S3).

### Reporter Position on F‐Glycans Does Not Affect Binding to Mammalian and Bacterial Lectins

**Figure 2:**
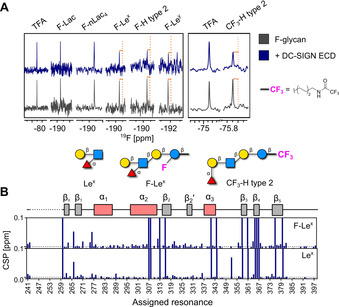
Mammalian lectin (DC‐SIGN) binding to F‐glycans and study on the reporter position. A) CPMG NMR screening of F‐glycans alone (gray) and in presence of DC‐SIGN ECD (blue). DC‐SIGN ECD binds to **F‐Le^x^**, **F‐H type 2**, and **F‐Le^y^** as shown by a decrease in peak intensity in presence of protein (orange lines, left panel). CPMG NMR spectra of **CF_3_‐H type 2** alone (gray) and in presence of DC‐SIGN ECD (blue; right panel). B) Cartoon of assigned domains of DC‐SIGN CRD (unassigned resonances in dashed line) and CSP plot of assigned resonances in presence of **F‐Le^x^** and **Le^x^** showing that **F‐Le^x^**‐perturbed resonances similarly to unlabeled **Le^x^**.

DC‐SIGN recognizes cellular ligands and pathogens that express Lewis antigens. In particular, Le^x^ and Le^y^ present on *Schistosoma mansoni* ^[56]^ and *Helicobacter pylori* ^[43]^ or endothelial cells,^[57]^ respectively, are known binding partners for DC‐SIGN.^[58]^ The strong preference of DC‐SIGN for fucosylated ligands has also been elucidated with the crystal structure of the carbohydrate‐binding site of DC‐SIGN bound to Le^x^.^[59]^ The qualitative CPMG NMR screening of mammalian lectins confirmed the interaction of DC‐SIGN with fucosylated glycans **F‐Le^x^**, **F‐H type 2**, and **F‐Le^y^** (Figure 2 A), as indicated by changes in the NMR peak intensity of the reporter molecule. This effect is maximized with a protein‐to‐ligand ratio of 2:1 (Figure S4A).

First, we explored the role of the ^19^F reporter in F‐glycan binding to DC‐SIGN. We performed protein‐observed ^15^N HSQC NMR and recorded an HSQC NMR spectrum of DC‐SIGN CRD in the presence of **F‐Le^x^** and **Le^x^**. Both ligands promoted similar changes in the backbone of DC‐SIGN CRD (Figure 2 B and S4B). Next, we investigated the effect of the reporter's position on the ability to reveal binding events. We conjugated a CF_3_ moiety to the remote end of the aminopentyl linker on **H type 2** (**CF_3_‐H type 2**), far from the carbohydrate‐binding site, and tested the new ligand in ^19^F and CPMG NMR. Remarkably, its binding was observed with both mammalian (DC‐SIGN, Figure 2 A) and bacterial lectins (BambL, Figure S5). These results indicate that the positioning of the ^19^F reporter on the Glc unit does not affect the binding of F‐glycans with proteins. Furthermore, the ^19^F reporter can be remote to the glycan binding site to avoid any interference with the binding event, while preserving excellent sensitivity. However, the functionalization of the amino linker with a CF_3_ moiety prevents any further conjugation of the glycan (e.g. to protein, surface, liposome).

**Figure 3:**
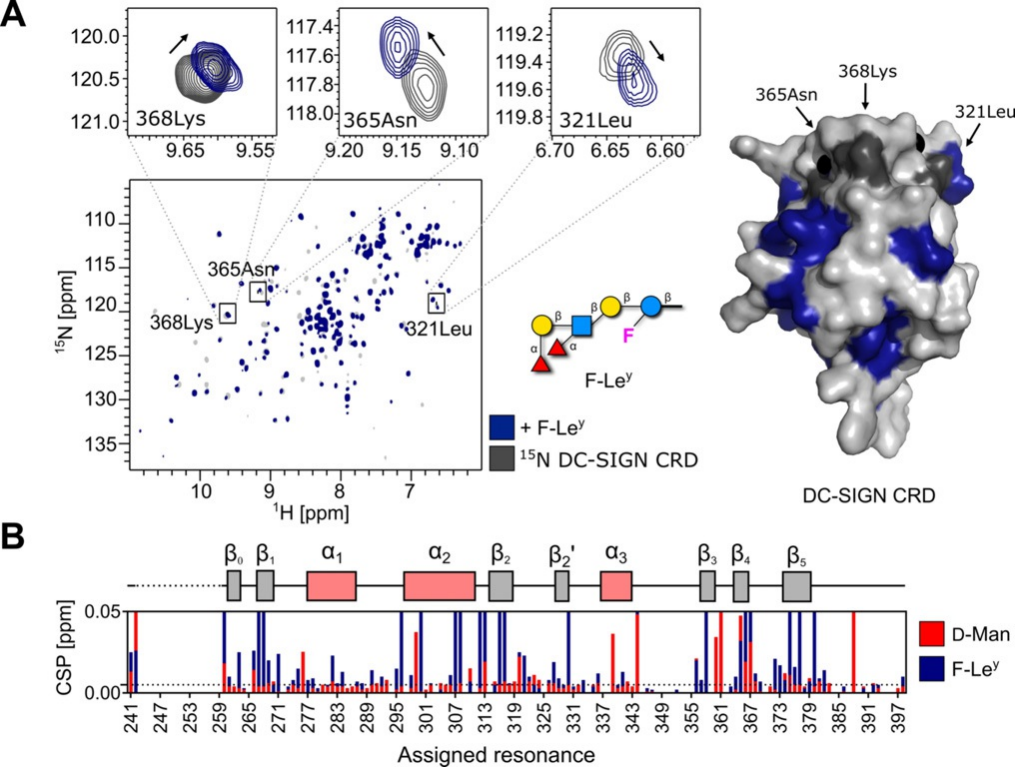
Mammalian lectin (DC‐SIGN) binding to **F‐Le^y^**. A) HSQC NMR (left) shows the interaction of **F‐Le^y^** with ^15^N‐labeled DC‐SIGN CRD and the perturbed residues were mapped on a structure of DC‐SIGN CRD (blue). Surface diagram of the crystal structure of DC‐SIGN CRD (PDB: 1sl4; right). **F‐Le^y^** targets the carbohydrate‐binding site of DC‐SIGN CRD based on changes in resonances (e.g. 321Leu, 365Asn and 368Lys, gray). B) Cartoon of assigned domains of DC‐SIGN CRD (unassigned resonances in dashed line) and CSP plot showing that **F‐Le^y^**‐perturbed resonances similarly to d‐mannose (red, positive control). The magnitude of **F‐Le^y^**‐promoted CSPs is higher compared to d‐mannose. CSPs exceeding the threshold (dashed line at 0.005 ppm) and intensities decreasing by more than 50 % were used for mapping the binding site of **F‐Le^y^** on a structure of DC‐SIGN CRD.

We further investigated the interactions of DC‐SIGN CRD with **F‐Le^y^** and **F‐H type 2** in ^15^N HSQC NMR (Figure 3 A and S6A). Even though **Le^y^** is known for its interaction with DC‐SIGN, structural data are lacking.^[57]^ Both ligands promoted CSPs of the residues located in the carbohydrate‐binding and remote sites of DC‐SIGN CRD. Binding to **F‐Le^y^** promoted larger changes in DC‐SIGN CRD than **F‐H type 2** or the monosaccharide positive control d‐mannose (Figure 3 B and S6B). This result proved that the avidity effect plays a crucial role in the interactions between DC‐SIGN and Lewis type 2 antigens, as similarly noted for high‐mannose structures.^[60]^ The CSPs observed in remote parts of the protein suggest allosteric binding, a known mechanism for C‐type lectins such as DC‐SIGN.[^61^, ^62^, ^63^] Cumulatively, we believe these probes are valuable tools for the description of the interaction mechanisms between DC‐SIGN and fucosylated blood antigens.

### Binding Affinity of F‐Glycans to Bacterial Lectins

**Figure 4:**
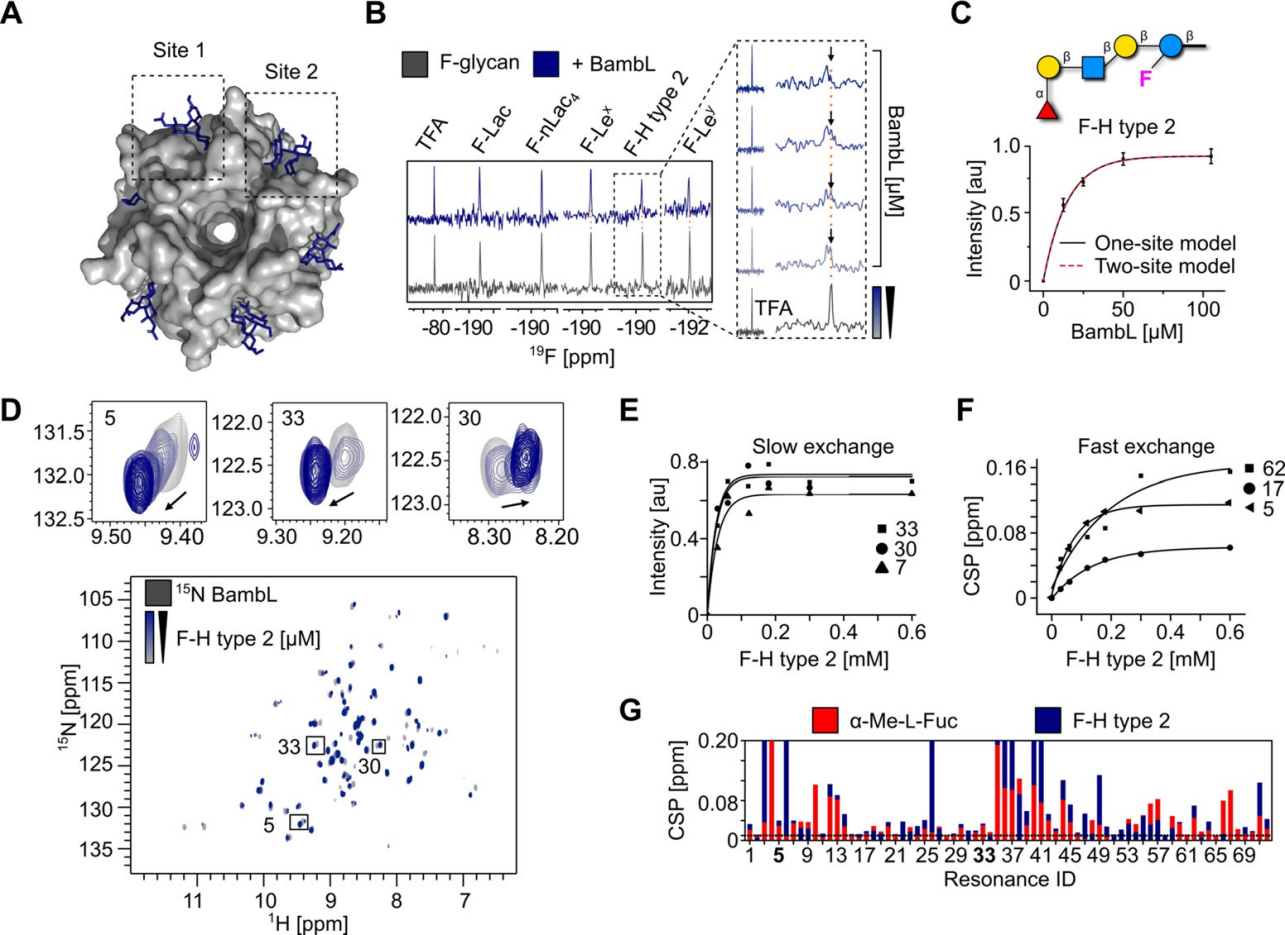
Bacterial lectin (BambL) binding to F‐glycans. A) Surface diagram of the crystal structure of BambL in complex with **H‐F type 2** (PDB: 3zzv). Sites 1 and 2 correspond to the carbohydrate‐binding sites within a monomer and between two monomers, respectively. B) ^19^F NMR screening of F‐glycans alone (gray) and in presence of BambL (blue). BambL binds **F‐Le^x^, F‐Le^y^**, and **F‐H type 2** strongly as shown by CSP in presence of protein (orange line). The ^19^F NMR titration spectra shows **F‐H type 2** undergoing slow exchange on the chemical shift timescale upon increase of BambL concentration. C) The *K*_d_ of **F‐H type 2** was calculated from the changes in peak intensity and fitted to one‐ and two‐site models resulting in a *K*_d_ of 9±2 μm. D) TROSY NMR verified **F‐H type 2** binding to ^15^N‐labeled BambL. Given that BambL has two binding sites, peaks showing a slow (30, 7, and 33), intermediate and fast exchange (5, 17, and 62) on the chemical shift timescale have been observed upon titration of **F‐H type 2**. One‐site model for slow (E) and fast exchange (F) peaks was applied to derive the *K*_d_ values of 12±8 μm and 94±33 μm, respectively. G) CSP plot showing the resonances perturbed in presence of α‐Me‐l‐fucose and **F‐H type 2**.

Bacterial lectins show a remarkably high affinity for fucosylated blood group antigens.[^35^, ^64^] The interaction of BambL from *Burkholderia ambifaria* with **H type 2** has been thoroughly investigated and two binding sites were identified in a crystal structure of the complex (Figure 4 A).^[35]^ We set on to verify this interaction for F‐glycans in ^19^F and protein‐observed NMR.

First, we performed ^19^F NMR screening and titration experiments with fucosylated F‐glycans. ^19^F NMR experiments allowed us to confirm the interaction and obtain affinity constants for **F‐H type 2** (*K*_d_=9±2 μm, Figure 4 B and 3 C) and **F‐Le^y^** (*K*_d_=14±2 μm, Figure S7A). Given that BambL has two binding sites available for glycan binding, we applied one‐ and two‐binding site models to derive the affinities for both sites. Both models resulted in matching *K*_d_ values, in agreement with values reported by ITC.^[35]^ Even though we did not observe a difference in the affinities between the two sites in ^19^F NMR, we showed that ^19^F NMR can be applied reliably to derive affinities while considerably reducing the amount of ligand needed for ITC.

We verified the interaction of **F‐H type 2** (Figure 4 D) and **F‐Le^y^** (Figure S7B) with ^15^N‐labeled BambL in protein‐observed ^15^N TROSY NMR. Changes in protein backbone similar to the one obtained with α‐Me‐l‐fucose indicate that the α‐l‐fucose branch was mainly responsible for the binding (Figures S7C and 4 D). To derive affinities, we titrated both ligands and followed the changes in peak intensities and CSPs for the peaks in slow (**F‐H type 2**: *K*_d_=12±8 μm, Figure 4 F and **F‐Le^y^**: *K*_d_=17±3 μm, Figure S7D), and fast (**F‐H type 2**: *K*_d_=94±33 μm, Figure 4 G and **F‐Le^y^**: *K*_d_=245±29 μm, Figure S7E) exchange regimes, respectively. However, protein‐observed NMR is not well suitable for the determination of *K*_d_ for ligands with high affinities and thus, it hampered the accurate derivation of the *K*_d_.^[65]^ This underscores the advantage of the ^19^F NMR ligand‐observed approach.

In addition to the known strong interactions of LecB and BambL with fucosylated glycans,^[66]^ CPMG NMR screening revealed weak interactions between LecA and fucosylated F‐glycans. To confirm this observation, we performed ^19^F R_2_‐filtered, protein‐observed ^19^F (PrOF) and ^15^N TROSY NMR experiments. **F‐H type 2** showed a faster relaxation in presence of protein, indicating a weak interaction with LecA (Figure S8B). Protein‐observed NMR experiments with 5‐fluorotryptophan (5FW, Figure S8C) and ^15^N‐labeled LecA (Figure S8D and S8E) confirmed that this interaction takes place in the canonical carbohydrate‐binding site of LecA, as indicated by perturbation of W42 and CSPs promoted in a similar manner to d‐galactose, respectively. To the best of our knowledge this is the first report of such weak binding detected using a biophysical method.[^67^, ^68^] These results demonstrate that F‐glycans serve as probes for the affinity determination and discovery of new interactions using low amounts of protein and ligand.

### Enzyme Binding and Real‐Time Kinetics with F‐Glycans

The ^19^F NMR assay allowed us to monitor the binding of F‐glycans (**F‐Lac** and **F‐nLac_4_**) to enzymes. Two sialyltransferases (Pmα23ST^[53]^ and Pdα26ST^[54]^) were screened in the absence of donor (i.e. CMP‐Neu5Ac) and revealed weak binding to the glycan substrate (Figure 1 B and S3). This is particularly relevant because binding sites of transferases usually have a very low affinity for the acceptors, making these interactions difficult to detect. Shorter non‐branched glycans (**F‐Lac** and **F‐nLac_4_**) showed stronger binding than longer branched ones. **F‐Le^x^** did not show any binding with Pmα23ST or Pdα26ST, matching its known poor reactivity as acceptor (Figure S3).^[69]^ In contrast, Pdα26ST showed weak binding to **F‐H type 2**, in agreement with previously reported enzymatic activity (Figure S3).^[54]^ This simple assay could be envisioned as screening platform to identify acceptor substrates for known enzymes and for the discovery of new glycosyltransferases.[^70^, ^71^]

**Figure 5:**
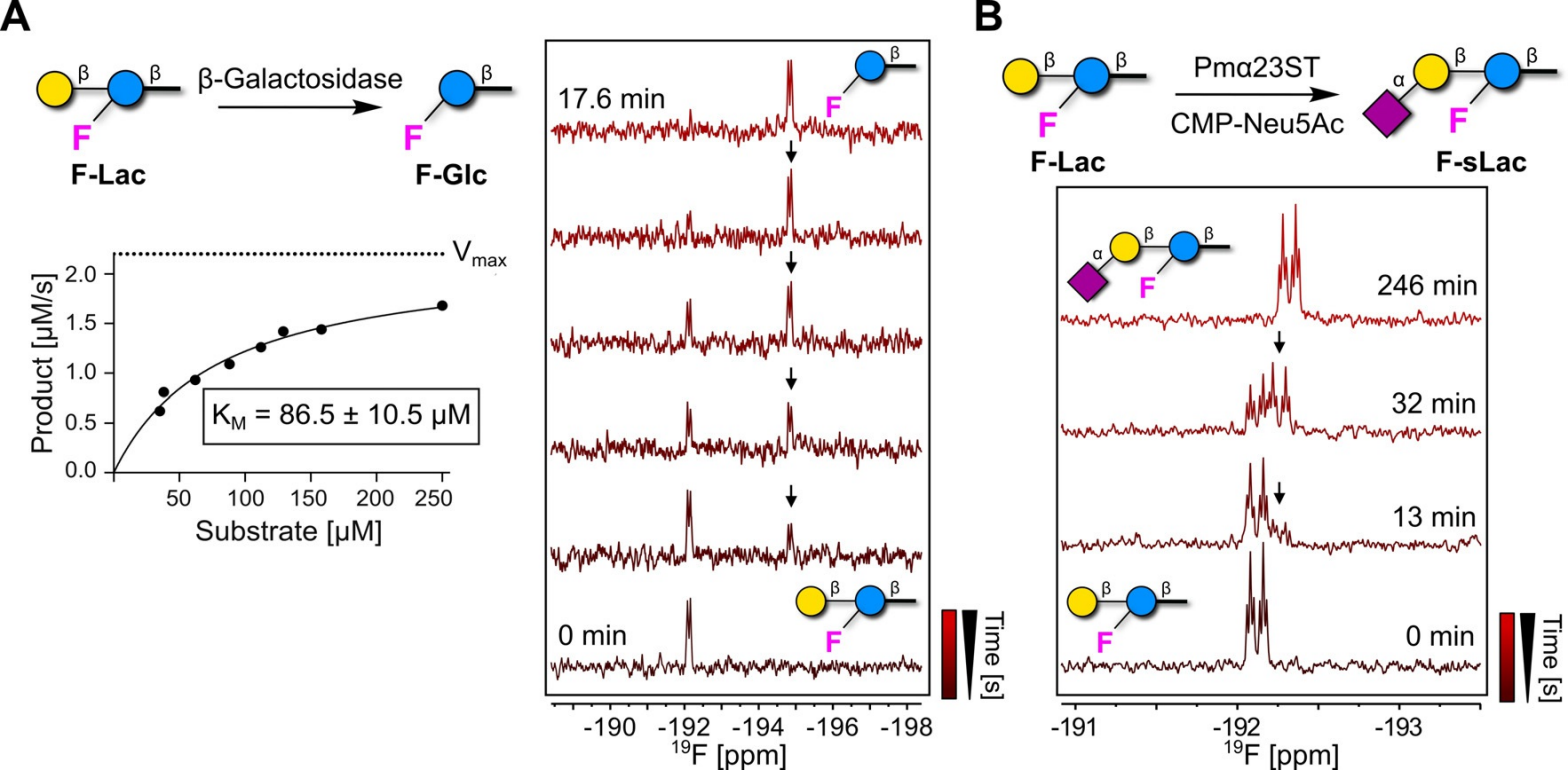
Real‐time enzyme kinetics by ^19^F NMR using F‐glycans. A) ^19^F NMR of **F‐Lac** incubated with β‐galactosidase. ^19^F NMR real‐time tracking of product formation (black arrows) upon incubation of **F‐Lac** with β‐galactosidase (right). Kinetic data were derived plotting the product formation rate as a function of the substrate concentration. The best fit of the experimental data provides a *K*_M_ value of 86.5±10.5 μm according to the Henry‐Michaelis–Menten equation (left). B) ^19^F NMR of **F‐Lac** incubated with Pmα23ST in presence of CMP‐Neu5Ac. The formation of **F‐sLac** (black arrows) can be followed by ^19^F NMR in real‐time. Product formation was confirmed by HPLC (Figure S9).

The high sensitivity of the ^19^F reporter to subtle modifications in its chemical environment offers a valuable tool for real‐time monitoring of enzymatic reactions. The possibility to place the ^19^F reporter on a carbohydrate unit in proximity to the functionalization site is crucial for detecting a chemical shift perturbation. We selected two enzymes (β‐galactosidase^[72]^ and Pmα23ST^[53]^) and we monitored their activity on a model substrate, **F‐Lac**. Glycosidic bond cleavage, mediated by β‐galactosidase, was followed by ^19^F NMR. Cleavage of the terminal β‐galactose induced a chemical shift perturbation and real‐time ^19^F NMR tracking allowed for derivation of the *K*_M_ of the enzymatic reaction (Figure 5 A). Next, glycosidic bond formation promoted by Pmα23ST^[53]^ was monitored in real‐time. *N*‐Acetyl‐neuraminic acid (Neu5Ac) is transferred from an activated cytidine monophosphate donor (CMP‐Neu5Ac) to the C‐3 OH of the terminal galactose unit of **F‐Lac** to yield **F‐sLac**. The electron‐withdrawing nature of Neu5Ac induced a chemical shift perturbation of 0.2 ppm on the ^19^F‐labeled acceptor, allowing to track in real‐time the enzymatic sialylation process (Figure 5 B). When the ^19^F reporter was positioned remotely to the reactive site of the acceptor (>3 sugar units away, **F‐nLac_4_**), no chemical shift perturbation was noticed, despite the success of the enzymatic transformation (Figure S10). Thus, in contrast to what is observed for protein binding, the position of the ^19^F reporter is key for monitoring enzymatic reactions.

## Conclusion

AGA enabled the fast assembly of ^19^F‐labeled Lewis type 2 antigens for the high‐throughput screening of protein binding. Mammalian and bacterial lectins as well as enzymes were analyzed. ^19^F NMR screening of F‐glycans permitted a quick qualitative evaluation as well as a reliable quantification of lectin binding (*K*_d_). The assay does not require labeled proteins or complex 2D NMR experiments. All NMR experiments can be performed in an extremely small scale (few nmol of glycan and protein per experiment). Enzymatic reactions, including sialylation, were monitored in real‐time, demonstrating that ^19^F‐labeled glycans hold a great potential as molecular probes to uncover enzymatic processes and for high‐throughput screening.^[27]^ Protocols for the selective ^19^F‐labeling of monosaccharides are available;[^73^, ^74^, ^75^] the implementation of these novel BBs in AGA will fuel the production of new classes of glycan probes. Given the high dispersion of ^19^F NMR signals, libraries of F‐glycans with diverse chemical shifts can be designed to increase the high throughput of this approach.^[76]^ The ability of ^19^F glycan probes to reveal binding or enzymatic transformation in solution and in real‐time could open the way to in cell NMR applications, often hampered by high background signals.[^14^, ^77^, ^78^] Overall, these probes are valuable tools for a better molecular understanding of the interactions of complex glycans with protein receptors.

## Conflict of interest

The authors declare no conflict of interest.

## Supporting information

As a service to our authors and readers, this journal provides supporting information supplied by the authors. Such materials are peer reviewed and may be re‐organized for online delivery, but are not copy‐edited or typeset. Technical support issues arising from supporting information (other than missing files) should be addressed to the authors.

Supplementary
