# Supplementary material for: Automated Glycan Assembly of 19F‐labeled Glycan Probes Enables High‐Throughput NMR Studies of Protein–Glycan Interactions
Source: Angew Chem Int Ed Engl. 2021 May 7;60(24):13302–9. doi: 10.1002/anie.202102690 (PMC8252726; doi:10.1002/anie.202102690)
Supplement: Supplementary file 1 — Supplementary [file ANIE-60-13302-s001.pdf]

Angewandte Chemie
Eine Zeitschrift der Gesellschaft Deutscher Chemiker

Supporting Information

Automated Glycan Assembly of ^{19}F-labeled Glycan Probes Enables High-Throughput NMR Studies of Protein–Glycan Interactions

Giulio Fittolani[+], Elena Shanina[+], Mónica Guberman, Peter H. Seeberger, Christoph Rademacher, and Martina Delbianco**

anie_202102690_sm_miscellaneous_information.pdf

Table of Contents

A. General materials and methods 2

B. Building blocks 3

C. Automated glycan assembly 4

General materials and method 4

Preparation of stock solutions 4

Modules for automated synthesis 4

Post-synthesizer manipulations (Post-AGA) 8

Oligosaccharides synthesis 10

Synthesis of **F-Lac** 11

Synthesis of **F-nLac$_4$** 16

Synthesis of **F-Le^x** 21

Synthesis of **F-H type 2** 26

Synthesis of **F-Le^y** 31

Synthesis of **CF_3-H type 2** 36

D. NMR studies of glycan-protein interactions 39

General materials and methods 39

E. References 58

A. General materials and methods

All chemicals used were reagent grade and used as supplied unless otherwise noted. The automated syntheses were performed on a home-built synthesizer developed at the Max Planck Institute of Colloids and Interfaces. Analytical thin-layer chromatography (TLC) was performed on Merck silica gel 60 F254 plates (0.25 mm). Compounds were visualized by UV irradiation or dipping the plate in a staining solution (sugar stain: 10% H_2SO_4 in EtOH; CAM: 48 g/L ammonium molybdate, 60 g/L ceric ammonium molybdate in 6% H_2SO_4 aqueous solution). Flash column chromatography was carried out by using forced flow of the indicated solvent on Fluka Kieselgel 60 M (0.04 – 0.063 mm). Analysis and purification by normal and reverse phase HPLC was performed by using an Agilent 1200 series. Products were lyophilized using a Christ Alpha 2-4 LD plus freeze dryer. ^{1}H, ^{13}C and HSQC NMR spectra were recorded on a Varian 400-MR (400 MHz), Varian 600-MR (600 MHz), or Bruker Biospin AVANCE700 (700 MHz) spectrometer. Spectra were recorded in $CDCl_3$ by using the solvent residual peak chemical shift as the internal standard ($CDCl_3$: 7.26 ppm ^{1}H, 77.0 ppm ^{13}C) or in D_2O using the solvent as the internal standard in ^{1}H NMR (D_2O: 4.79 ppm ^{1}H). High resolution mass spectra were obtained using a 6210 ESI-TOF mass spectrometer (Agilent) and a MALDI-TOF autoflex^{TM} (Bruker). MALDI and ESI mass spectra were run on IonSpec Ultima instruments. IR spectra were recorded on a Perkin-Elmer 1600 FTIR spectrometer. Optical rotations were measured by using a Perkin-Elmer 241 and Unipol L1000 polarimeter.

B. Building blocks

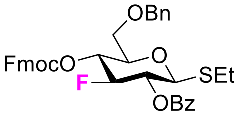

1

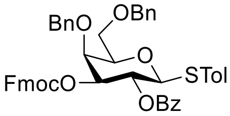

2

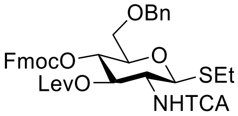

3

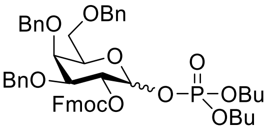

4

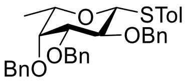

5

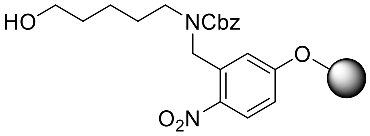

L1

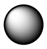 = Merrifield resin

Building blocks **1** and **4** were synthesized according to previous literature procedures.[1,2] Building blocks **2**, **3**, and **5** were purchased from GlycoUniverse (Germany, product codes Gal32.11140202, GlcN30.15131402, Fuc32.020202 respectively). Merrifield resin equipped with a photocleavable linker (**L1**, loading 0.30 mmol/g) was prepared according to previous literature.[3]

C. Automated glycan assembly

General materials and method

The automated syntheses were performed on a home-built synthesizer developed at the Max Planck Institute of Colloids and Interfaces. All solvents used were HPLC-grade. The solvents used for the building block, activator, TMSOTf and capping solutions were taken from an anhydrous solvent system (J.C. Meyer) and further dried with molecular sieves (4 Å) for moisture-sensitive solutions. The building blocks were co-evaporated three times with toluene and dried for 1 h on high vacuum before use. Oven-heated, argon-flushed flasks were used to prepare all moisture-sensitive solutions. Activator, capping, deprotection, acidic wash and building block solutions were freshly prepared and kept under argon during the automation run. All yields of products obtained by AGA were calculated on the basis of resin loading. Resin loading was determined following previously established procedures.[4]

Preparation of stock solutions

- **Building block solution**: Between 0.06 and 0.10 mmol of building block (depending on the BB, see Module C1 and C2) was dissolved in DCM (1 mL).
- **NIS/TfOH activator solution**: 1.35 g (6.0 mmol) of recrystallized NIS was dissolved in 40 mL of a 2:1 v/v mixture of anhydrous DCM and anhydrous dioxane. Then triflic acid (55 µL, 0.6 mmol) was added. The solution is kept at 0°C for the duration of the automation run.
- **Fmoc deprotection solution**: A solution of 20% piperidine in DMF (v/v) was prepared.
- **Lev deprotection solution**: Hydrazine acetate (550 mg, 5.97 mmol) was dissolved in pyridine/AcOH/H2O (40mL, v/v, 32:8:2) and sonicated for 10 min.
- **TMSOTf solution**: TMSOTf (0.45 mL, 2.49 mmol) was added to DCM (40 mL).
- **Capping solution**: A solution of 10% acetic anhydride and 2% methanesulfunic acid in DCM (v/v) was prepared.

Modules for automated synthesis

Module A: Resin preparation for synthesis (20 min)

All automated syntheses were performed on 0.0135 mmol scale. Resin (**L1**, 45 mg) was placed in the reaction vessel and swollen in DCM for 20 min at room temperature prior to synthesis. During this time, all reagent lines needed for the synthesis were washed and primed. After the swelling, the resin was washed with DMF, THF, and DCM (three times each with 2 mL for 25 s).

Module B: Acidic wash with TMSOTf solution (20 min)

The resin was swollen in 2 mL DCM and the temperature of the reaction vessel was adjusted to -20 °C. Upon reaching the low temperature, TMSOTf solution (1 mL) was added drop wise to the reaction vessel. After bubbling for 3 min, the acidic solution was drained and the resin was washed with 2 mL DCM for 25 s.

Action	Cycles	Solution	Amount	T (°C)	Incubation time
Cooling	-	-	-	-20	(15 min)*
Deliver	1	DCM	2 mL	-20	-
Deliver	1	TMSOTf solution	1 mL	-20	3 min
Wash	1	DCM	2 mL	-20	25 sec

*Time required to reach the desired temperature.

Module C1: Thioglycoside glycosylation (35 min-55 min)

The building block solution (0.10 mmol of BB in 1 mL of DCM per glycosylation) was delivered to the reaction vessel. After the set temperature was reached, the reaction was started by dropwise addition of the NIS/TfOH activator solution (1.0 mL, excess). The glycosylation conditions (T_1, T_2, t_1, and t_2) are building block dependent and are reported in a table below. After completion of the reaction, the solution was drained and the resin was washed with DCM, DCM:dioxane (1:2, 3 mL for 20 s) and DCM (two times, each with 2 mL for 25 s). The temperature of the reaction vessel was increased to 25 °C for the next module.

Action	Cycles	Solution	Amount	T (°C)	Incubation time
Cooling	-	-	-	T_1	-
Deliver	1	BB solution	1 mL	T_1	-
Deliver	1	NIS/TfOH activator solution	1 mL	T_1	-
Reaction time (BB dependent)	1			T_1 to T_2	t_1 t_2
Wash	1	DCM	2 mL	T_2	5 sec
Wash	1	DCM : Dioxane (1:2)	2 mL	T_2	20 sec
Heating	-	-	-	25	-
Wash	2	DCM	2 mL	> 0	25 sec

The AGA glycosylation conditions employed for thioglycoside BBs were previously reported.[1,2]

BB	Equiv.	t_1 (min)	T_1 (°C)	t_2 (min)	T_2 (°C)
**1**	8	5	-20	20	0
**2**	8	5	-20	20	0
**3**	8	5	-20	40	0
**5**	8	5	-40	20	-20

Module C2: Glycosyl phosphate glycosylation (45 min)

The building block solution (0.06 mmol of BB in 1 mL of DCM per glycosylation) was delivered to the reaction vessel. After the set temperature was reached, the reaction was started by drop wise addition of the TMSOTf solution (1.0 mL, stoichiometric). After completion of the reaction, the solution was drained and the resin washed with DCM (six times, each with 2 mL for 25 s). The temperature of the reaction vessel was increased to 25 °C for the next module.

Action	Cycles	Solution	Amount	T (°C)	Incubation time
Cooling	-	-	-	-35	-
Deliver	1	BB solution	1 mL	-35	-
Deliver	1	TMSOTf solution	1 mL	-35	-
Reaction time (BB dependent)	1			-35 to -15	5 min 30 min
Wash	1	DCM	2 mL	-15	5 sec
Heating	-	-	-	25	-
Wash	6	DCM	2 mL	> 0	25 sec

The AGA glycosylation conditions employed for the glycosyl phosphate BB were previously reported.[2]

BB	Equiv.	t_1 (min)	T_1 (°C)	t_2 (min)	T_2 (°C)
**4**	5	5	-35	30	-15

Module D: Capping (30 min)

The resin was washed with DMF (two times with 2 mL for 25 s) and the temperature of the reaction vessel was adjusted to 25 °C. 2 mL of Pyridine solution (10% in DMF) was delivered into the reaction vessel. After 1 min, the reaction solution was drained and the resin washed with DCM (three times with 3 mL for 25 s). 4 mL of capping solution was delivered into the

reaction vessel. After 20 min, the reaction solution was drained and the resin washed with DCM (three times with 3 mL for 25 s).

Action	Cycles	Solution	Amount	T (°C)	Incubation time
Heating	-	-	-	25	(5 min)*
Wash	2	DMF	2 mL	25	25 sec
Deliver	1	10% Pyridine in DMF	2 mL	25	1 min
Wash	3	DCM	2 mL	25	25 sec
Deliver	1	Capping Solution	4 mL	25	20 min
Wash	3	DCM	2 mL	25	25 sec

*Time required to reach the desired temperature.

Module E1: Fmoc deprotection (9 min)

The resin was washed with DMF (three times with 2 mL for 25 s) and the temperature of the reaction vessel was adjusted to 25 °C. 2 mL of Fmoc deprotection solution was delivered to the reaction vessel and kept under Ar bubbling. After 5 min, the reaction solution was drained and the resin washed with DMF (three times with 3 mL for 25 s) and DCM (five times each with 2 mL for 25 s). The temperature of the reaction vessel was decreased to -20 °C for the next module.

Action	Cycles	Solution	Amount	T (°C)	Incubation time
Wash	3	DMF	2 mL	25	25 sec
Deliver	1	Fmoc depr. solution	2 mL	25	5 min
Wash	1	DMF	2 mL		
Cooling	-	-	-	-20	-
Wash	3	DMF	2 mL	< 25	25 sec
Wash	5	DCM	2 mL	< 25	25 sec

Module E2: Lev deprotection (65 min)

The resin was washed with DCM (three times with 2 mL for 25 s). DCM (1.3 mL) was delivered to the reaction vessel and the temperature of the reaction vessel was adjusted to 25 °C. 2 mL of Lev deprotection solution was delivered to the reaction vessel that was kept under pulsed Ar bubbling for 30 min. This procedure was repeated twice. The reaction solution was drained and the resin washed with DMF (three times with 3 mL for 25 s) and DCM (five times each with 2 mL for 25 s).

Action	Cycles	Solution	Amount	T (°C)	Incubation

					time
Wash	3	DMF	2 mL	25	25 sec
Deliver	2	Lev depr. solution	2 mL	25	30 min
Wash	1	DMF	2 mL		
Cooling	-	-	-	-20	-
Wash	3	DMF	2 mL	< 25	25 sec
Wash	5	DCM	2 mL	< 25	25 sec

Note:

With the current setup the automated synthesizer has four BB lines. Therefore, for AGA syntheses requiring the use of five BBs (*i.e.* AGA of **F-Le^x**, **F-H type 2**, and **F-Le^y**) a first cycle with BB **1** was performed and, upon completion, BB **1** was replaced by BB **5** solution to continue the AGA.

Post-synthesizer manipulations (Post-AGA)

Module F: On-resin methanolysis

The resin was suspended THF (4 mL). 0.4 mL of NaOMe in MeOH (0.5 M) was added and the suspension was gently shaken at room temperature. After micro-cleavage (see Module G1) indicated the complete removal of benzoyl groups, the resin was repeatedly washed with MeOH (2mL x 3) and DCM (2mL x 3).

Module G: Cleavage from solid support

The oligosaccharides were cleaved from the solid support using a continuous-flow photoreactor as described previously.[5]

Module G1: Micro-cleavage from solid support

Trace amount of resin (around 20 beads) was dispersed in DCM (0.1 mL) and irradiated with a UV lamp (6 watt, 356 nm) for 10 minutes. ACN (10 µL) was then added to the resin and the resulting solution analyzed by MALDI.

Module H1: Hydrogenolysis

The crude compound obtained from *Module G* was dissolved in 2 mL of EA:*t*BuOH:H_2O (2:1:1). 100% by weight Pd-C (10%) was added and the reaction was stirred in H_2 bomb with 60 psi pressure. The reaction progress was monitored to avoid undesired side products formation.

Upon completion, the reaction was filtered and washed with EA, *t*BuOH and H_2O. The filtrates were concentrated *in vacuo.*

Module H2: Hydrogenolysis at ambient pressure

The crude compound obtained from *Module G* was dissolved in 2 mL of EA:*t*BuOH:H_2O (2:1:1). 100% by weight Pd-C (10%) was added and the reaction was stirred in a flask equipped with a H_2 balloon. The reaction progress was monitored to avoid undesired side products formation. Upon completion, the reaction was filtered and washed with EA, *t*BuOH and H_2O. The filtrates were concentrated *in vacuo.*

Module I: Purification

The purification of the crudes was conducted using a C_{18} silica column or reverse phase HPLC (Agilent 1200 Series, Method B and Method C). The pure compound was analyzed using analytical HPLC (Agilent 1200 Series, Method A).

- **Method A:** (Hypercarb column, 150 x 4.6 mm, 3 μm) flow rate of 0.7 mL/min with H_2O (0.1% formic acid) as eluents [isocratic (5 min), linear gradient to 30% ACN (30 min), linear gradient to 100% ACN (5 min)].
- **Method B:** (Hypercarb column, 150 x 10 mm, 5 μm), flow rate of 3 mL /min with H_2O (0.1% formic acid) as eluents [isocratic (5 min), linear gradient to 30% ACN (30 min), linear gradient to 100% ACN (5 min)].
- **Method C:** (Manual reverse phase C_{18} silica gel column chromatography): H_2O (0.1% formic acid, 10 mL), 3% MeOH (10 mL), 6% MeOH (10 mL), 9% MeOH (10 mL), 15% MeOH (10 mL).

Following final purification, all deprotected products were lyophilized on a Christ Alpha 2-4 LD plus freeze dryer prior to characterization.

Oligosaccharides synthesis

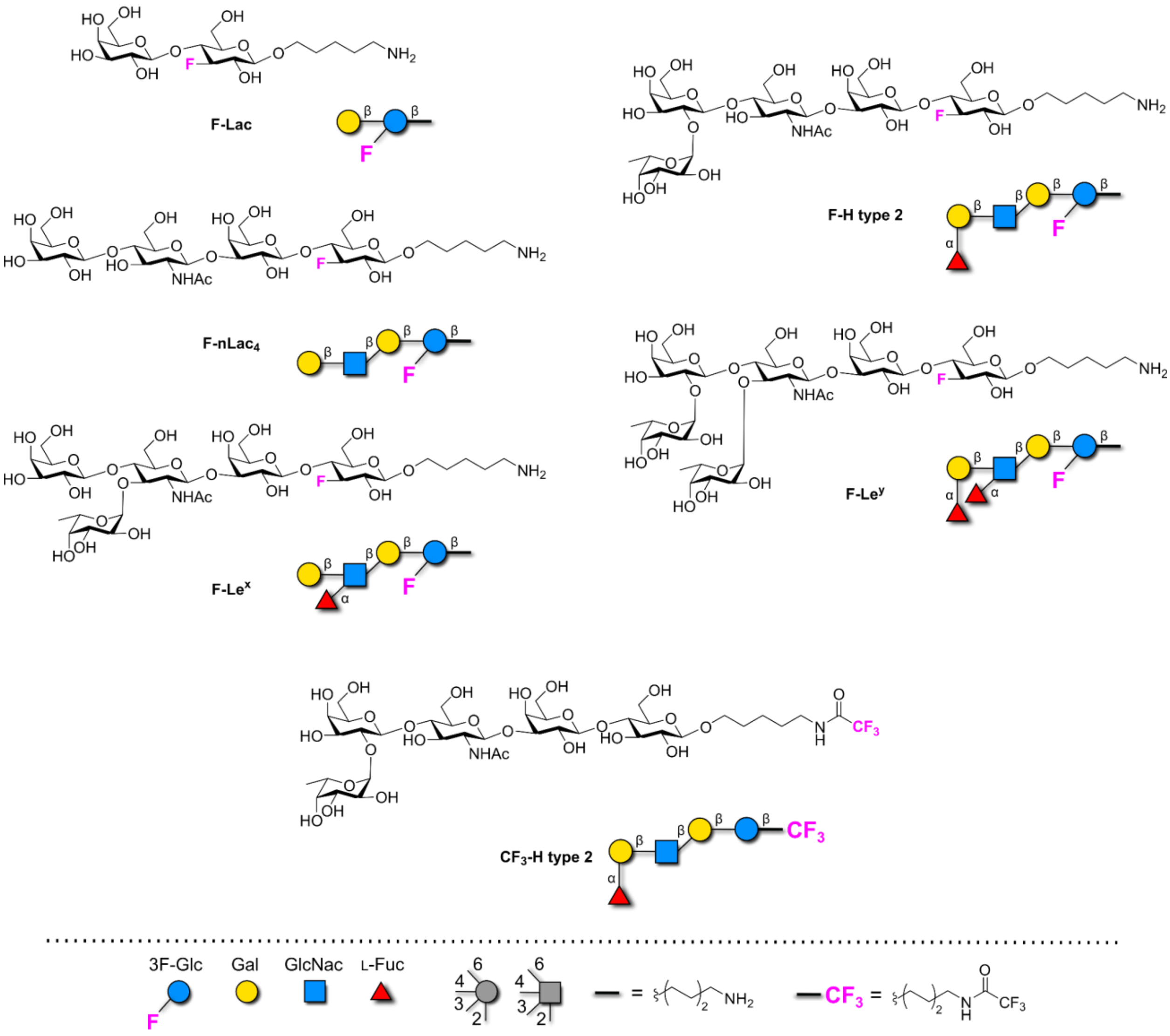

Figure S1 Collection of ^{19}F labelled Lewis type-II antigens synthesized by AGA.

Synthesis of **F-Lac**

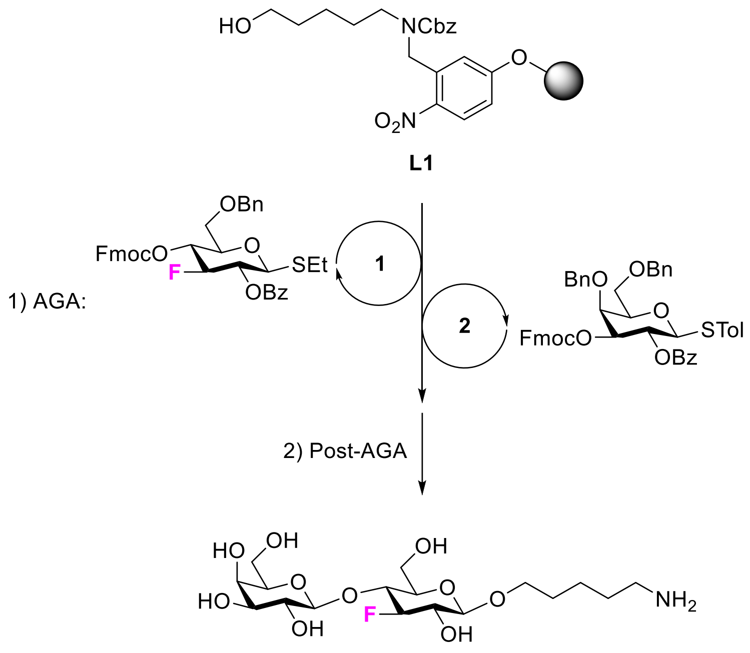

F-Lac

Step	BB	Modules	Notes
AGA		**A**	**L1** swelling
	**1**	**B**, **C1**, **D**, **E1**	**C1**: (**1**, -20°C for 5 min, 0°C for 20 min)
	**2**	**B**, **C1**, **D**, **E1**	**C1**: (**2**, -20°C for 5 min, 0°C for 20 min)
Post-AGA		**F**, **G**, **H2**, **I**	**F**: (16 h)<br>**H2**: (3 h)<br>**I**: (Method B, t_R = 19.6 min)

Automated synthesis, global deprotection, and purification afforded **F-Lac** as a white solid (1.17 mg, 20% overall yield).

Analytical data for **F-Lac**:

^{1}H NMR (600 MHz, Deuterium Oxide) δ 4.67 – 4.55 (m, 1H, **H-1 Glc**, H-3 Glc), 4.54 (d, *J* = 8.1 Hz, 1H, **H-1 Gal**), 4.50 (d, *J* = 7.9 Hz, 1H), 4.05 – 3.92 (m, 4H), 3.87 (dd, *J* = 12.3, 5.1 Hz, 1H), 3.81 (dd, *J* = 11.8, 7.7 Hz, 1H), 3.78 – 3.65 (m, 4H), 3.64 – 3.53 (m, 3H), 3.01 (t, *J* = 7.5 Hz, 2H, CH_2-NH_3^+ linker), 1.70 (h, *J* = 7.4, 6.8 Hz, 4H, 2x CH_2 linker), 1.47 (p, *J* = 7.7 Hz, 2H, CH_2 linker). ^{13}C NMR (151 MHz, Deuterium Oxide) δ 102.81 (s, **C-1 Gal**), 101.11 (d, *J* = 12.1 Hz, **C-1 Glc**), 94.77 (d, *J* = 183.6 Hz, C-3 Glc), 75.46 (d, *J* = 16.9 Hz), 75.18, 73.80 (d, *J* = 8.1 Hz), 72.57, 71.72 (d, *J* = 18.1 Hz), 70.92, 70.17 (s, CH_2-O linker), 68.43, 60.79, 59.77, 39.31 (s, CH_2-

NH_3^+ linker), 28.07 (s, CH_2 linker), 26.49 (s, CH_2 linker), 22.01 (s, CH_2 linker). ^{19}F NMR (564 MHz, Deuterium Oxide) δ -192.23 (dt, J = 52.1, 14.0 Hz). (ESI-HRMS) m/z 430.208 $[M+H]^+$ ($C_{17}H_{33}FNO_{10}$ requires 430.208).

RP-HPLC of F-Lac (ELSD trace, Method A, t_R = 21.4 min)

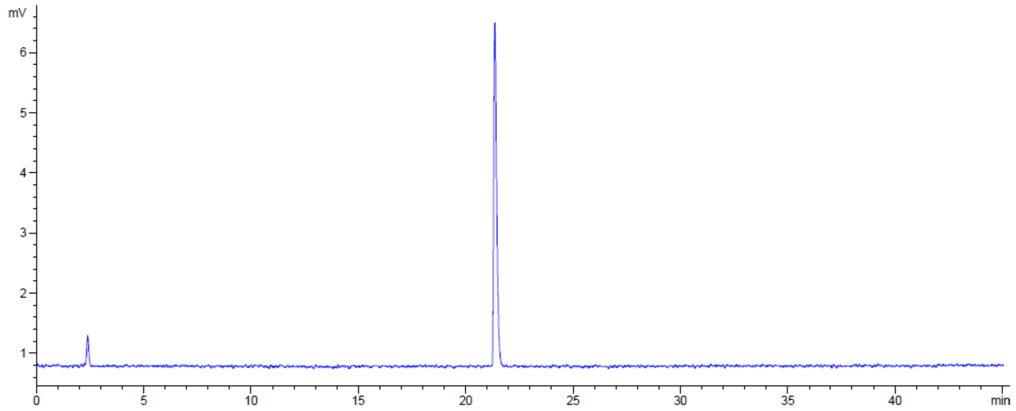

^{1}H NMR of F-Lac (600 MHz, D_2O)

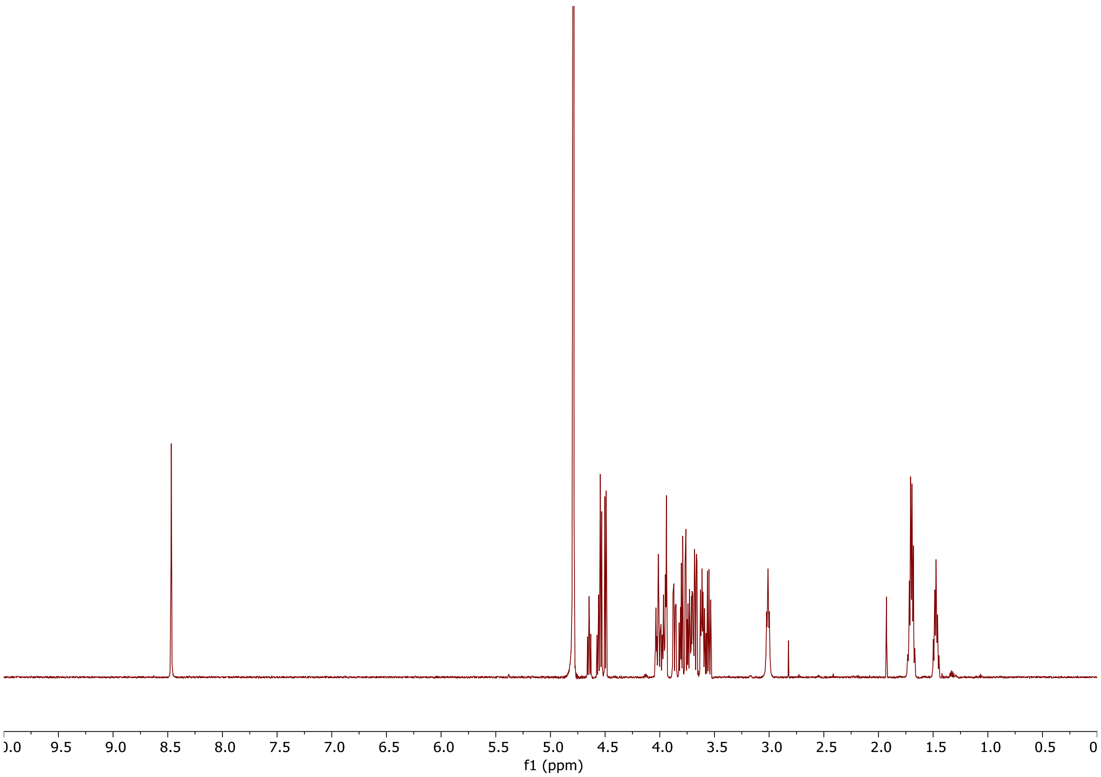

^{13}C NMR of F-Lac (151 MHz, D_2O)

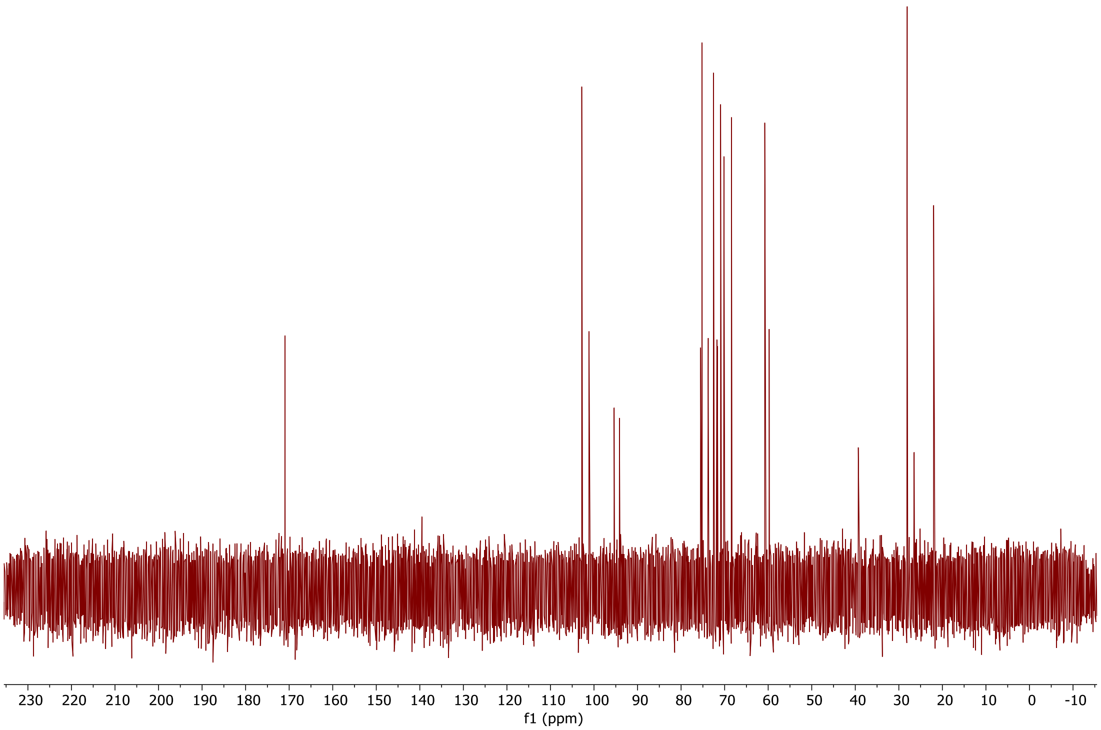

^{19}F NMR of F-Lac (564 MHz, D_2O)

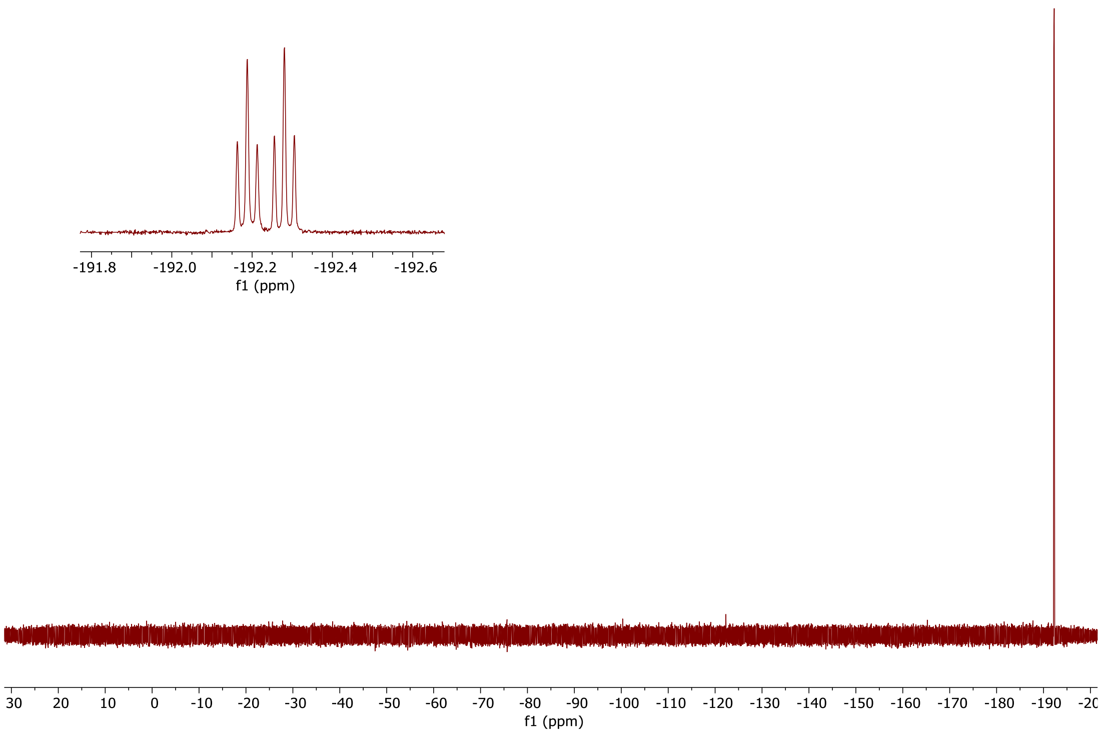

HSQC NMR of F-Lac (D_2O)

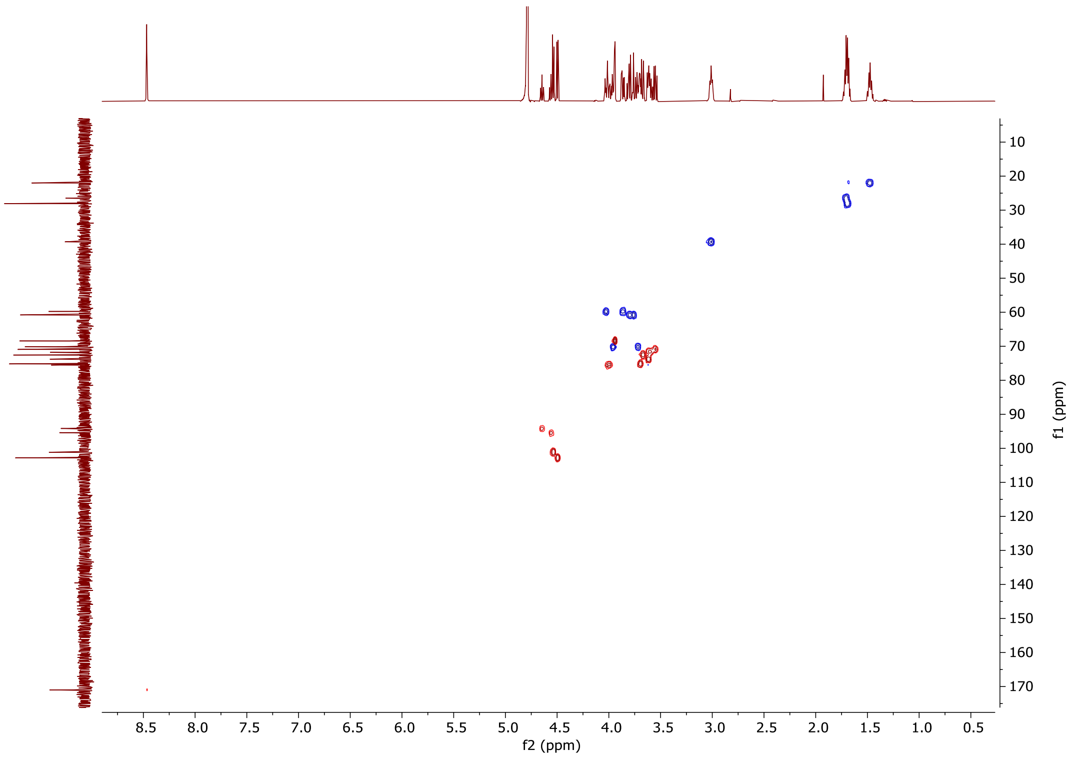

COSY NMR of F-Lac (D_2O)

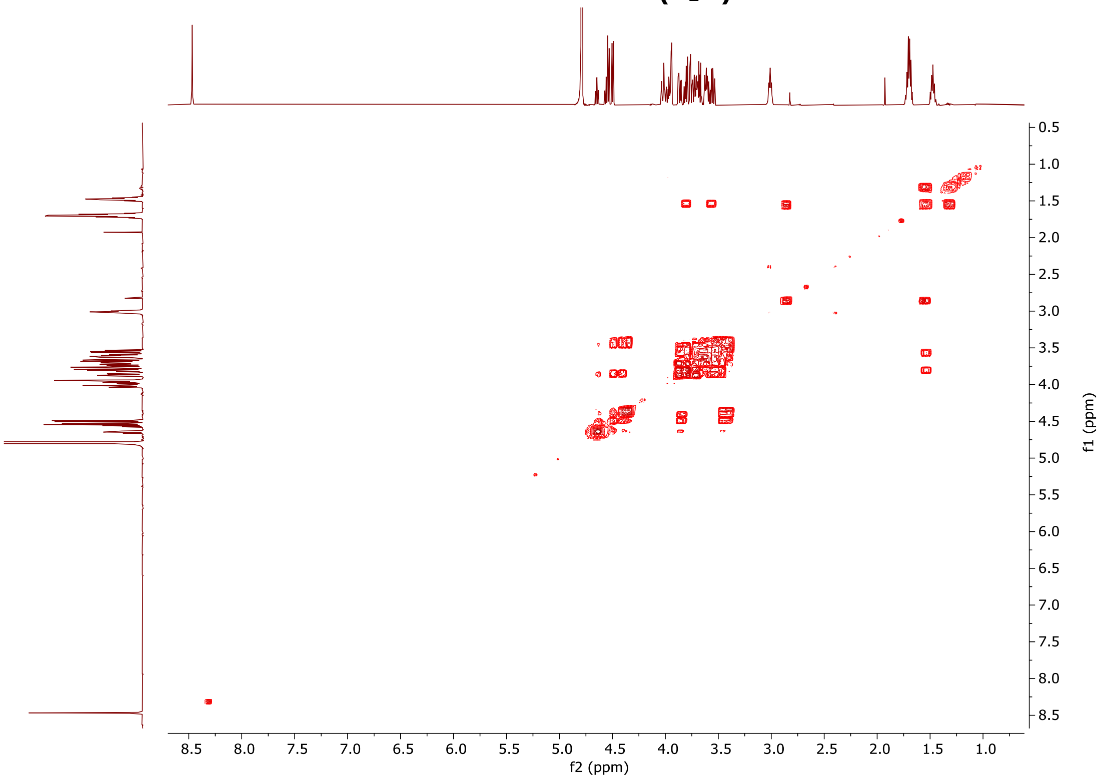

Synthesis of **F-nLac$_4$**

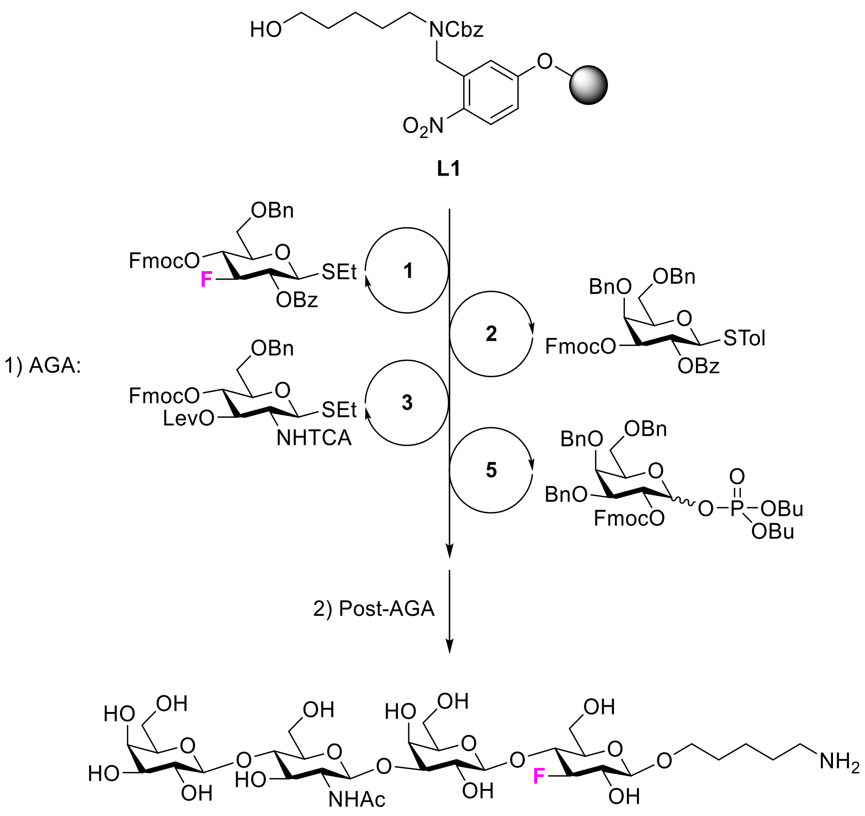

F-nLac$_4$

Step	BB	Modules	Notes
AGA		**A**	**L1** swelling
	**1**	**B**, **C1**, **D**, **E1**	**C1**: (**1**, -20°C for 5 min, 0°C for 20 min)
	**2**	**B**, **C1**, **D**, **E1**	**C1**: (**2**, -20°C for 5 min, 0°C for 20 min)
	**3**	**B**, **C1**, **D**, **E1**	**C1**: (**3**, -20°C for 5 min, 0°C for 40 min)
	**4**	**B**, **C2**, **D**, **E1**, **E2**	**C2**: (**4**, -35°C for 5 min, -15°C for 30 min)
Post-AGA		**F**, **G**, **H1**, **I**	**F:** (3 d)<br>**H1**: (3 d)<br>**I**: (Method C)

Automated synthesis, global deprotection, and purification afforded **F-nLac$_4$** as white solid (1.64 mg, 16% overall yield).

Analytical data for **F-nLac$_4$**:

^{1}H NMR (700 MHz, Deuterium oxide) 4.71 (d, J = 8.4 Hz, 1H, **H-1**), 4.59 (dt, J = 52.0, 7.8 Hz, 1H, H-3 Glc), 4.53 (d, J = 8.0 Hz, 1H, **H-1**), 4.48 (t, J = 8.8 Hz, 2H, 2x **H-1**), 4.16 (s, 1H), 4.07 – 3.89 (m, 5H), 3.89 – 3.65 (m, 14H), 3.64 – 3.50 (m, 5H), 3.00 (t, J = 7.7 Hz, 2H, CH_2-NH_3^+ linker), 2.04 (s, 3H, CH_3 NHAc), 1.73 – 1.64 (m, 4H, 2x CH_2 linker), 1.53 – 1.43 (m, 2H, CH_2 linker). ^{13}C NMR (151 MHz, Deuterium oxide) δ 174.84, 102.83 (s, **C-1**), 102.80 (s, **C-1**), 102.66 (s, **C-1**), 101.13 (s, **C-1**) 94.72 (d, J = 184.3 Hz, C-3 Glc), 82.13, 78.14, 75.57, 75.29, 74.72, 74.50, 73.81, 72.44, 72.12, 71.66, 70.90, 70.15 (s, CH_2-O linker), 69.92, 68.48, 68.18, 60.96, 60.72, 59.78, 55.14, 39.28 (s, CH_2 linker), 28.06 (s, CH_2 linker), 26.33 (s, CH_2 linker), 22.11 (s, CH_3 NHAc), 21.99 (s, CH_2 linker). ^{19}F NMR (564 MHz, Deuterium oxide) δ -192.16 (dt, J = 52.1, 14.0 Hz). (ESI-HRMS) m/z 795.344 $[M+H]^+$ ($C_{31}H_{56}FN_2O_{20}$ requires 795.341).

RP-HPLC of F-nLac$_4$ (ELSD trace, Method A, t_R = 28.1 min)

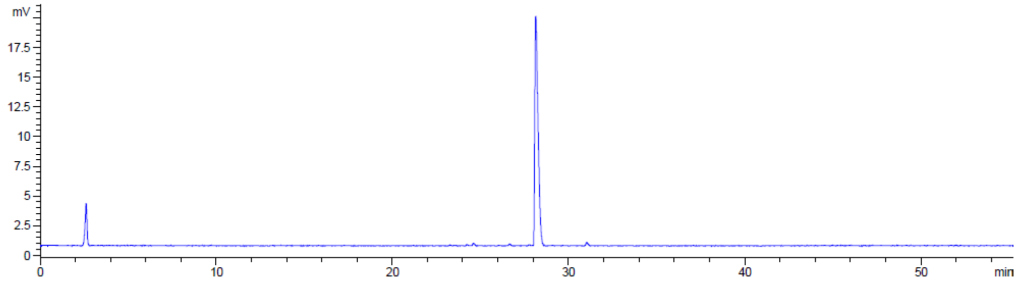

^{1}H NMR of F-nLac$_4$ (700 MHz, D_2O)

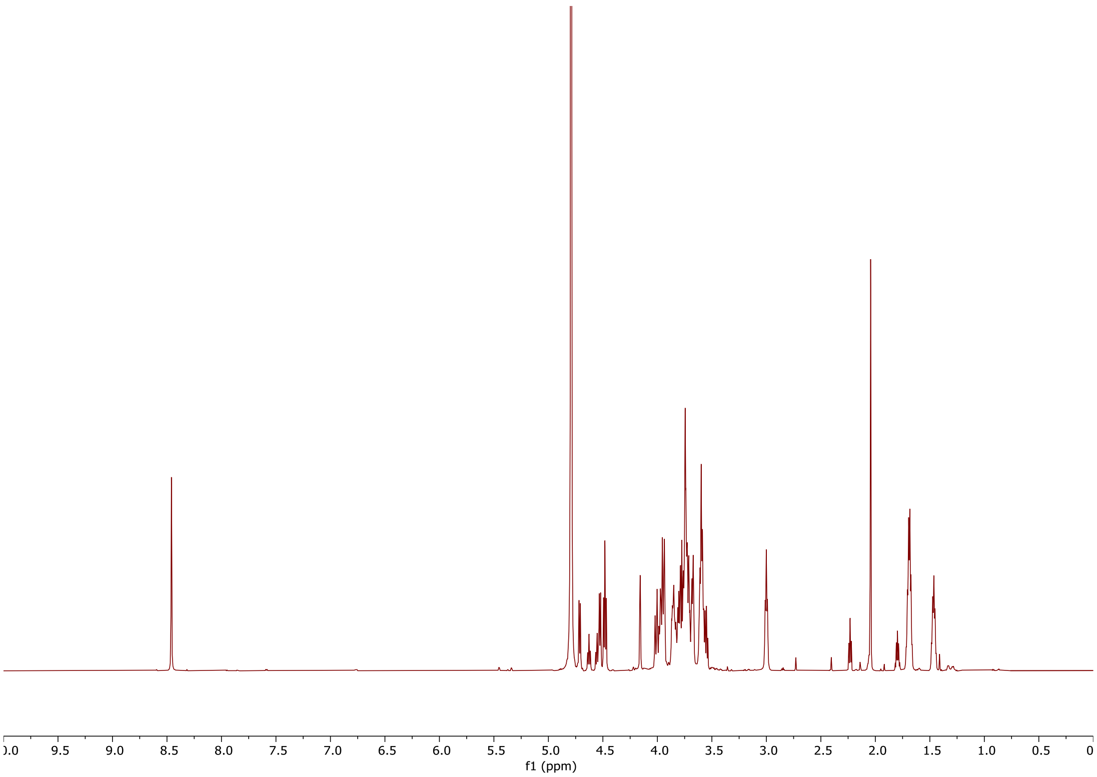

^{13}C NMR of F-nLac$_4$ (151 MHz, D_2O)

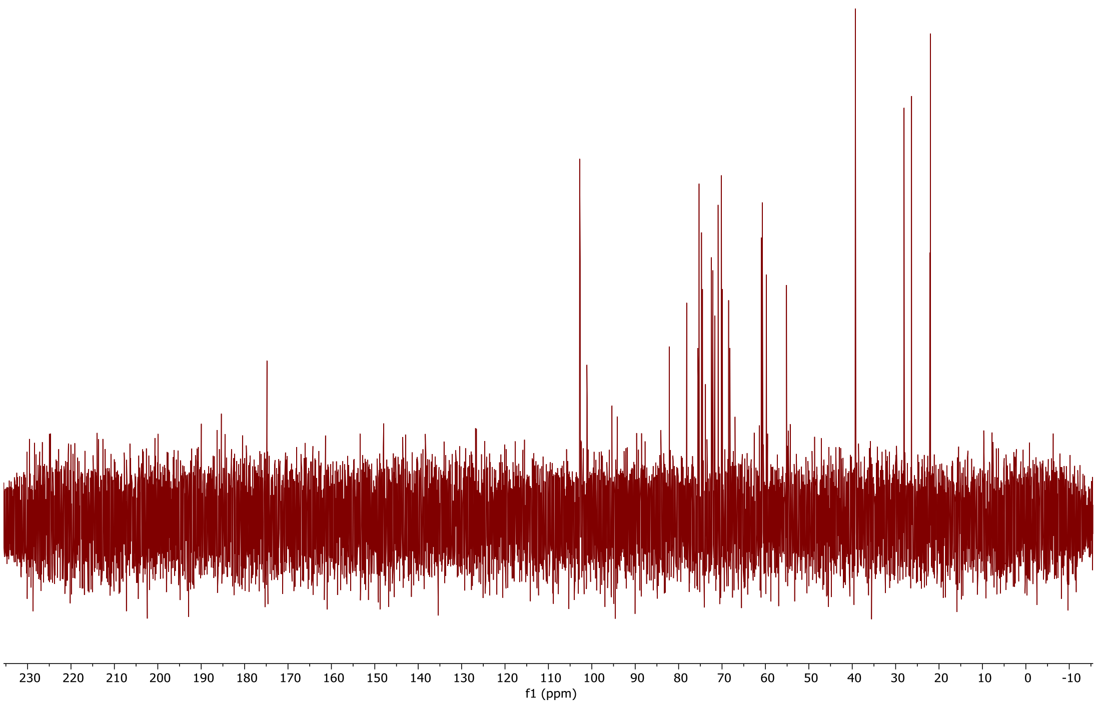

^{19}F NMR of F-nLac$_4$ (376 MHz, D_2O)

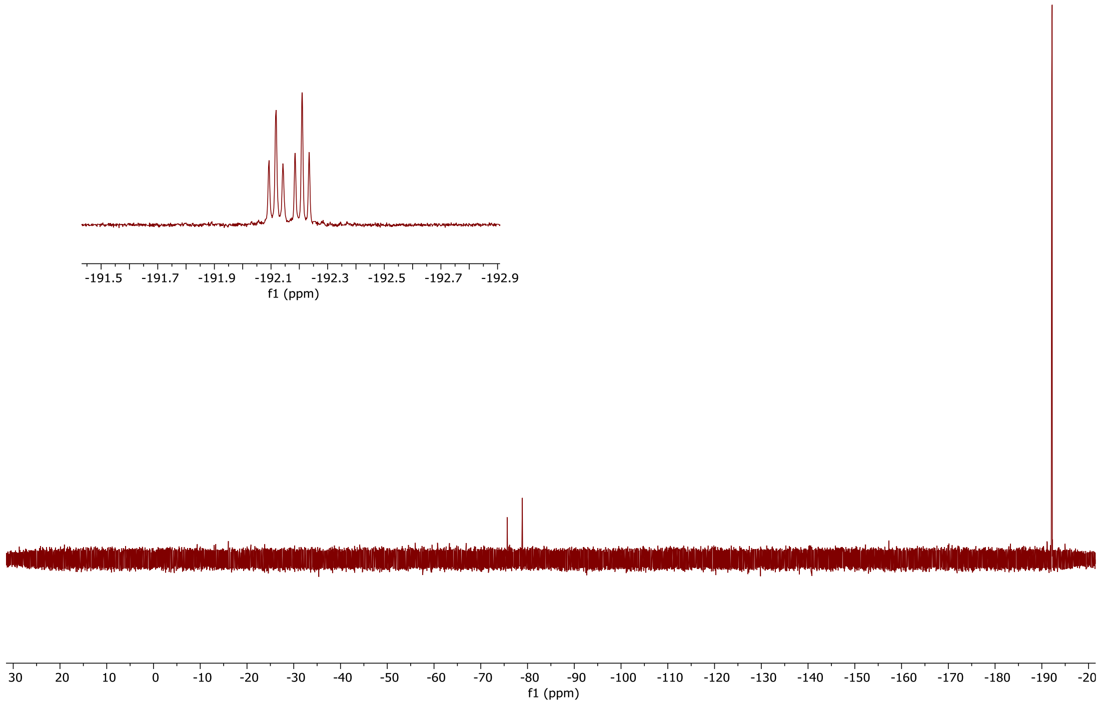

HSQC NMR of F-nLac$_4$ (D_2O)

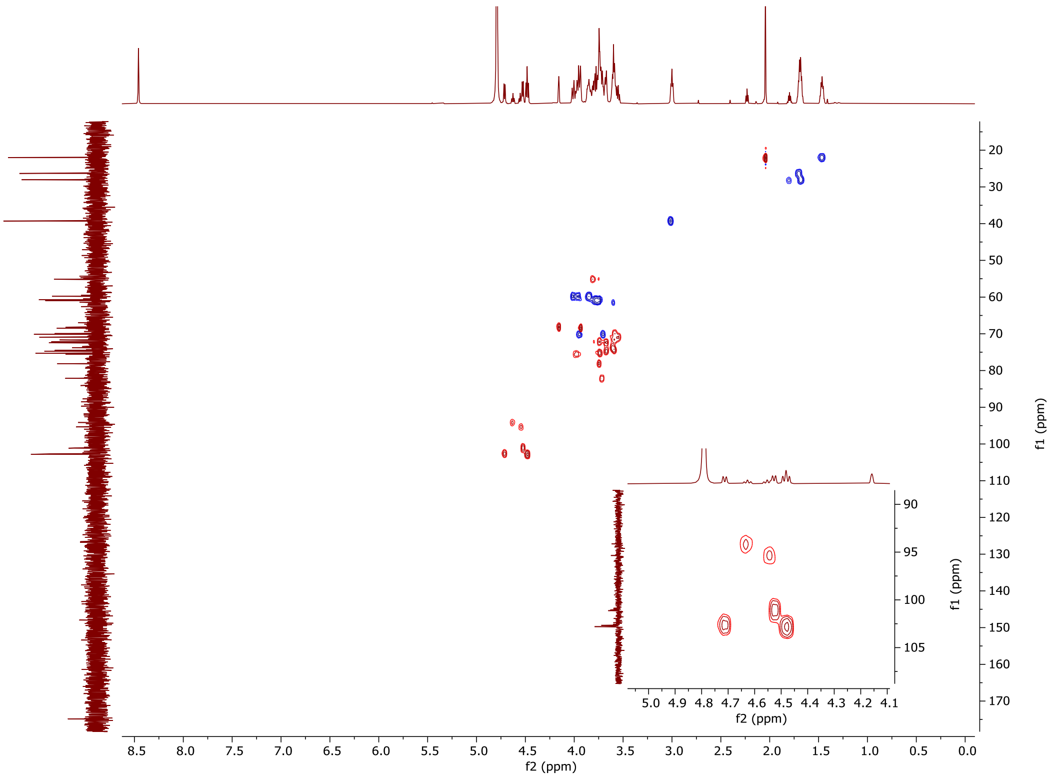

COSY NMR of F-nLac$_4$ (D_2O)

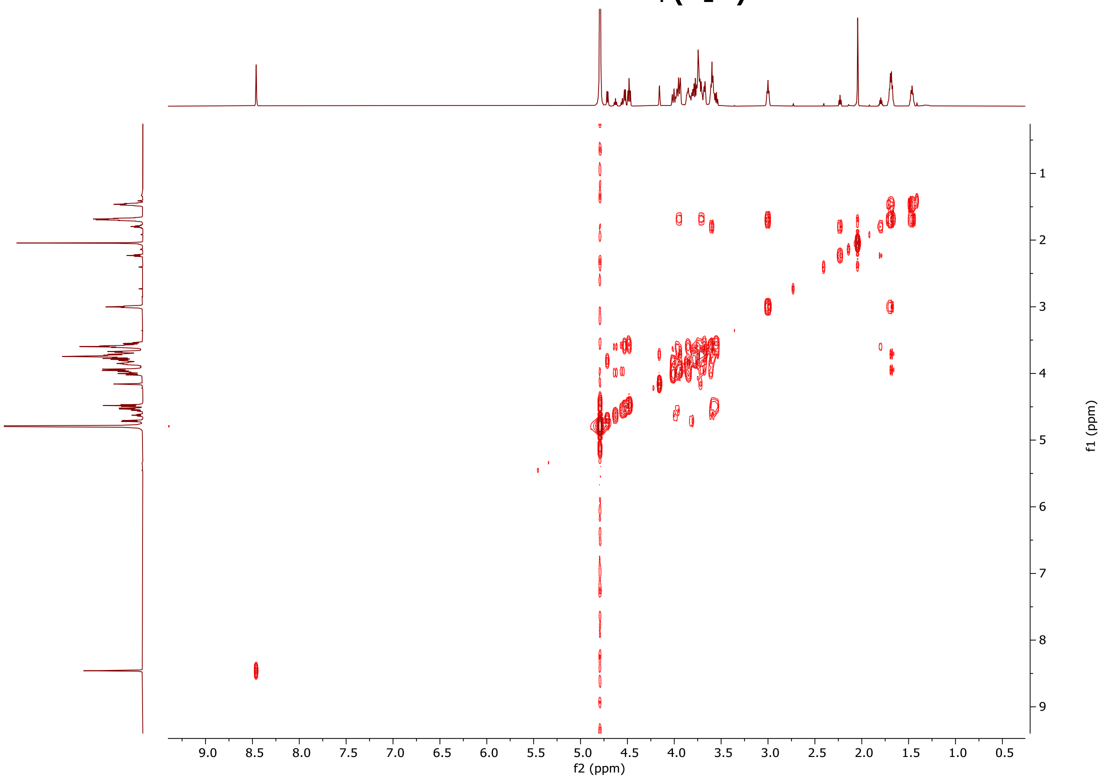

Synthesis of **F-Le^x**

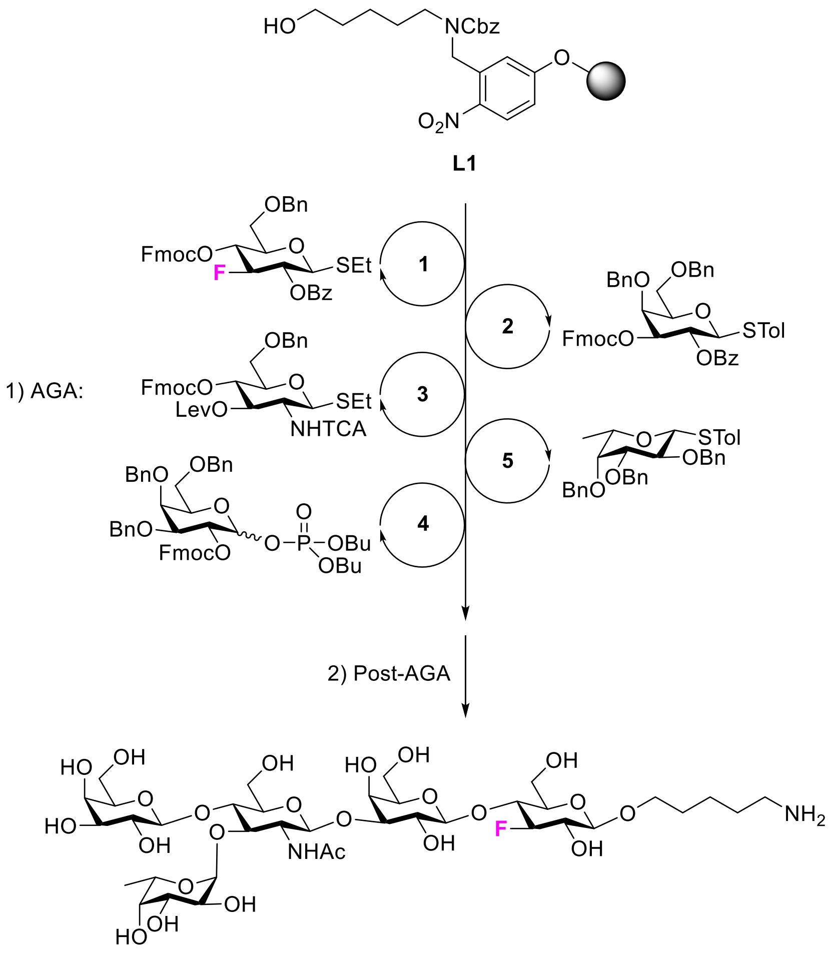

F-Le^x

Step	BB	Modules	Notes
AGA		**A**	**L1** swelling
	**1**	**B**, **C1**, **D**, **E1**	**C1**: (**1**, -20°C for 5 min, 0°C for 20 min)
	**2**	**B**, **C1**, **D**, **E1**	**C1**: (**2**, -20°C for 5 min, 0°C for 20 min)
	**3**	**B**, **C1**, **D**, **E2**	**C1**: (**3**, -20°C for 5 min, 0°C for 40 min)
	**5**	**B**, **C1**, **D**, **E1**	**C1**: (**5**, -40°C for 5 min, -20°C for 20 min)
	**4**	**B**, **C2**, **D**, **E1**	**C2**: (**4**, -35°C for 5 min, -15°C for 30 min)
Post-AGA		**F**, **G**, **H1**, **I**	**F:** (3 d)<br>**H1**: (3 d)<br>**I**: (Method C)

Automated synthesis, global deprotection, and purification afforded **F-Le^x** as a white solid (1.1 mg, 8% overall yield).

Analytical data for **F-Le^x**:

^{1}H NMR (700 MHz, Deuterium Oxide) δ 5.14 (s, 1H, **H-1 Fuc α-1,3**), 4.89 – 4.83 (m, 1H, H-5 Fuc α-1,3), 4.72 (d, *J* = 8.4 Hz, 1H, **H-1**), 4.67 – 4.51 (m, 2H, H-3 Glc, **H-1**), 4.50 – 4.44 (m, 2H, 2x **H-1**), 4.21 – 4.13 (m, 1H), 4.06 – 3.82 (m, 11H), 3.82 – 3.64 (m, 10H), 3.59 (ddt, *J* = 17.3, 10.2, 3.8 Hz, 5H), 3.51 (t, *J* = 8.9 Hz, 1H), 3.00 (t, *J* = 7.6 Hz, 2H, CH_2-NH_3^+ linker), 2.03 (s, 3H, CH_3 NHAc), 1.68 (p, *J* = 7.8, 7.1 Hz, 4H, 2x CH_2 linker), 1.46 (p, *J* = 7.5 Hz, 2H, CH_2 linker), 1.18 (d, *J* = 6.7 Hz, 3H, CH_3 Fuc). ^{13}C NMR (151 MHz, Deuterium oxide) δ 170.97 (s, *C*=O NHAc), 102.82 (s, **C-1**), 102.46 (s, **C-1**), 101.71 (s, **C-1**), 101.13 (s, **C-1**), 98.54 (s, **C-1 Fuc α-1,3**), 94.73 (d, *J* = 178.3 Hz, C-3 Glc), 82.16, 75.07, 74.85, 74.71, 73.81, 73.01, 72.42, 71.85, 70.99, 70.15 (s, CH_2-O linker), 69.92, 69.14, 68.29, 68.16, 67.65, 66.63 (s, C-5 Fuc α-1,3), 61.44, 60.73, 59.58, 39.29 (s, CH_2-NH_3^+ linker), 28.06 (s, CH_2 linker), 26.35 (s, CH_2 linker), 22.19 (s, CH_3 NHAc), 21.99 (s, CH_2 linker), 15.24 (s, CH_3 Fuc). ^{19}F NMR (564 MHz, Deuterium oxide) δ -192.15 (dt, *J* = 52.2, 14.0 Hz). (ESI-HRMS) m/z 941.403 $[M+H]^+$ ($C_{37}H_{66}FN_2O_{24}$ requires 941.398).

RP-HPLC of F-Le^x (ELSD trace, Method A, t_R = 24.3 min)

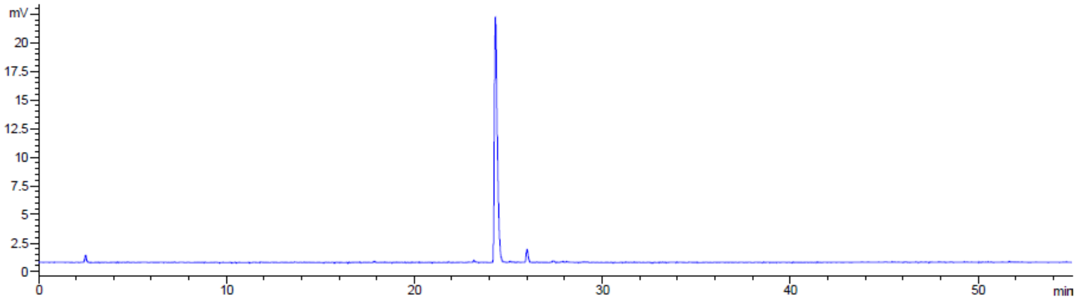

^{1}H NMR of F-Le^{x} (700 MHz, D_2O)

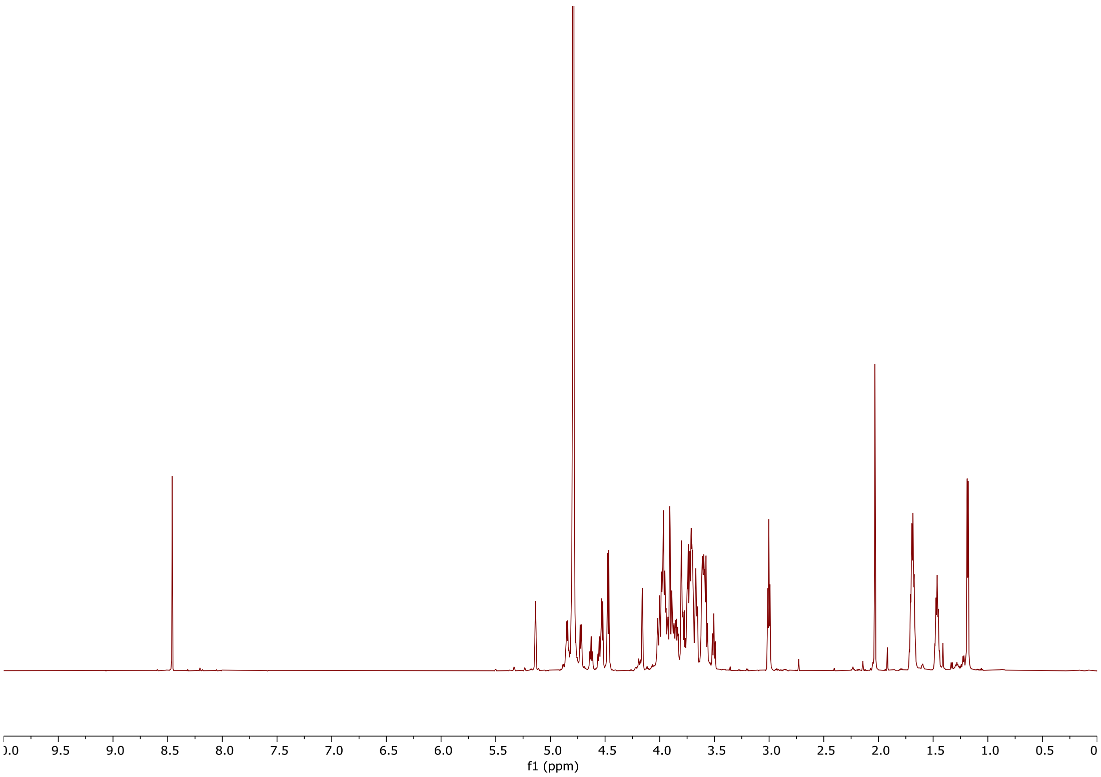

^{13}C NMR of F-Le^{x} (151 MHz, D_2O)

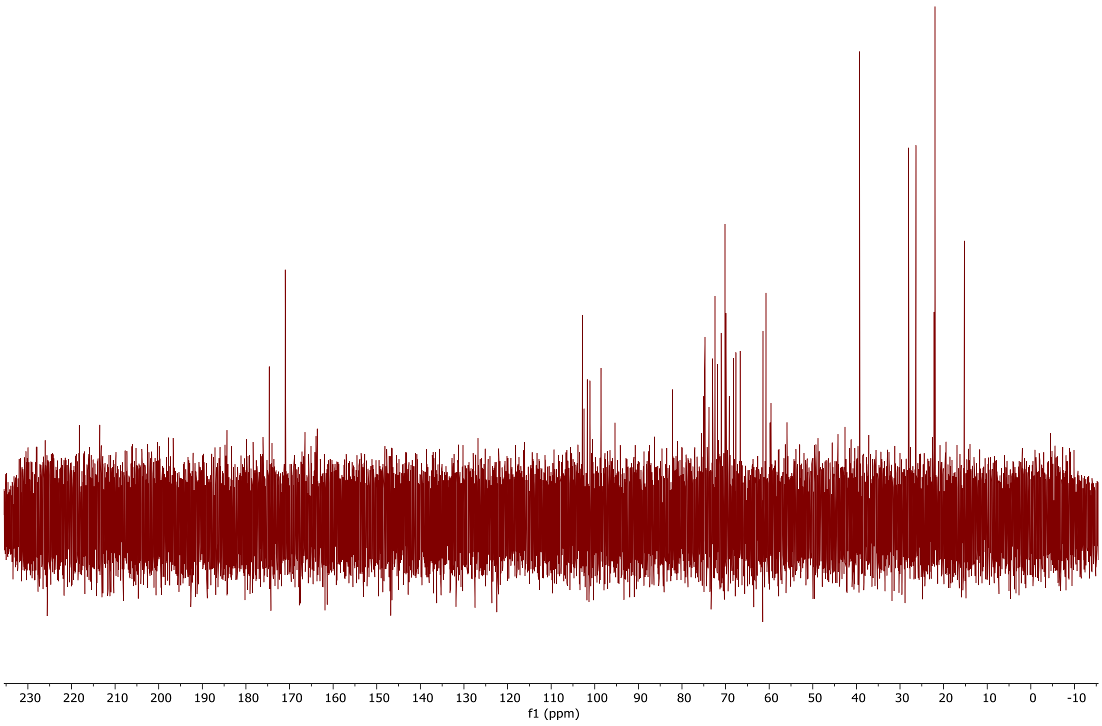

^{19}F NMR of F-Le^x (376 MHz, D_2O)

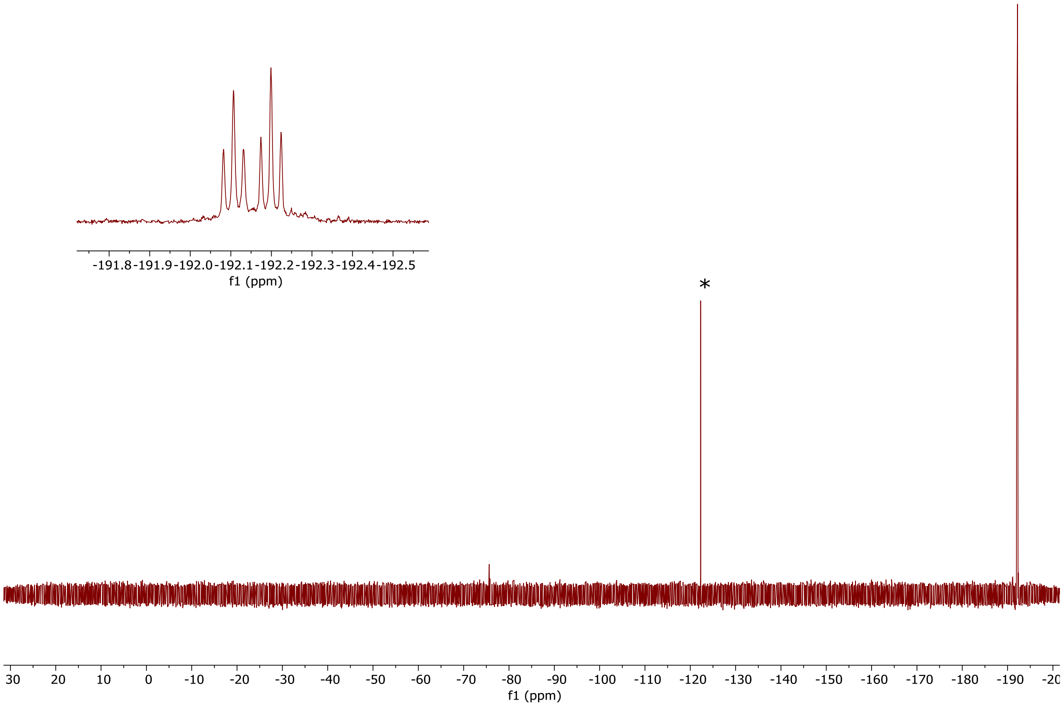

* The peak at -122.29 ppm is a fluoride impurity

HSQC NMR of F-Le^x (D_2O)

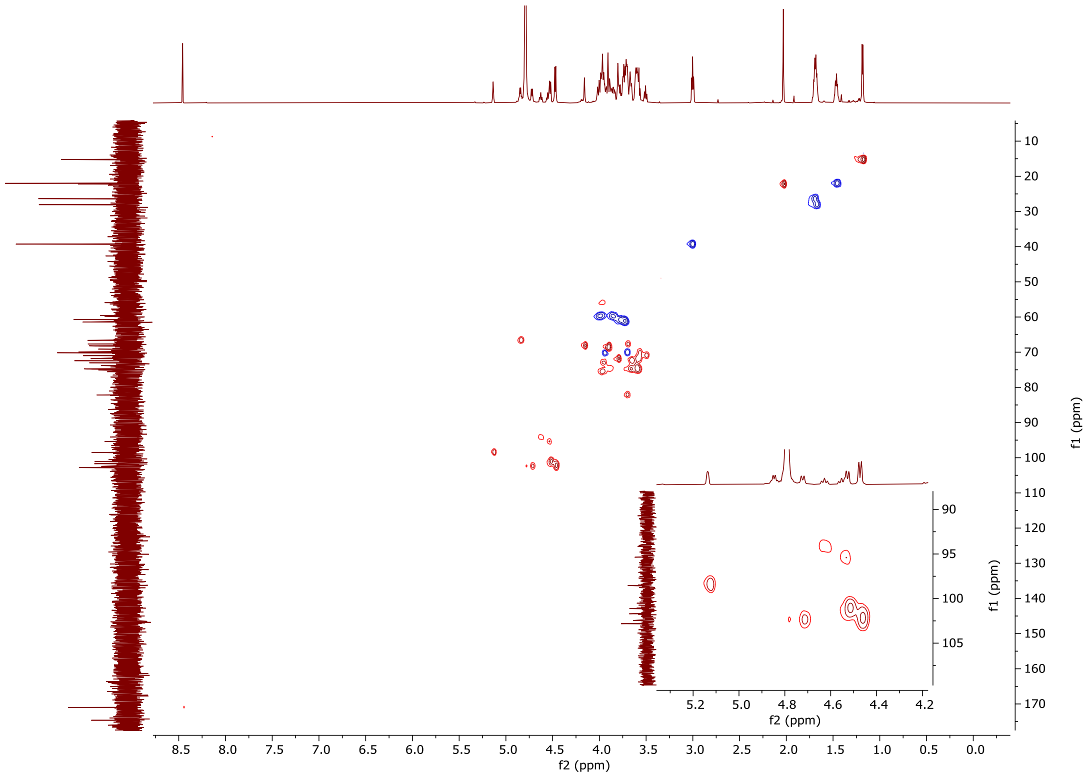

COSY NMR of F-Le^x (D_2O)

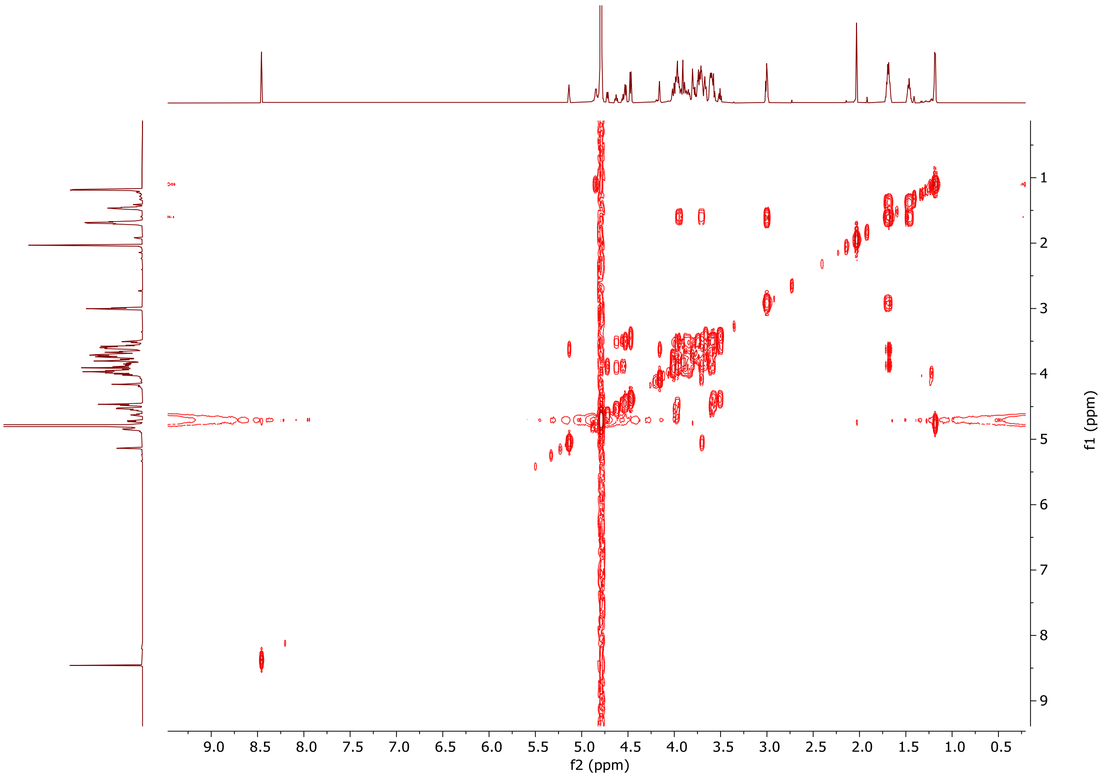

Synthesis of **F-H type 2**

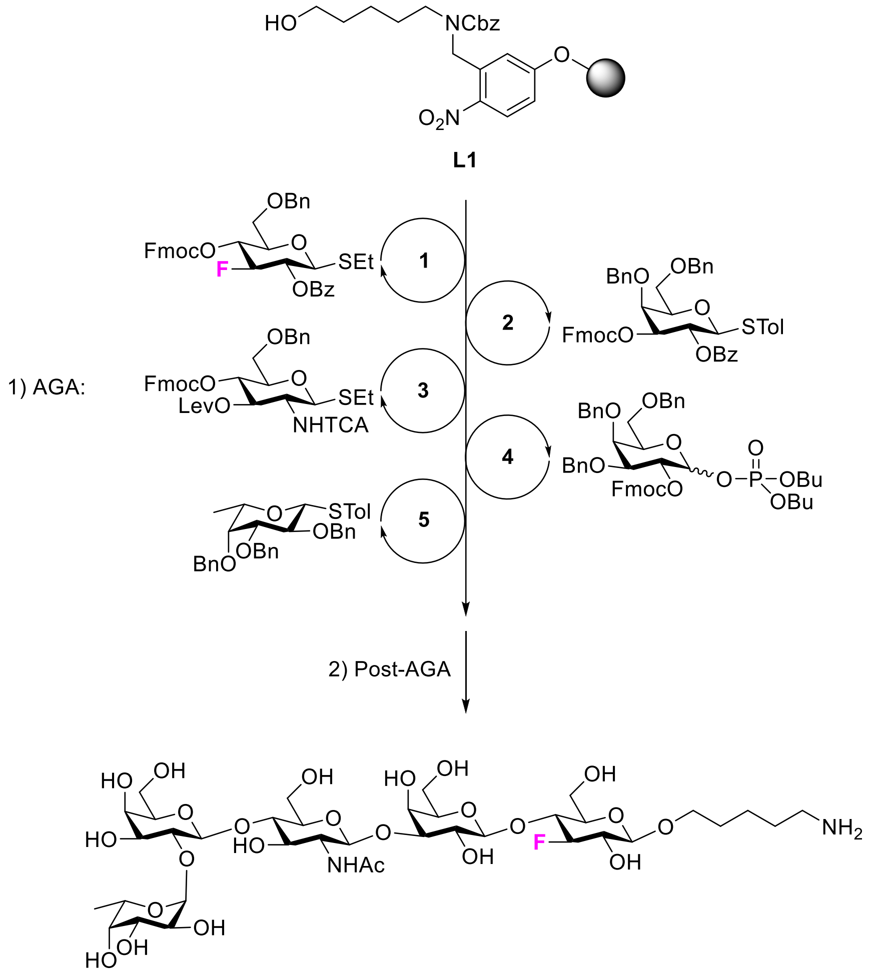

F-H type 2

Step	BB	Modules	Notes
AGA		**A**	**L1** swelling
	**1**	**B**, **C1**, **D**, **E1**	**C1**: (**1**, -20°C for 5 min, 0°C for 20 min)
	**2**	**B**, **C1**, **D**, **E1**	**C1**: (**2**, -20°C for 5 min, 0°C for 20 min)
	**3**	**B**, **C1**, **D**, **E1**	**C1**: (**3**, -20°C for 5 min, 0°C for 40 min)
	**4**	**B**, **C2**, **D**, **E1**	**C2**: (**4**, -35°C for 5 min, -15°C for 30 min)
	**5**	**B**, **C1**, **D**, **E2**	**C1**: (**5**, -40°C for 5 min, -20°C for 20 min)
Post-AGA		**F**, **G**, **H1**, **I**	**F:** (2 d)<br>**H1**: (3 d)<br>**I**: (Method C)

Automated synthesis, global deprotection, and purification afforded **F-H type 2** as a white solid (1.3 mg, 10% overall yield).

Analytical data for **F-H type 2**:

^{1}H NMR (700 MHz, Deuterium Oxide) δ 5.32 (s, 1H, **H-1 Fuc α-1,2**), 4.71 (d, *J* = 8.5 Hz, 1H, **H-1**), 4.68 – 4.44 (m, 4H, 4x **H-1**, H-3 Glc), 4.23 (q, *J* = 6.5 Hz, 1H, H-5 Fuc α-1,2), 4.20 – 3.64 (m, 25H), 3.64 – 3.50 (m, 2H), 3.47 (m, 1H), 3.01 (t, *J* = 7.6 Hz, 2H, CH_2-NH_3^+ linker), 2.05 (s, 3H, CH_3 NHAc), 1.80 – 1.62 (m, 4H, 2x CH_2 linker), 1.46 (p, *J* = 8.0 Hz, 2H, CH_2 linker), 1.27 – 1.20 (d, *J* = 6.1 Hz, 3H, CH_3 Fuc). ^{13}C NMR (151 MHz, Deuterium oxide) δ 170.79 (s, *C*=O NHAc), 102.82 (s, **C-1**), 102.68 (s, **C-1**), 101.13 (s, **C-1**), 100.19 (s, **C-1**), 99.35 (s, **C-1 Fuc α-1,2**), 94.75 (d, *J* = 196.3 Hz, C-3 Glc), 82.07, 76.39, 75.85, 75.58, 75.19, 75.04, 74.70, 73.76, 73.46, 72.00, 71.61, 70.15 (s, CH_2-O linker), 69.94, 69.55, 69.05, 68.14, 66.87 (s, C-5 Fuc α-1,2), 61.05, 60.68, 59.78, 55.33, 39.28 (s, CH_2-NH_3^+ linker), 28.06 (s, CH_2 linker), 26.33 (s, CH_2 linker), 22.13 (s, CH_3 NHAc), 21.99 (s, CH_2 linker), 15.24 (s, CH_3 Fuc). ^{19}F NMR (564 MHz, Deuterium oxide) δ -192.17 (dt, *J* = 52.1, 14.1 Hz). (ESI-HRMS) m/z 941.404 $[M+H]^+$ ($C_{37}H_{66}FN_2O_{24}$ requires 941.398).

RP-HPLC of F-H type 2 (ELSD trace, Method A, t_R = 27.4 min)

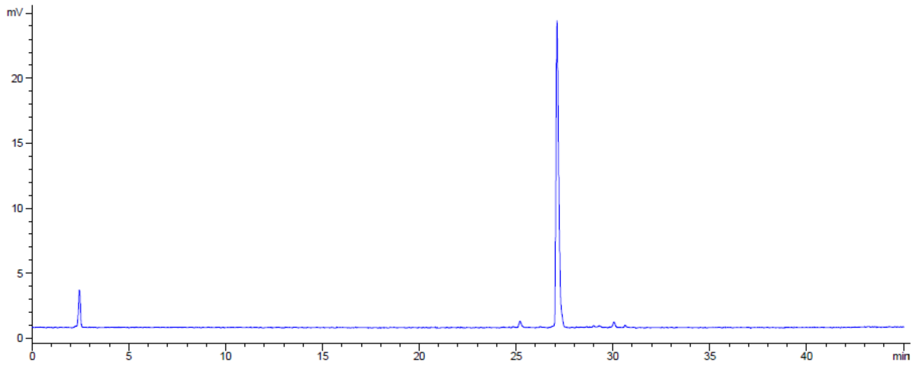

^1H NMR of F-H type 2 (700 MHz, D_2O)

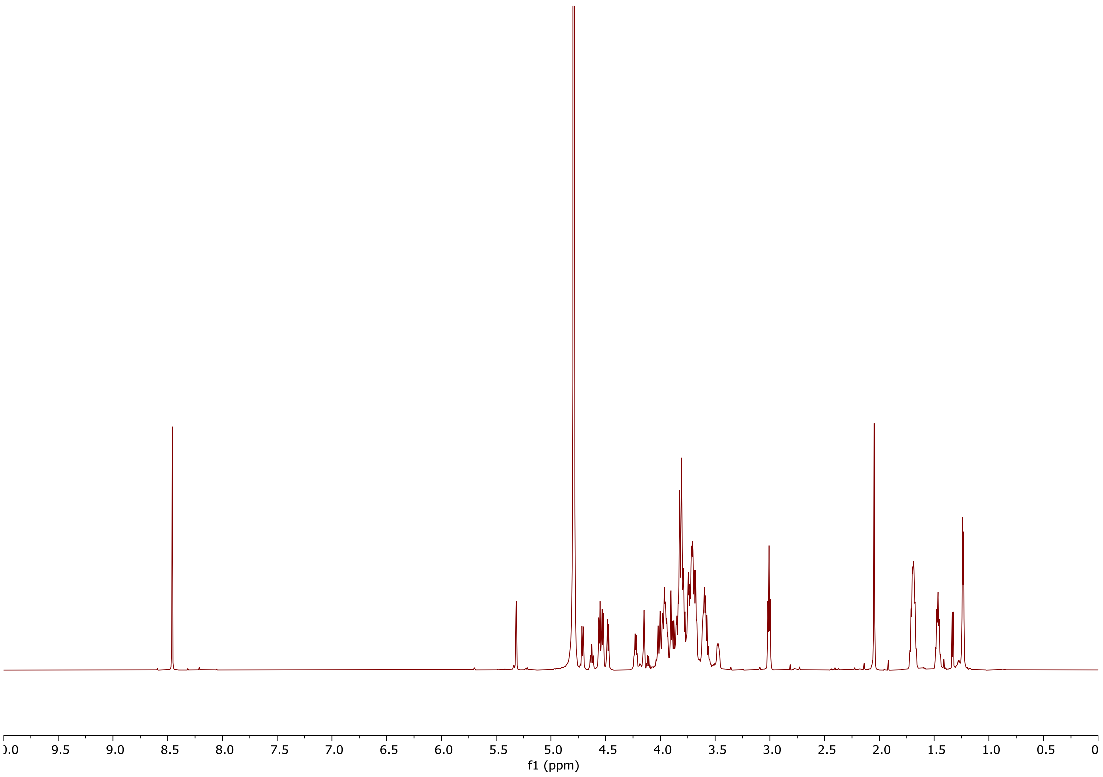

^{13}C NMR of F-H type 2 (151 MHz, D_2O)

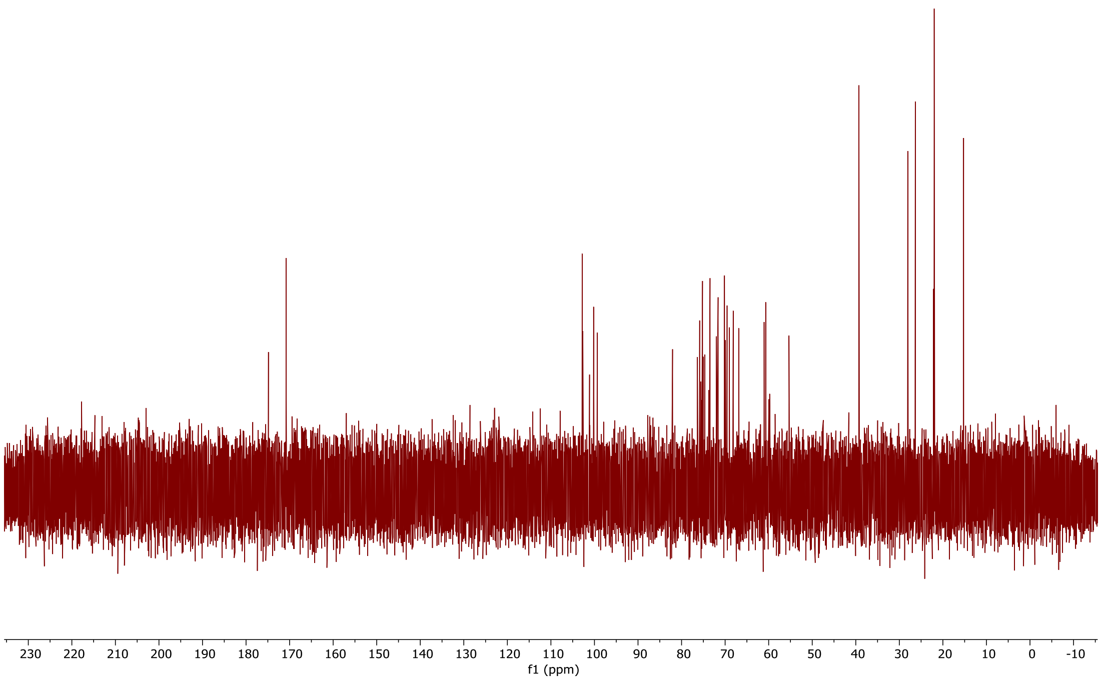

^{19}F NMR of F-H type 2 (564 MHz, D_2O)

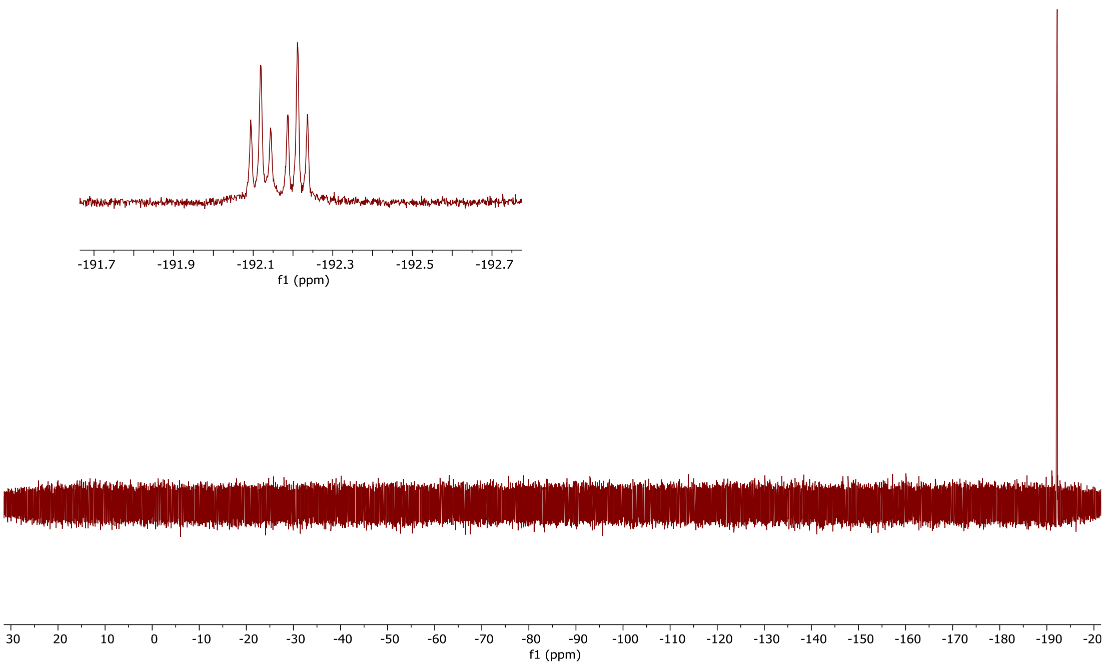

HSQC NMR of F-H type 2 (D_2O)

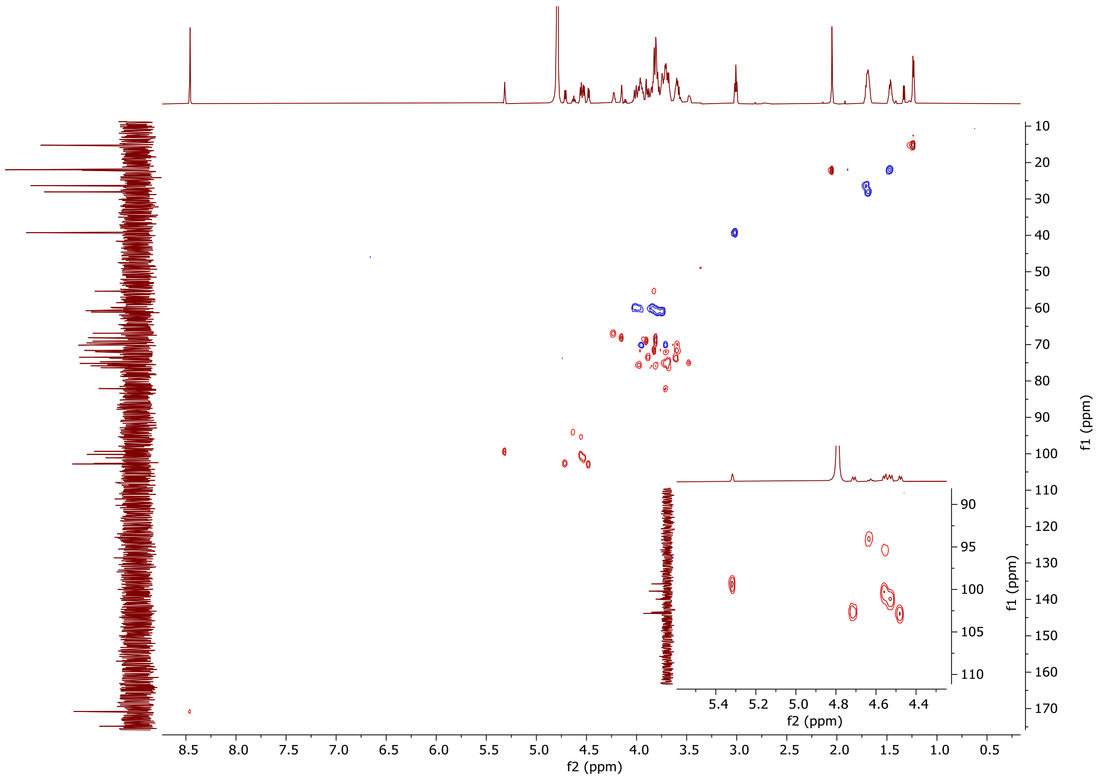

COSY NMR of F-H type 2 (D_2O)

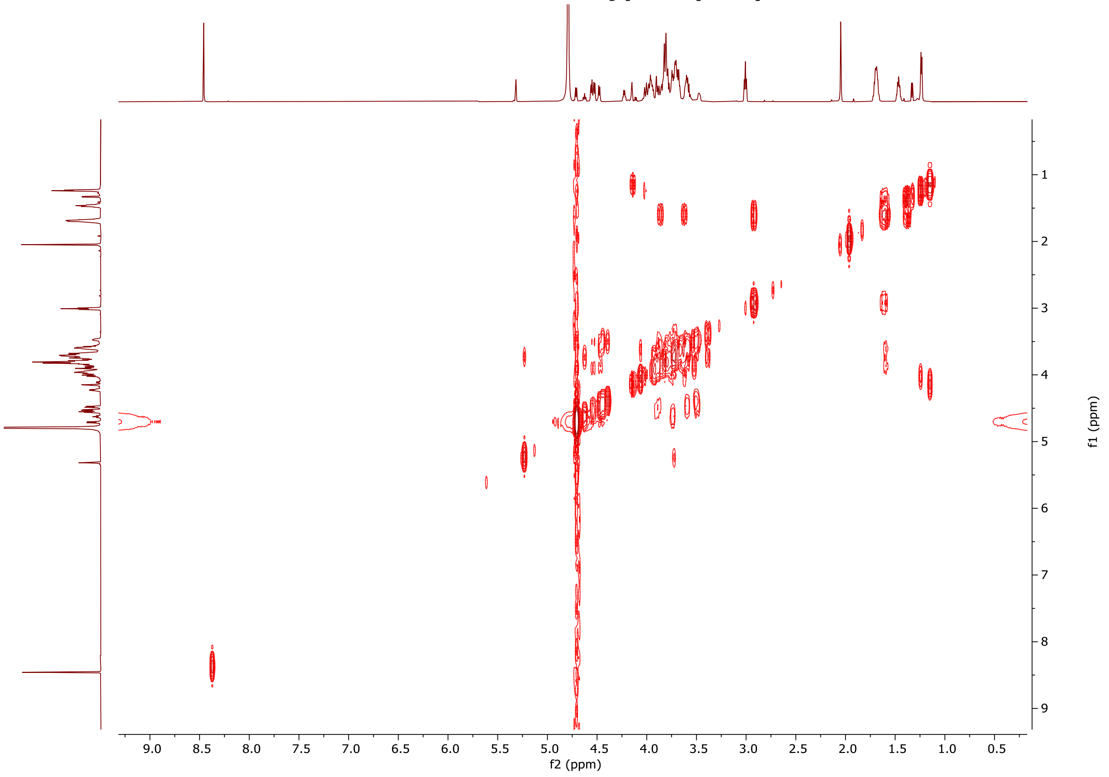

Synthesis of **F-Le^y**

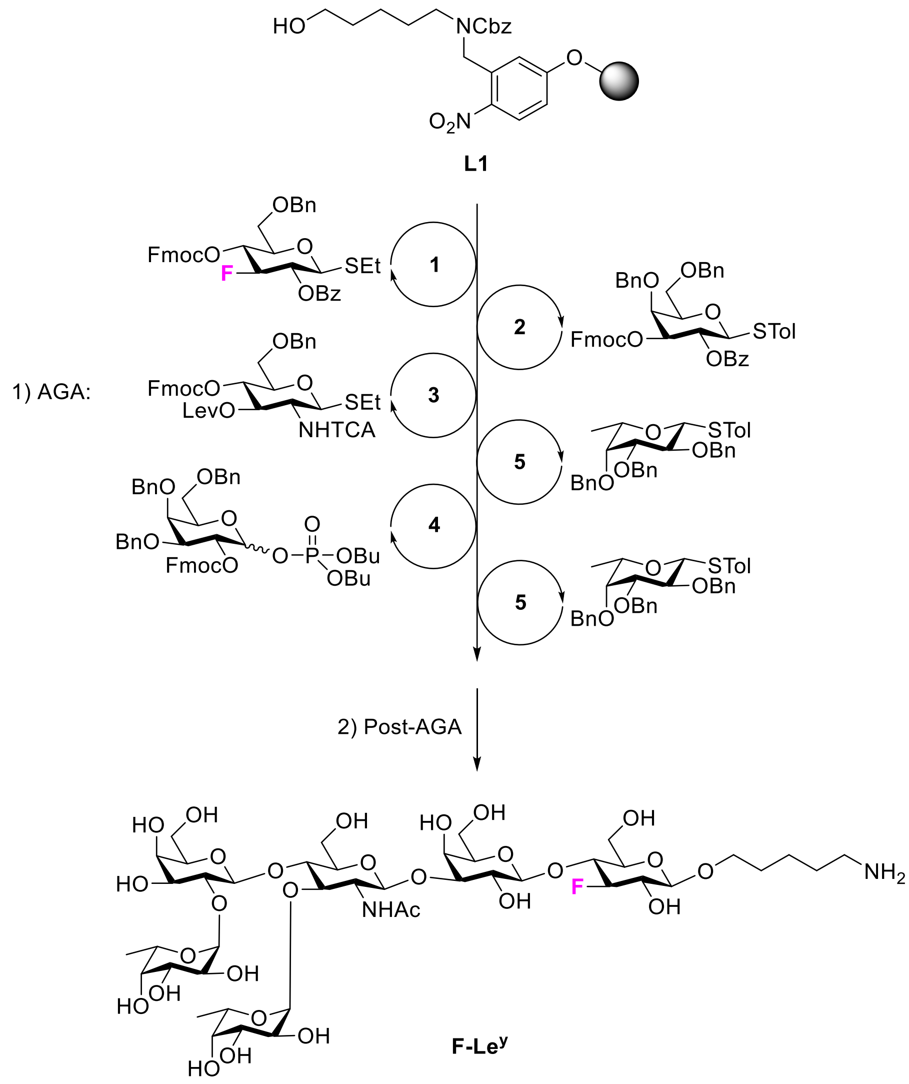

Step	BB	Modules	Notes
AGA		**A**	**L1** swelling
	**1**	**B**, **C1**, **D**, **E1**	**C1**: (**1**, -20°C for 5 min, 0°C for 20 min)
	**2**	**B**, **C1**, **D**, **E1**	**C1**: (**2**, -20°C for 5 min, 0°C for 20 min)
	**3**	**B**, **C1**, **D**, **E2**	**C1**: (**3**, -20°C for 5 min, 0°C for 40 min)
	**5**	**B**, **C1**, **D**, **E1**	**C1**: (**5**, -40°C for 5 min, -20°C for 20 min)
	**4**	**B**, **C2**, **D**, **E1**	**C2**: (**4**, -35°C for 5 min, -15°C for 30 min)
	**5**	**B**, **C1**, **D**	**C1**: (**5**, -40°C for 5 min, -20°C for 20 min)
Post-AGA		**F**, **G**, **H1**, **I**	**F:** (3 d)<br>**H1**: (3 d)

	**I**: (Method B, t_R = 22.0 min)

Automated synthesis, global deprotection, and purification afforded **F-Le^y** as a white solid (0.8 mg, 5% overall yield).

Analytical data for **F-Le^y**:

^{1}H NMR (700 MHz, Deuterium Oxide) δ 5.28 (d, *J* = 3.6 Hz, 1H, **H-1 Fuc α-1,2**), 5.12 (d, *J* = 4.0 Hz, 1H, **H-1 Fuc α-1,3**), 4.89 (q, *J* = 6.8 Hz, 1H, H-5 Fuc **α-1,3**), 4.73 (d, *J* = 8.4 Hz, 1H, **H-1**), 4.59 (dt, *J* = 52.3, 8.8 Hz, 1H, H-3 Glc), 4.54 – 4.50 (m, 2H, 2x **H-1**), 4.48 (d, *J* = 7.9 Hz, 1H, **H-1**), 4.26 (q, *J* = 6.6 Hz, 1H, H-5 Fuc α-1,2), 4.16 – 4.13 (m, 1H), 4.05 – 3.90 (m, 7H), 3.90 – 3.64 (m, 17H), 3.63 – 3.55 (m, 5H), 3.46 (ddd, *J* = 10.1, 5.2, 2.4 Hz, 1H), 3.05 – 2.99 (m, 2H, CH_2-NH_3^+ linker), 2.03 (s, 3H, CH_3 NHAc), 1.69 (dp, *J* = 13.8, 7.1 Hz, 4H, 2x CH_2 linker), 1.47 (qd, *J* = 9.6, 8.9, 6.5 Hz, 2H, CH_2 linker), 1.27 (d, *J* = 6.6 Hz, 3H, CH_3 Fuc), 1.24 (d, *J* = 6.6 Hz, 3H, CH_3 Fuc). ^{13}C NMR (176 MHz, Deuterium oxide) δ 170.92 (s, *C*=O NHAc), 102.88 (s, **C-1**), 101.20 (s, **C-1**), 101.13 (s, **C-1**), 100.22 (s, **C-1**), 99.44 (s, **C-1 Fuc α-1,2**), 98.60 (s, **C-1 Fuc α-1,3**), 94.83 (d, *J* = 182.8 Hz, C-3 Glc), 82.16, 76.40, 75.63, 75.39, 74.87, 74.76, 73.88, 73.83, 73.56, 73.09, 71.95, 71.85, 71.72, 70.22 (s, CH_2-O linker), 70.02, 69.74, 69.19, 68.75, 68.29, 68.22, 67.72, 66.92 (s, C-5 Fuc α-1,2), 66.80 (s, C-5 Fuc α-1,3), 61.48, 60.75, 59.86, 59.80, 56.15, 39.35 (s, CH_2-NH_3^+ linker), 28.13 (s, CH_2 linker), 26.40 (s, CH_2 linker), 22.28 (s, CH_3 NHAc), 22.06 (s, CH_2 linker), 15.46 (s, CH_3 Fuc), 15,44 (s, CH_3 Fuc). ^{19}F NMR (564 MHz, Deuterium oxide) δ -192.16 (dt, *J* = 52.4, 14.1 Hz). (ESI-HRMS) m/z 1087.462 $[M+H]^+$ ($C_{43}H_{76}FN_2O_{28}$ requires 1087.456).

RP-HPLC of F-Le^y (ELSD trace, Method A, t_R = 23.0 min)

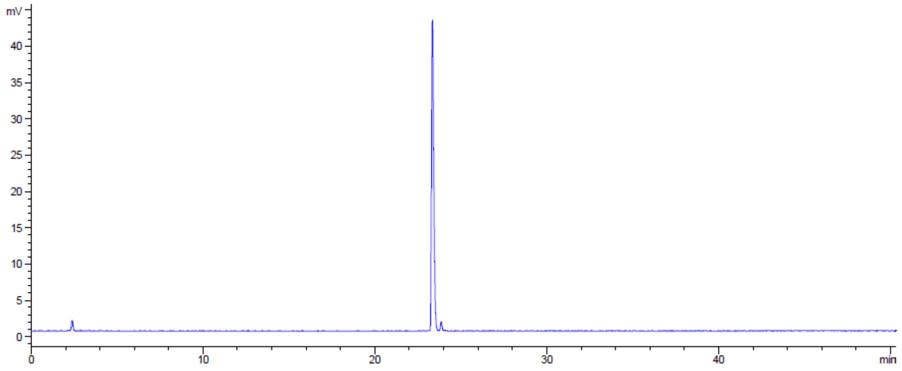

^{1}H NMR of F-Le^{y} (700 MHz, D_2O)

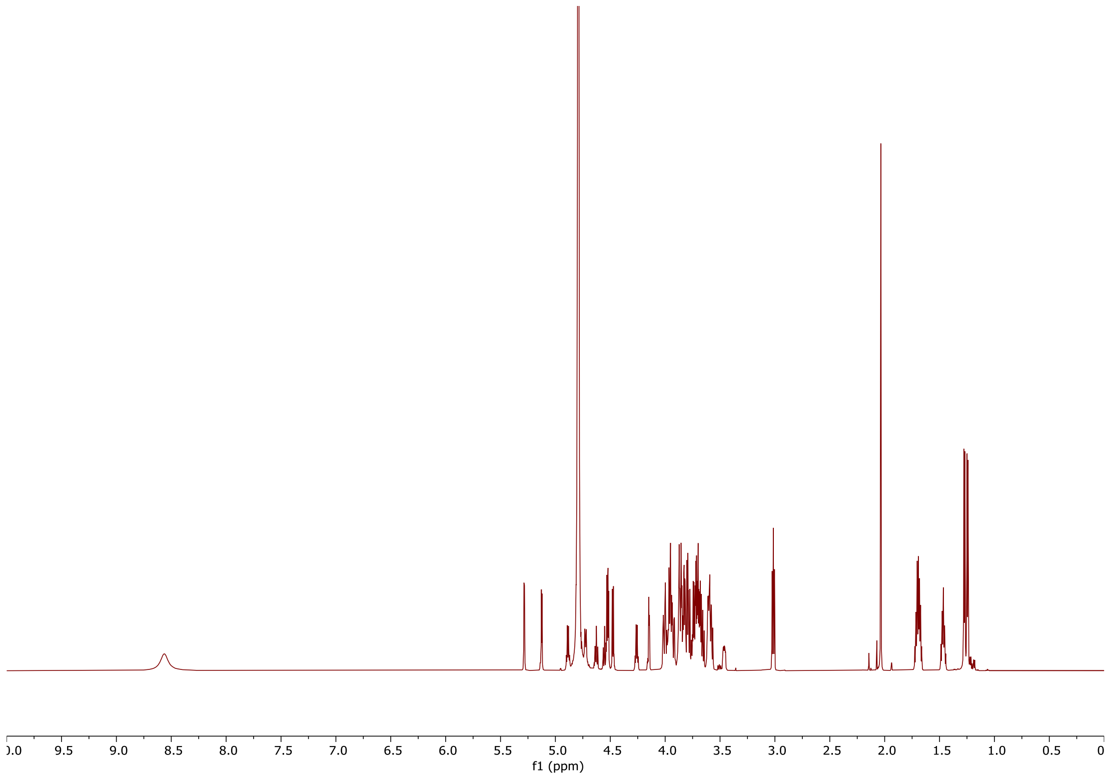

^{13}C NMR of F-Le^{y} (151 MHz, D_2O)

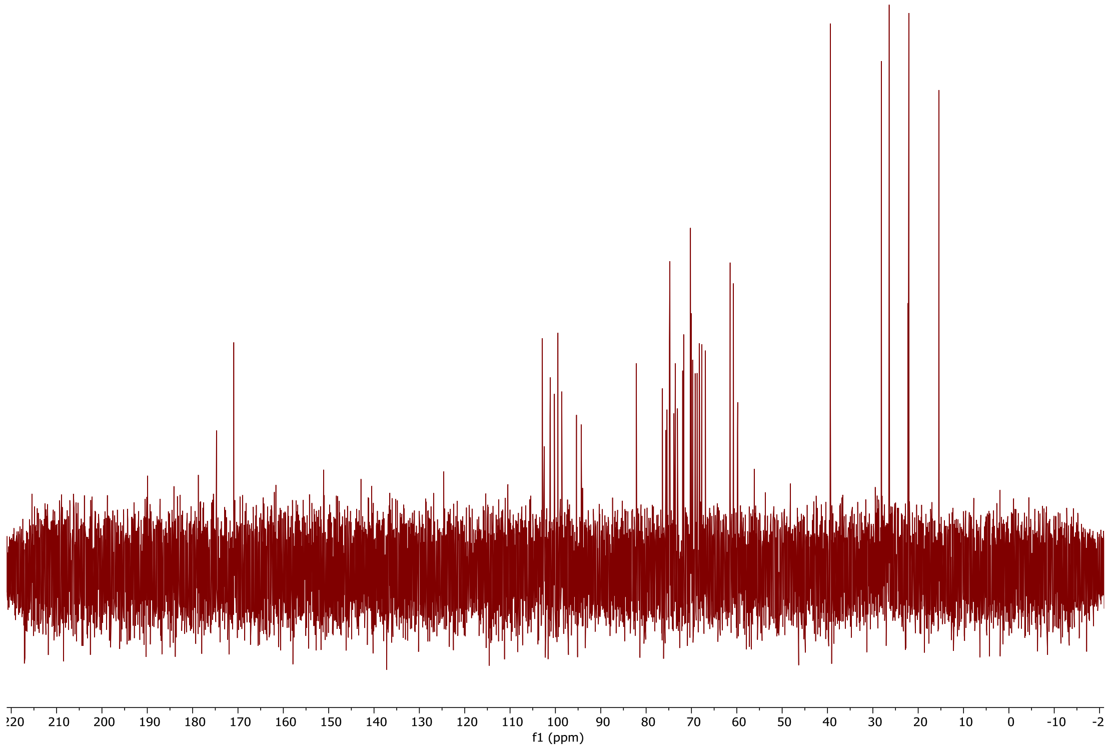

^{19}F NMR of F-Le^{y} (564 MHz, D_2O)

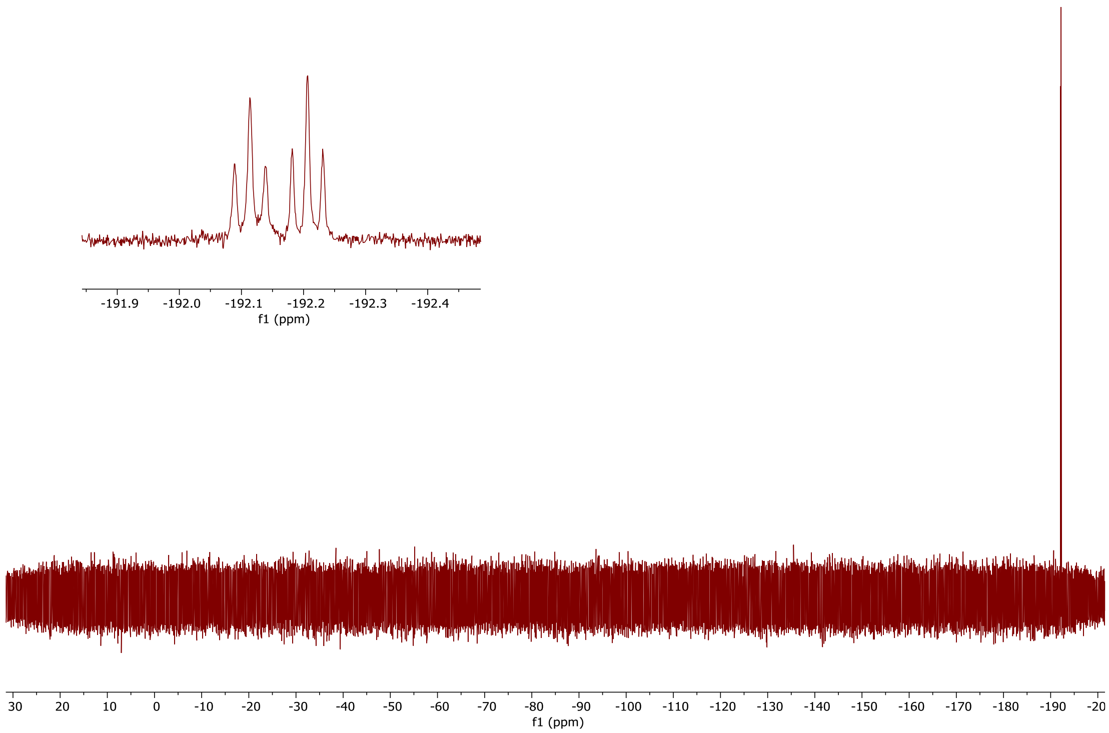

HSQC NMR of F-Le^{y} (D_2O)

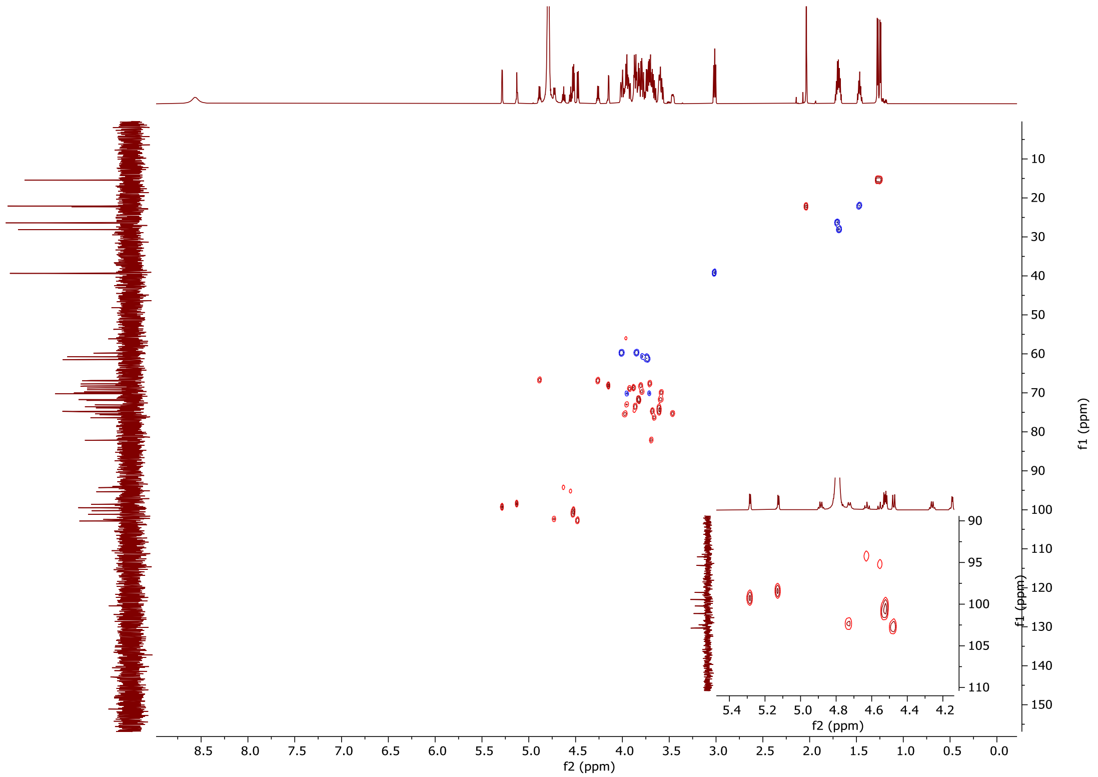

COSY NMR of F-Le^y (D_2O)

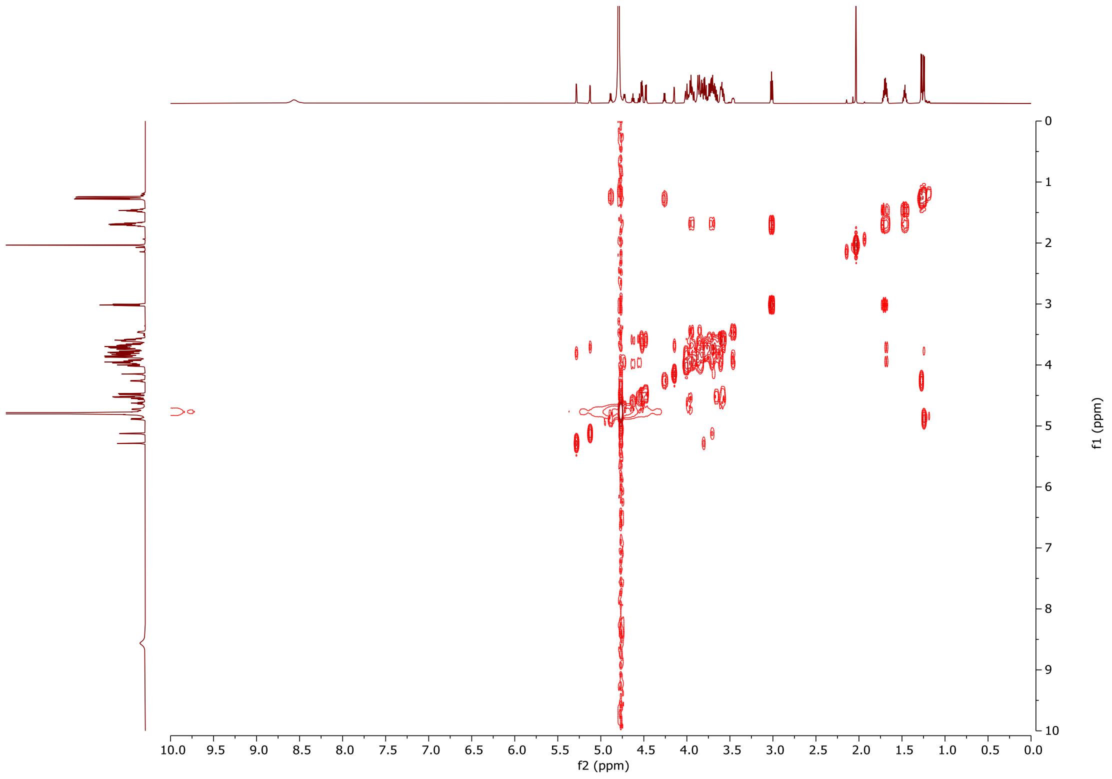

Synthesis of **CF_3-H type 2**

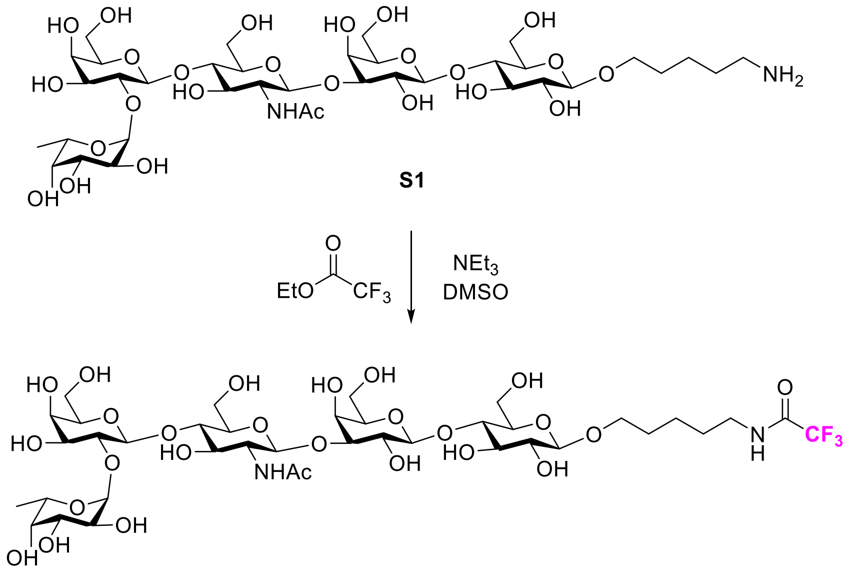

S1 was prepared by automated synthesis according to previously established protocols.[2]

S1 (250 µg, 0.27 µmol) was dissolved in DMSO (150 µL) and a triethylamine solution (10 µL, 0.1 M in DMSO, 1 µmol) was added. A solution of ethyltrifluoroacetate (20 µL, 0.1 M in DMSO, 2 µmol) was added and the mixture stirred at RT overnight. The reaction was then diluted with water, lyophilized, and purified by RP HPLC (Method B, t_R = 38.6 min). **CF_3-type 2** was obtained as a white solid (140 µg, 51% yield).

Analytical data for **CF_3-H type 2**:

^{1}H NMR (600 MHz, Deuterium Oxide) δ 5.32 (d, *J* = 3.1 Hz, 1H, **H-1 Fuc α-1,2**), 4.72 (d, *J* = 8.4 Hz, 1H, **H-1**), 4.56 (d, *J* = 7.7 Hz, 1H, **H-1**), 4.49 (d, *J* = 8.0 Hz, 1H, **H-1**), 4.46 (d, *J* = 7.9 Hz, 1H, **H-1**), 4.23 (q, *J* = 6.6 Hz, 1H, H-5 Fuc α-1,2), 4.16 (d, *J* = 3.4 Hz, 1H), 4.02 – 3.87 (m, 5H), 3.87 – 3.57 (m, 22H), 3.51 – 3.45 (m, 1H), 3.35 (t, *J* = 7.0 Hz, 2H, CH_2-NH_3^+ linker), 3.33 – 3.29 (m, 1H), 2.06 (s, 3H, CH_3 NHAc), 1.70 – 1.59 (m, 4H, 2x CH_2 linker), 1.42 (p, *J* = 7.7 Hz, 2H, CH_2 linker), 1.24 (d, *J* = 6.6 Hz, 3H, CH_3 Fuc). ^{19}F NMR (564 MHz, Deuterium oxide) δ -75.92. (ESI-HRMS) m/z 1057.372 $[M+Na]^+$ ($C_{39}H_{65}F_3N_2O_{26}Na$ required1057.367).

RP-HPLC of the crude reaction mixture (ELSD trace, Method A)

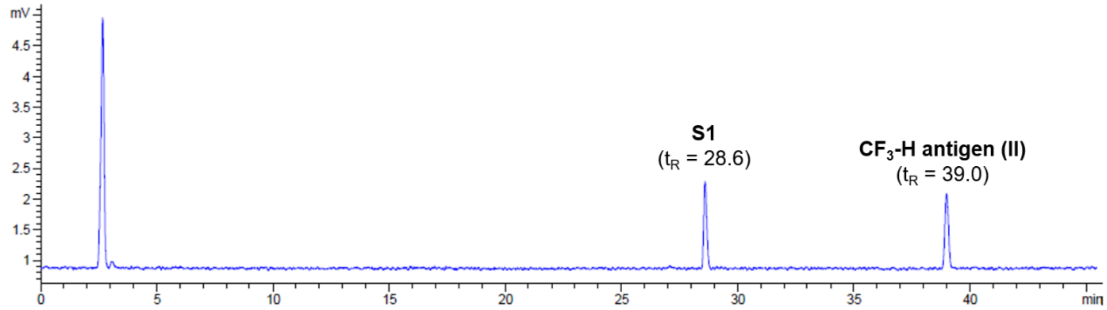

RP-HPLC of pure CF_3-H type 2 (ELSD trace, Method A, t_R = 39.1 min)

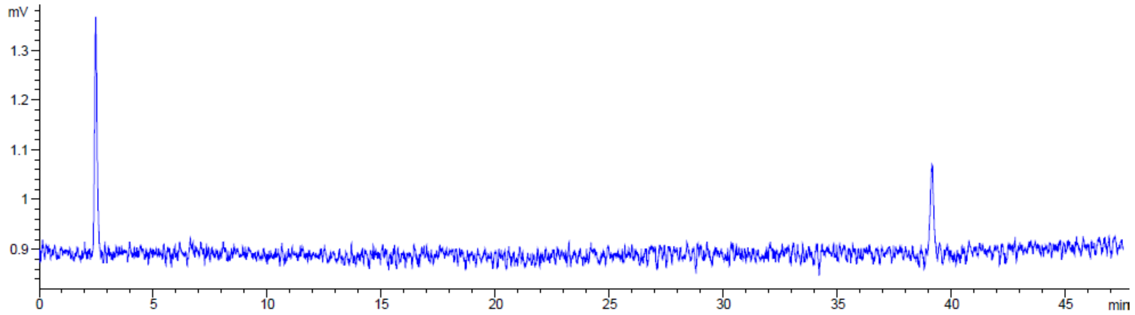

^{1}H NMR of CF_3-H type 2 (600 MHz, D_2O)

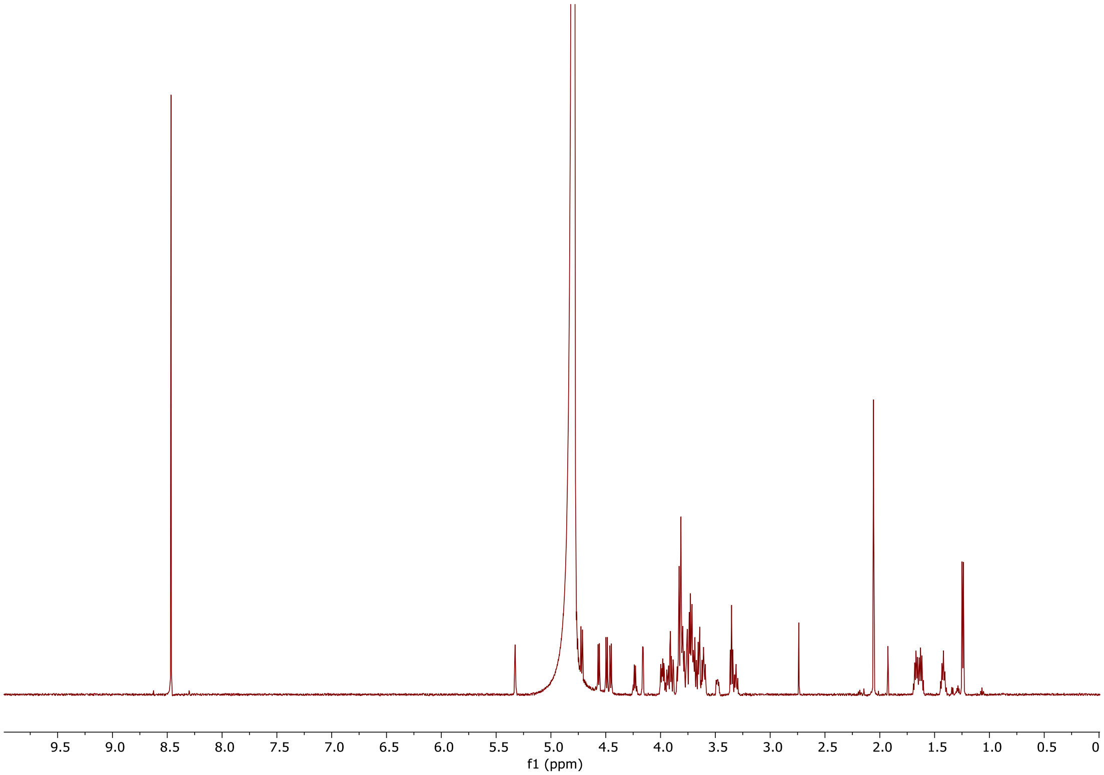

^{19}F NMR of CF_3-H type 2 (564 MHz, D_2O)

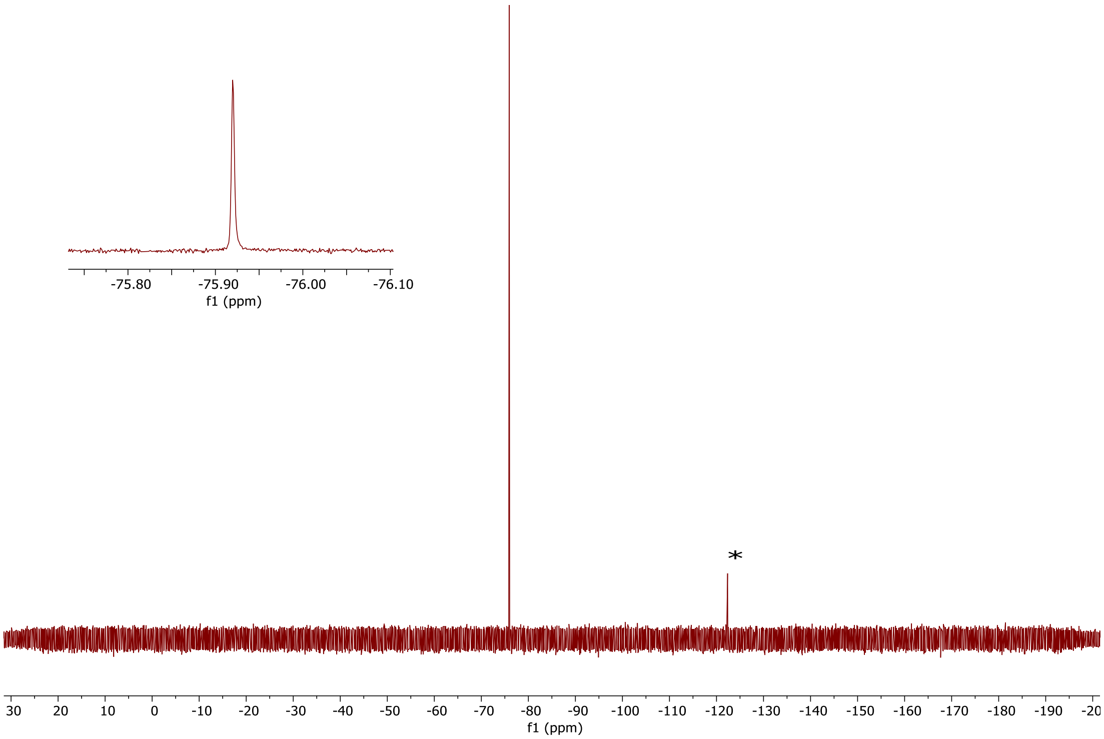

* The peak at -122.29 ppm is a fluoride impurity

D. NMR studies of glycan-protein interactions

General materials and methods

Chemicals

All commercial carbohydrates and building blocks were purchased from Carbosynth (UK) or TCI (Germany).

Recombinant proteins

The non-labelled or ^{15}N-labeled LecA[6], LecB[7] and BambL[8] were purified in soluble form as reported previously. LecA labelled with 5-fluorotryptophan (5FW) was expressed and purified as described previously.[9] Human Langerin ECD, DC-SIGN CRD and ECD constructs were expressed in inclusion bodies and prepared as described previously.[10,11]

^{19}F NMR

^{19}F NMR was used to screen F-glycans for bacterial (LecA, LecB, BambL), mammalian lectins (Langerin, DC-SIGN), and sialyltransferases (α(2,3)-sialyltransferase from Pasteurella multocida (Pmα23ST) and α(2,6)-sialyltransferase from Photobacterium damsela (Pdα26ST)). The enzymes α(2,3)-sialyltransferase from *Pasteurella multocida* (Pmα23ST, *E. coli* EC no. 2.4.99.4), and α(2,6)-sialyltransferase from *Photobacterium damsela* (Pdα26ST, *E. coli* EC no. 2.4.99.1) were purchased from Sigma Aldrich. All experiments were performed on a Bruker Ascend™700 (AvanceIII HD) spectrometer equipped with a 5 mm TCI700 CryoProbe™ in 3 mm tubes (Norell S-3-800-7) at 298 K. The proteins were screened at 10 µM (or 67 mU/mL for enzymes) against F-glycans: **F-Lac**, **F-nLac$_4$**, **F-Le^x**, **F-H type 2** and **F-Le^y** in 25 mM Tris-HCl pH 7.8, 150 mM NaCl, 10% D_2O, 25 µM TFA and 5 mM $CaCl_2$ at 298 K. For this, proteins were mixed at 1:1 in volume ratio with the F-glycans resulting in the concentrations of 10 µM (or 67 mU/mL for enzymes) and 50 µM, respectively. The enzymes were screened in the absence of donor (*i.e.* CMP-Neu5Ac). ^{19}F spectra were recorded with 512 scans, a spectral width of 10 ppm, a transmitter offset at -190 ppm, acquisition time of 2 s and 1 s relaxation time. T2-filtered spectra were recorded using a CPMG pulse sequence with a 180° pulse repetition rate of 0.38 s using same acquisition and relaxation times.[12,13] ^{19}F R_2-filtered experiments for 0.6 mM **F-H type 2** and 0.15 mM LecA were performed with a CPMG pulse sequence with a 180° pulse repetition rate of 0, 0.24, 0.48, 0.72, 0.96 and 1.2 s using the same buffer and acquisition conditions as described above. ^{19}F and CPMG NMR spectra of 50 µM **CF$_3$-H type 2** were recorded in presence of 10 µM DC-SIGN ECD and 5 µM BambL with 16 scans, a spectral width of 10 ppm, a transmitter offset at -75 ppm, acquisition time of 2 s and 1 s relaxation time. Data

were recorded without proton decoupling. All spectra were analyzed in MestReNova 11.0.0 (Mestrelab Research SL). The binding strength (**Figure 1B**) was defined depending on the changes observed in the NMR after addition of the protein. A decrease in peak intensity higher than -25% or a CSP higher than 0.01 ppm in the normal ^{19}F NMR was interpreted as strong binding (blue), a decrease in peak intensity higher than -25% in the CPMG filtered ^{19}F NMR as weak/medium binding (light blue), and a decrease in peak lower than -25% in normal or CPMG filtered ^{19}F NMR as no binding (white).

K_d determination with F-glycans in ^{19}F NMR

To derive the affinities of BambL to **F-H type 2** and **F-Le^y**, we recorded ^{19}F NMR of 50 µM compound alone and in presence of BambL (6.25 µM to 100 µM) in 25 mM Tris-HCl pH 7.8, 150 mM NaCl (TBS) with 25 µM TFA and 10% D_2O at 298 K. ^{19}F spectra were recorded with 512 scans, a spectral width of 5 ppm, a transmitter offset at -191 ppm, acquisition time of 2 s and 1 s relaxation time. All spectra were normalized to internal reference trifluoroacetic acid (TFA) at -75.6 ppm and analyzed for the changes in the peak intensities. The decreasing intensity of F-glycan peak in the free state was followed to determine the K_d values of **F-H type 2** and **F-Le^y**. Next, we normalized the changes in the fluorine peak intensities ($I_{normalized}$) following the equation (1) resulting in values plotted on Y-axis.

$$I_{normalized} = \frac{I_0 - I_{measured}}{I_0} \quad (1),$$

where I_0 was F-glycan in the reference spectrum without protein, $I_{measured}$ was F-glycan with protein. The K_d values were calculated according to the one- and two−site binding models in Origin(Pro) 2020b (OriginLab Corp., USA) from three independent titrations.

^{1}H-^{15}N HSQC and TROSY NMR

To validate binding F-glycans to DC-SIGN CRD and BambL, we ^{15}N-labeled proteins for ^{1}H-^{15}N HSQC and TROSY NMR, respectively. All ^{1}H-^{15}N HSQC and TROSY experiments were measured on a Bruker Ascend™700 (AvanceIII HD) spectrometer equipped with a 5 mm TCI700 CryoProbe™ in 3 mm tubes (Norell S-3-800-7) at 298 K and 310 K, respectively.

Briefly, ^{15}N DC-SIGN HSQC and ^{15}N BambL TROSY experiments were recorded with 100 µM protein in 20 mM HEPES pH 7.4, 150 mM NaCl with 5 mM $CaCl_2$, 10% D_2O and 100 µM 4,4-dimethyl-4-silapentane-1-sulfonic acid (DSS) as internal reference. A ^{1}H-^{15}N HSQC pulse sequence *hsqcf3gpph19* with 128 increments and 8 scans per increment was applied for ^{15}N DC-SIGN CRD. A ^{1}H-^{15}N TROSY pulse sequence *trosyf3gpphsi19* with 128 increments and 32 scans per increment was applied for ^{15}N BambL. We recorded ^{15}N HSQC NMR of DC-SIGN

CRD in presence of 1 mM **F-H type 2** and 250 µM **F-Le^{y}** or **F-Le^{x}**. ^{15}N TROSY NMR titration experiments of ^{15}N BambL were recorded with 30 to 600 µM **F-H type 2** or 90 to 880 µM **F-Le^{y}**. Data were processed with NMRpipe[14] and further analyzed with CcpNmr analysis.[15]

^{1}H-^{15}N HSQC DC-SIGN CRD resonances were assigned as reported previously.[11] ^{1}H-^{15}N TROSY BambL resonances were indexed with IDs from 1 to 72 due to a lack of the protein backbone resonance assignment. Next, resonance IDs from BambL and DC-SIGN CRD spectra were transferred to the spectra obtained in the presence of F-glycan for a comparison of the changes in the peak intensities and chemical shift perturbations (CSPs) indicating a slow and fast exchange regimes on the chemical shift timescale, respectively. The changes in peak intensities upon addition of F-glycans were calculated in CcpNmr analysis followed by normalization according to Equation (1). The K_d values were calculated according to the one−site binding model in Origin(Pro) 2020b (OriginLab Corp., USA).

The changes in chemical shift perturbations (CSPs) were calculated according to Equation (2):

$$\Delta\delta = \sqrt{\frac{1}{2}[\Delta\delta_H^2 + (\alpha\Delta\delta_N)^2]} \quad (2)$$

in which δ is the difference in chemical shift (in ppm) and α is an empirical weighting factor of 0.14 for all amino acid backbone resonances.[16] The threshold value was set based on three independent measurements of reference spectra to 0.01 ppm and 0.005 ppm for LecA, BambL and DC-SIGN CRD, respectively.

Protein-observed ^{19}F (PrOF) NMR

To validate binding of **F-H type 2** to LecA, we recorded protein-observed ^{19}F (PrOF) NMR with recombinant 5FW LecA as reported previously.[9] Briefly, 150 µM 5FW LecA was recorded alone and in presence of 1 mM **F-H type 2** in 25 mM Tris-HCl pH 7.8, 150 mM NaCl, 5 mM $CaCl_2$ with 10% D_2O and 50 µM TFA as internal reference at 310 K. Data analysis was performed in MestReNova 11.0.0 (Mestrelab Research SL) after applying the exponential function (30 Hz) and baseline correction.

Real-time ^{19}F NMR kinetic measurement

The enzymes β-galactosidase (*E. coli*, CAS: 9031-11-2), and α(2,3)-sialyltransferase from *Pasteurella multocida* (Pmα23ST, *E. coli* EC no. 2.4.99.4) were purchased from Sigma Aldrich. The enzymatic reactions with β-galactosidase (0.15 µM), and Pmα23ST (100 mU/mL) were performed at 310 K in 25 mM Tris-HCl pH 7.8, 150 mM NaCl with 2 mM $MgCl_2$, 100 µM TFA and 10% D_2O. The concentration of **F-Lac** was 250 µM. The reactions with β-galactosidase were monitored every 149 s. All data were recorded without proton decoupling with 32 scans,

acquisition time of 2 s and relaxation delay of 1 s. All spectra were referenced to TFA at -75.6 ppm. To derive the kinetics we used the peak integral of product at -194.8 ppm to derive its concentration. Data were analyzed with nonlinear least-squares methods in the GraphPad Prism 8 software. The best fit of the experimental data provides the value of K_M the Henri−Michaelis−Menten equation. To monitor the sialylation of 250 µM **F-Lac** and **F-nLac$_4$** with Pmα23ST, we used the buffer conditions reported above with 275 µM CMP-Neu5Ac and recorded data at time points: 0 min, 13 min, 32 min, and 246 min. All data were recorded without proton decoupling with 128 scans, acquisition time of 2 s and relaxation delay of 1 s. All spectra were referenced to TFA at -75.6 ppm. To determine the yield of this reaction, we used the ratio of peak integrals of substrate and product at -192.1 ppm and -192.3 ppm, respectively.

Note to the ^{19}F NMR experiments

All the experiments described in this study were performed without ^{1}H-^{19}F decoupling due to the lack of the specific hardware component in the spectrometer used. The ^{19}F NMR analysis would benefit in sensitivity and resolution when recording ^{19}F{^{1}H } NMR spectra.

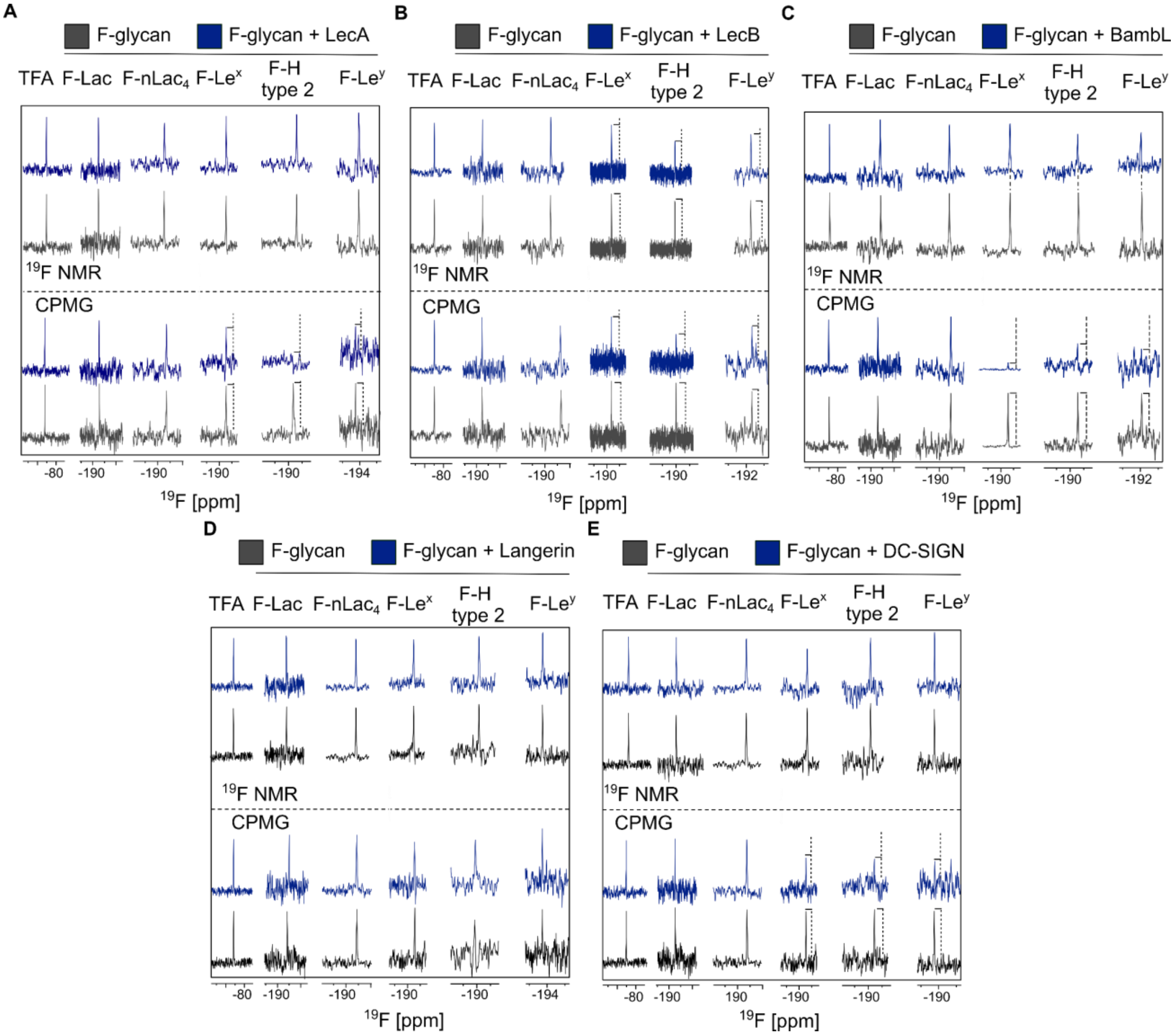

Figure S2 *Binding of F-glycans to mammalian and bacterial lectins.* ^{19}F and CPMG NMR spectra of F-glycans alone (*gray*) and in presence of DC-SIGN ECD (*blue*). A chemical shift perturbation of fucosylated Lewis antigens in presence of BambL (**C**, *upper panel*) in ^{19}F NMR is shown (*dashed line*). Decrease in peak intensity (*dashed line*) in CPMG NMR indicates binding of fucosylated Lewis antigens to bacterial: LecA (**A**), LecB (**B**) and BambL (**C**, *lower panel*) and mammalian: Langerin ECD (**D**) DC-SIGN ECD (**E**) lectins. No binding to F-glycans has been observed in presence of Langerin ECD.

Differences in signal-to-noise ratio were observed for the spectra acquired with different quality of shimming.

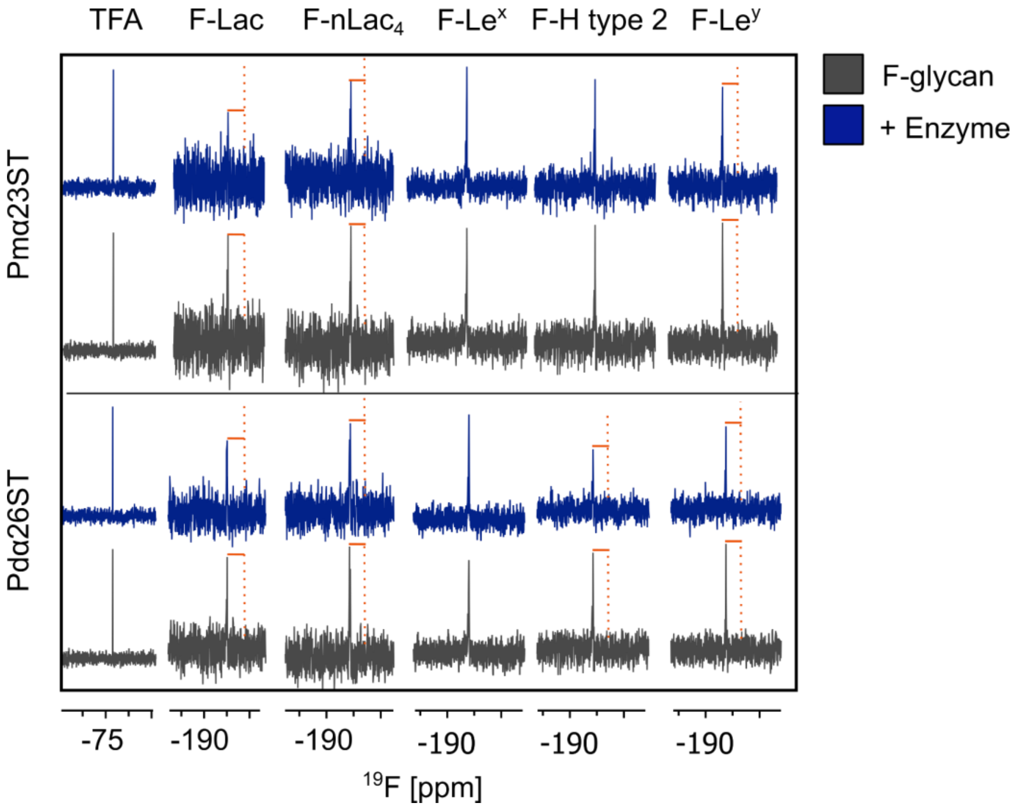

Figure S3 *Binding of sialyltransferases to F-glycans.* CPMG NMR of F-glycans alone and in presence of Pmα23ST or Pdα26ST in the absence of CMP-Neu5Ac donor. F-glycan binding to both enzymes is shown as a reduction in peak intensity in presence of enzymes (*orange*).

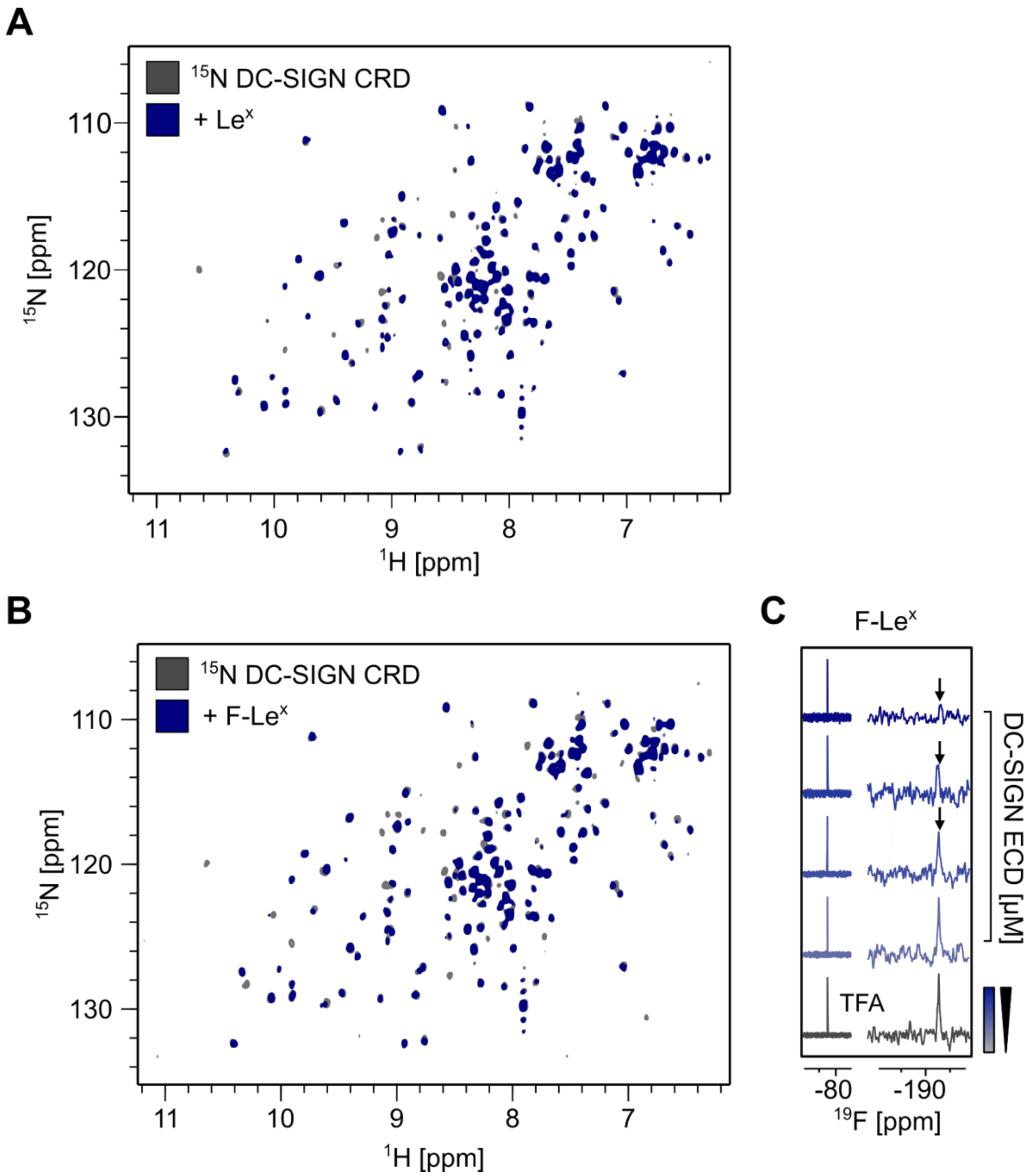

Figure S4 *Interaction of* ***F-Le^x*** *and* ***Le^x*** *with a mammalian lectin DC-SIGN*. **A** HSQC NMR of ^{15}N-labeled DC-SIGN CRD binding to **Le^x**. **B** HSQC NMR of ^{15}N-labeled DC-SIGN CRD binding to **F-Le^x**. **C** CPMG NMR spectra of 25 µM **F-Le^x** alone (*gray*) and in presence of increasing concentration of DC-SIGN ECD: 2.5, 5, 10, 25 and 50 µM (*blue*). The largest difference in a bound *vs* free state was observed at 50 µM DC-SIGN ECD.

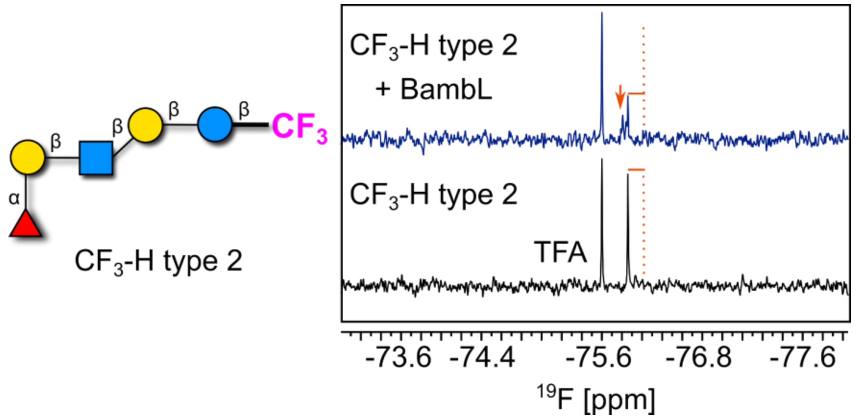

Figure S5 *Binding of* ***CF_3-H type 2*** *to BambL in ^{19}F NMR.* ^{19}F NMR spectrum of **CF_3-H type 2** alone and in presence of BambL (6 µM). Given that **CF_3-H type 2** undergoes a slow exchange in presence of BambL a new peak for the bound ligand arises (*arrow*). This proves that the CF_3 reporter does not have to be near the carbohydrate-binding site of a lectin and can be placed even in remote position (*i.e.* aminopentyl linker attached to the reducing end) of the glycan in order to be detected in ^{19}F NMR.

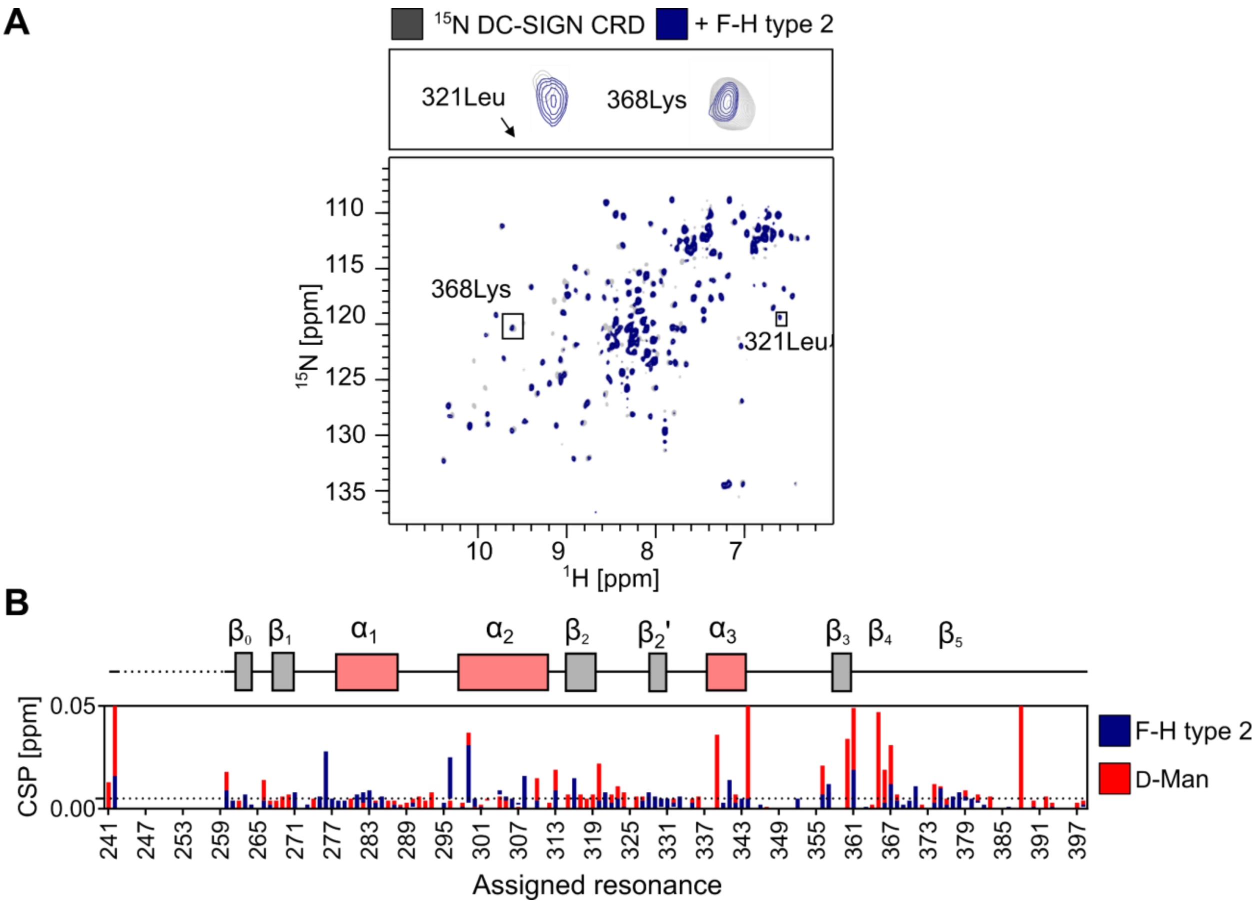

Figure S6 *Weak interaction of **F-H type 2** with DC-SIGN CRD*. **A** HSQC NMR of ^{15}N-labeled DC-SIGN CRD in complex with **F-H type 2**. **F-H type 2** promoted CSPs in the carbohydrate-binding site of DC-SIGN CRD (321Leu and Lys368) and remote parts of DC-SIGN CRD (*not shown*) similarly to D-mannose. **B** The magnitude of **F-H type 2** interaction with DC-SIGN CRD was comparable to D-mannose showing a weaker interaction of **F-H type 2** compared to **F-Le**$^{\mathbf{x}}$ or **F-Le**$^{\mathbf{y}}$.

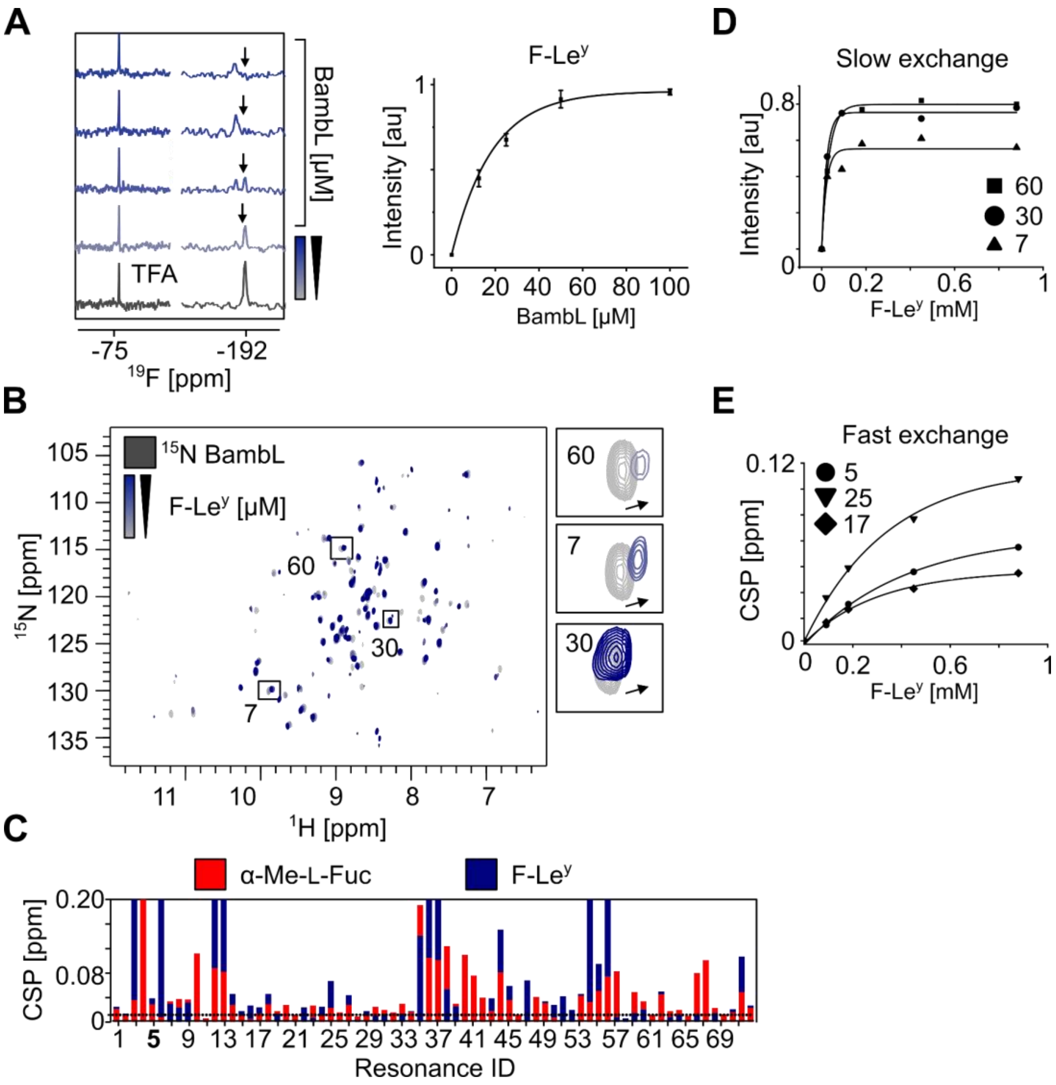

Figure S7 Interaction of ***F-Le^y*** *with a bacterial lectin BambL*. **A** ^{19}F and CPMG NMR screening of F-glycan (25 µM) alone and in presence of BambL (10 µM). DC-SIGN interacts with **F-Le^y** as shown in ^{19}F NMR titration spectra (*left panel*) and one-site-binding model fit used to derive the dissociation constant (K_d) of **F-Le^y** to BambL (*right panel*). The apparent K_d was calculated using changes in peak intensity (*arrow*) and estimated to be at P:L ratio of 1:0.38 which (referenced to the concentration of 25 µM **F-Le^y** in this assay) corresponds to K_d of 9.2 ± 1.5 µM. **B** TROSY NMR verified **F-Le^y** binding to ^{15}N-labeled BambL. Given that BambL has two binding sites, peaks showing a slow (60, 7 and 30), intermediate and fast exchange (5, 17 and 25) on the chemical shift timescale have been observed upon titration of **F-Le^y**. **C** CSP plot showing the resonances perturbed in presence of α-Me-L-fucose and **F-Le^y**. This verifies that **F-Le^y** targets the carbohydrate-binding site of BambL. Notably, the magnitude of **F-Le^y** promoted effects is similarly to L-fucose suggesting that the avidity effect does not contribute to **F-Le^y**

interaction with BambL, but is rather due to binding of L-fucose alone. One-site model for slow (**D**) and fast exchange (**E**) peaks was applied to derive the K_d values of 17±3 μM and 245±29 μM, respectively.

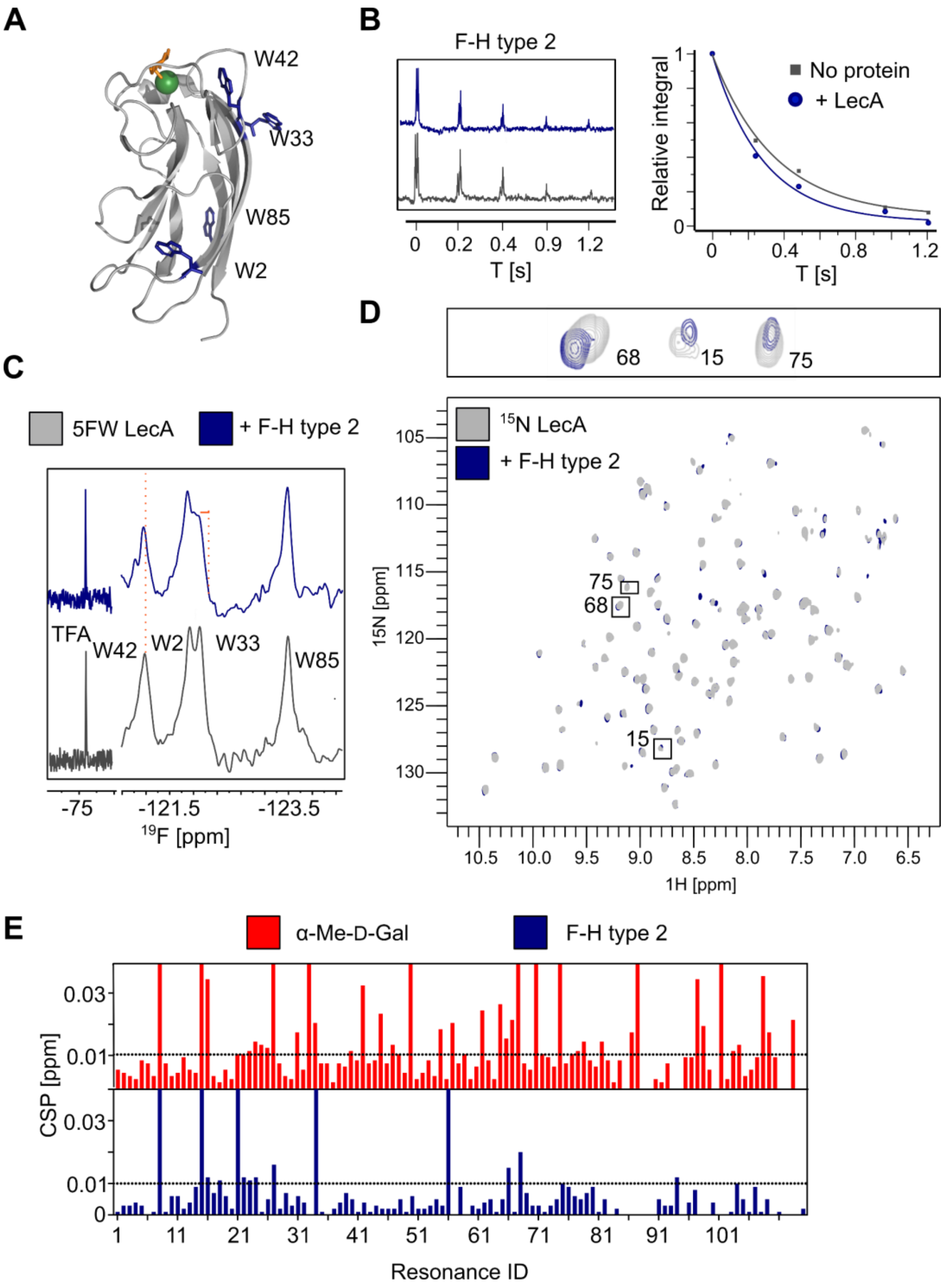

Figure S8 ***F-H type 2*** *binding to a bacterial lectin LecA*. **A** A ribbon diagram of the crystal structure of a LecA monomer (PDB: 1OKO) bound to galactose (*orange*) in a Ca^{2+}-dependent manner (*green sphere*). The four tryptophanes (*blue*) are labelled with 5-fluorotryptophanes for PrOF NMR. **B** ^{19}F R_2-filtered NMR assay performed on **F-H type 2** and the decay curves of 0.6 mM **F-H type 2** alone (*grey*) and in presence of 0.15 mM LecA (*blue*). **C** PrOF NMR spectrum of 5-fluorotryptophan labelled (5FW) LecA alone (*grey*) and in presence of **F-H type 2** (*blue*). **F-H**

type 2 binds weakly the carbohydrate-binding site of 5FW LecA as shown by CSP and peak intensity decrease of W42 and W33 resonances, respectively. **D** Shown is a TROSY NMR spectrum of ^{15}N-labeled LecA bound to **F-H type 2**, which perturbs resonances similarly to α-Me-D-galactose (*e.g.* 68, 15 and 75). **D** The magnitude of **F-H type 2** promoted perturbations is lower compared to α-Me-D-galactose as shown in a CSP plot.

Table S1 List of residue IDs in a CSP plot indicating an assigned resonance in ^{15}N-labeled DC-SIGN CRD (n.a. = not assigned)

Residue ID	Assigned resonance
1	241Leu
2	242Val
3	n.a.
4	n.a.
5	n.a.
6	n.a.
7	n.a.
8	n.a.
9	n.a.
10	n.a.
11	n.a.
12	n.a.
13	n.a.
14	n.a.
15	n.a.
16	n.a.
17	n.a.
18	n.a.
19	n.a.
20	260Trp
21	261Thr
22	262Phe
23	263Phe
24	264Gln
25	n.a.
26	266Asn
27	267Cys
28	268Tyr
29	269Phe
30	270Met
31	271Ser
32	n.a.
33	273Ser
34	274Gln
35	275Arg
36	276Asn
37	277Trp
38	278His
39	279Asp
40	280Ser
41	281Ile
42	282Thr
43	283Ala
44	284Cys
45	285Lys
46	286Glu
47	287Val

48	288Gly
49	289Ala
50	290Gln
51	291Leu
52	292Val
53	293Val
54	n.a.
55	295Lys
56	296Ser
57	n.a.
58	298Glu
59	299Glu
60	300Gln
61	301Asn
62	302Phe
63	n.a.
64	304Gln
65	305Leu
66	306Gln
67	307Ser
68	308Ser
69	n.a.
70	310Ser
71	311Asn
72	312Arg
73	313Phe
74	n.a.
75	315Trp
76	316Met
77	317Gly
78	318Leu
79	319Ser
80	320Asp
81	321Leu
82	322Asn
83	323Gln
84	324Glu
85	n.a.
86	326Thr
87	327Trp
88	328Gln
89	329Trp
90	330Val
91	331Asp
92	332Gly
93	333Ser
94	n.a.
95	335LeuH
96	336LeuH
97	n.a.
98	n.a.

99	339Phe
100	340Lys
101	341Gln
102	342Tyr
103	343Trp
104	344Asn
105	n.a.
106	346Gly
107	347Glu
108	n.a.
109	n.a.
110	n.a.
111	n.a.
112	352Gly
113	n.a.
114	n.a.
115	n.a.
116	356Cys
117	357Ala
118	358Glu
119	n.a.
120	360Ser
121	361Gly
122	n.a.
123	363Gly
124	364Trp
125	365Asn
126	366Asp
127	367Asp
128	368Lys
129	369Cys
130	370Asn
131	371Leu
132	372Ala
133	373Lys
134	374Phe
135	375Trp
136	376Ile
137	377Cys
138	378Lys
139	379Lys
140	380Ser
141	381Ala
142	382Ala
143	383Ser
144	n.a.
145	n.a.
146	386Arg
147	n.a.
148	388Glu
149	n.a.

150	390Gln
151	n.a.
152	392Leu
153	393Ser
154	n.a.
155	n.a.
156	n.a.
157	397Ala
158	398Thr

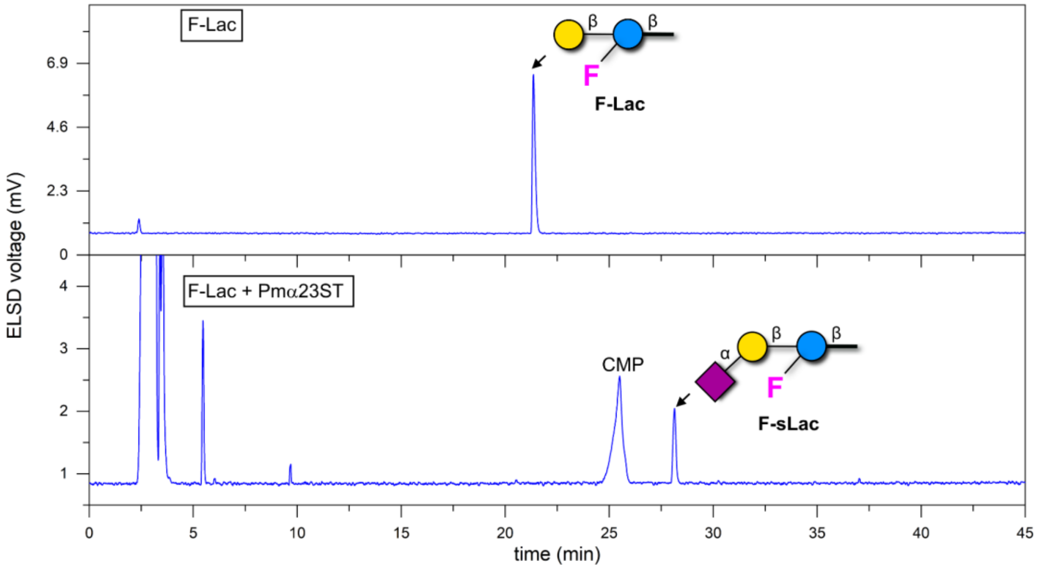

Figure S9 *Enzymatic sialylation of* ***F-Lac*** *with Pmα23ST confirmed by HPLC.* RP-HPLC trace (ELSD trace, Method A) shows consumption of the starting material (**F-Lac**, t_R = 21.4 min, *top*) and formation of a new peak in the crude reaction mixture after 246 min (**F-sLac**, t_R = 28.1 min, *bottom*). HPLC retention times match previously reported data.[17]

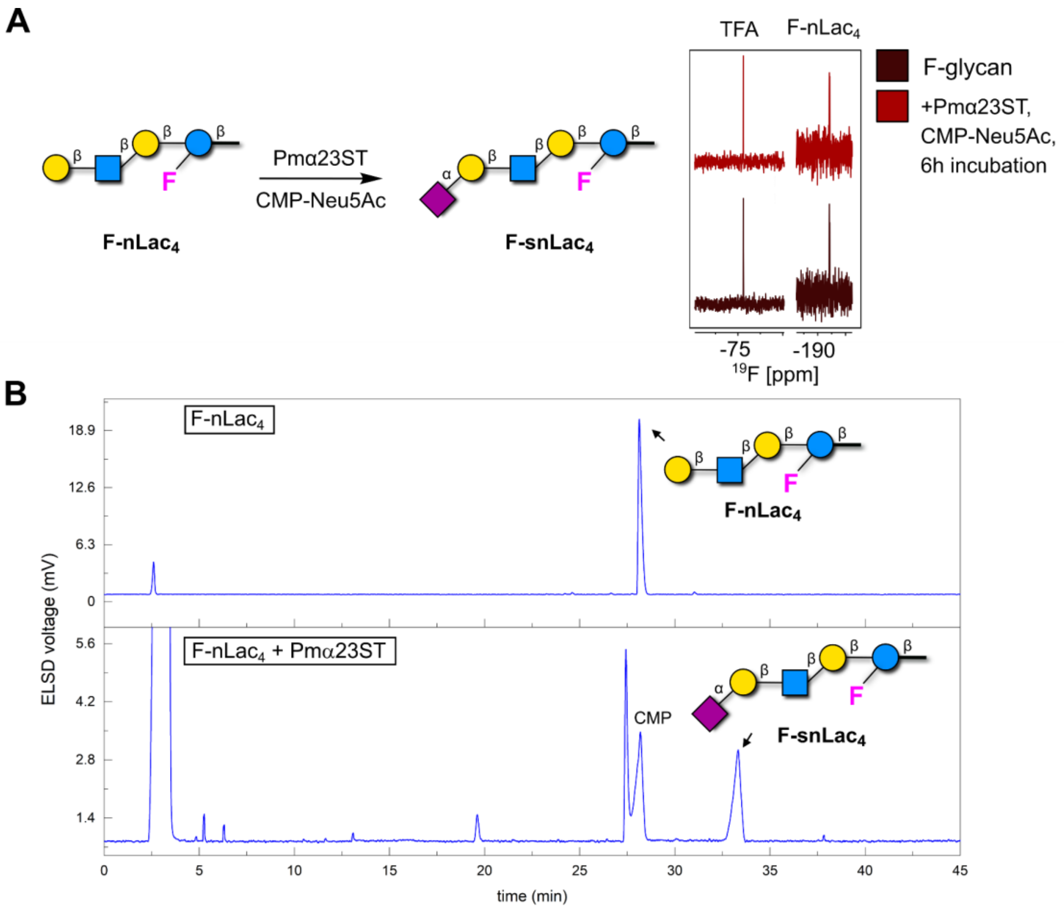

Figure S10 *Enzymatic sialylation of **F-nLac** with Pmα23ST.* **A** Real-time ^{19}F NMR monitoring of **F-nLac$_4$** incubated with Pmα23ST in the presence of CMP-Neu5Ac. No chemical shift perturbation was observed after 6 h incubation time. **B** RP-HPLC trace (ELSD trace, Method A) shows incomplete consumption of the starting material (**F-nLac$_4$**, t_R = 28.1 min, *top*) and formation of the sialylated product (**F-snLac$_4$**, t_R = 33.3 min, *bottom*) in the crude reaction mixture.

E. References

(1) Yu, Y.; Tyrikos-Ergas, T.; Zhu, Y.; Fittolani, G.; Bordoni, V.; Singhal, A.; Fair, R. J.; Grafmüller, A.; Seeberger, P. H.; Delbianco, M. Systematic Hydrogen-Bond Manipulations To Establish Polysaccharide Structure-Property Correlations. *Angew. Chem., Int. Ed.* **2019**, *58* (37), 13127–13132. https://doi.org/10.1002/anie.201906577.

(2) Guberman, M.; Bräutigam, M.; Seeberger, P. H. Automated Glycan Assembly of Lewis Type I and II Oligosaccharide Antigens. *Chem. Sci.* **2019**, *10* (21), 5634–5640. https://doi.org/10.1039/C9SC00768G.

(3) Le Mai Hoang, K.; Pardo-Vargas, A.; Zhu, Y.; Yu, Y.; Loria, M.; Delbianco, M.; Seeberger, P. H. Traceless Photolabile Linker Expedites the Chemical Synthesis of Complex Oligosaccharides by Automated Glycan Assembly. *J. Am. Chem. Soc.* **2019**, *141* (22), 9079–9086. https://doi.org/10.1021/jacs.9b03769.

(4) Gude, M.; Ryf, J.; White, P. D. An Accurate Method for the Quantitation of Fmoc-Derivatized Solid Phase Supports. *Lett. Pept. Sci.* **2002**, *9* (4), 203–206. https://doi.org/doi.org/10.1023/A:1024148619149.

(5) Hurevich, M.; Kandasamy, J.; Ponnappa, B. M.; Collot, M.; Kopetzki, D.; McQuade, D. T.; Seeberger, P. H. Continuous Photochemical Cleavage of Linkers for Solid-Phase Synthesis. *Org. Lett.* **2014**, *16* (6), 1794–1797. https://doi.org/10.1021/ol500530q.

(6) Denavit, V.; Lainé, D.; Bouzriba, C.; Shanina, E.; Gillon, É.; Fortin, S.; Rademacher, C.; Imberty, A.; Giguère, D. Stereoselective Synthesis of Fluorinated Galactopyranosides as Potential Molecular Probes for Galactophilic Proteins: Assessment of Monofluorogalactoside–LecA Interactions. *Chem. – A Eur. J.* **2019**, *25* (17), 4478–4490. https://doi.org/10.1002/chem.201806197.

(7) Sommer, R.; Hauck, D.; Varrot, A.; Wagner, S.; Audfray, A.; Prestel, A.; Möller, H. M.; Imberty, A.; Titz, A. Cinnamide Derivatives of <scp>d</Scp> -Mannose as Inhibitors of the Bacterial Virulence Factor LecB from *Pseudomonas Aeruginosa*. *ChemistryOpen* **2015**, *4* (6), 756–767. https://doi.org/10.1002/open.201500162.

(8) Audfray, A.; Claudinon, J.; Abounit, S.; Ruvoën-Clouet, N.; Larson, G.; Smith, D. F.; Wimmerová, M.; Le Pendu, J.; Römer, W.; Varrot, A.; Imberty, A. Fucose-Binding Lectin from Opportunistic Pathogen Burkholderia Ambifaria Binds to Both Plant and Human Oligosaccharidic Epitopes. *J. Biol. Chem.* **2012**, *287* (6), 4335–4347. https://doi.org/10.1074/jbc.M111.314831.

(9) Shanina, E.; Siebs, E.; Zhang, H.; Varón Silva, D.; Joachim, I.; Titz, A.; Rademacher, C. Protein-Observed 19F NMR of LecA from Pseudomonas Aeruginosa. *Glycobiology* **2020**, *2020*, 1–7. https://doi.org/10.1093/glycob/cwaa057.

(10) Hanske, J.; Aleksić, S.; Ballaschk, M.; Jurk, M.; Shanina, E.; Beerbaum, M.; Schmieder, P.; Keller, B. G.; Rademacher, C. Intradomain Allosteric Network Modulates Calcium Affinity of the C-Type Lectin Receptor Langerin. *J. Am. Chem. Soc.* **2016**, *138* (37), 12176–12186. https://doi.org/10.1021/jacs.6b05458.

(11) Aretz, J.; Baukmann, H.; Shanina, E.; Hanske, J.; Wawrzinek, R.; Zapol'skii, V. A.; Seeberger, P. H.; Kaufmann, D. E.; Rademacher, C. Identification of Multiple Druggable Secondary Sites by Fragment Screening against DC-SIGN. *Angew. Chem., Int. Ed.* **2017**, *56* (25), 7292–7296. https://doi.org/10.1002/anie.201701943.

(12) Carr, H. Y. Steady-State Free Precession in Nuclear Magnetic Resonance. *Phys. Rev.* **1958**, *112* (5), 1693–1701. https://doi.org/10.1103/PhysRev.112.1693.

(13) Meiboom, S.; Gill, D. Modified Spin-Echo Method for Measuring Nuclear Relaxation Times. *Rev. Sci. Instrum.* **1958**, *29* (8), 688–691. https://doi.org/10.1063/1.1716296.

(14) Delaglio, F.; Grzesiek, S.; Vuister, G.; Zhu, G.; Pfeifer, J.; Bax, A. NMRPipe: A Multidimensional Spectral Processing System Based on UNIX Pipes. *J. Biomol. NMR* **1995**, *6* (3), 277–293. https://doi.org/10.1007/BF00197809.

(15) Vranken, W. F.; Boucher, W.; Stevens, T. J.; Fogh, R. H.; Pajon, A.; Llinas, M.; Ulrich, E. L.; Markley, J. L.; Ionides, J.; Laue, E. D. The CCPN Data Model for NMR Spectroscopy: Development of a Software Pipeline. *Proteins Struct. Funct. Genet.* **2005**, *59* (4), 687–696. https://doi.org/10.1002/prot.20449.

(16) Williamson, M. P. Using Chemical Shift Perturbation to Characterise Ligand Binding. *Prog. Nucl. Magn. Reson. Spectrosc.* **2013**, *73*, 1–16. https://doi.org/10.1016/j.pnmrs.2013.02.001.

(17) Fair, R. J.; Hahm, H. S.; Seeberger, P. H. Combination of Automated Solid-Phase and Enzymatic Oligosaccharide Synthesis Provides Access to α(2,3)-Sialylated Glycans. *Chem. Commun.* **2015**, *51* (28), 6183–6185. https://doi.org/10.1039/C5CC01368B.
